# TABASCO: A single molecule, base-pair resolved gene expression simulator

**DOI:** 10.1186/1471-2105-8-480

**Published:** 2007-12-19

**Authors:** Sriram Kosuri, Jason R Kelly, Drew Endy

**Affiliations:** 1 Department of Biological Engineering, Massachusetts Institute of Technology, 77 Massachusetts Ave., Cambridge, MA 02139 USA

## Abstract

**Background:**

Experimental studies of gene expression have identified some of the individual molecular components and elementary reactions that comprise and control cellular behavior. Given our current understanding of gene expression, and the goals of biotechnology research, both scientists and engineers would benefit from detailed simulators that can explicitly compute genome-wide expression levels as a function of individual molecular events, including the activities and interactions of molecules on DNA at single base pair resolution. However, for practical reasons including computational tractability, available simulators have not been able to represent genome-scale models of gene expression at this level of detail.

**Results:**

Here we develop a simulator, TABASCO , which enables the precise representation of individual molecules and events in gene expression for genome-scale systems. We use a single molecule computational engine to track individual molecules interacting with and along nucleic acid polymers at single base resolution. Tabasco uses logical rules to automatically update and delimit the set of species and reactions that comprise a system during simulation, thereby avoiding the need for a priori specification of all possible combinations of molecules and reaction events. We confirm that single molecule, base-pair resolved simulation using TABASCO (Tabasco) can accurately compute gene expression dynamics and, moving beyond previous simulators, provide for the direct representation of intermolecular events such as polymerase collisions and promoter occlusion. We demonstrate the computational capacity of Tabasco by simulating the entirety of gene expression during bacteriophage T7 infection; for reference, the 39,937 base pair T7 genome encodes 56 genes that are transcribed by two types of RNA polymerases active across 22 promoters.

**Conclusion:**

Tabasco enables genome-scale simulation of transcription and translation at individual molecule and single base-pair resolution. By directly representing the position and activity of individual molecules on DNA, Tabasco can directly test the effects of detailed molecular processes on system-wide gene expression. Tabasco would also be useful for studying the complex regulatory mechanisms controlling eukaryotic gene expression. The computational engine underlying Tabasco could also be adapted to represent other types of processive systems in which individual reaction events are organized across a single spatial dimension (e.g., polysaccharide synthesis).

## Background

Mechanistic models of the individual biochemical events that comprise gene expression are quite detailed and are continuously improving. For example, experiments studying the kinetics of promoter initiation and single molecule studies of processive protein movements along DNA have revealed intricate regulatory processes that control gene expression [1-4]. As these details accrue, understanding how individual processes work together to determine system-level behavior becomes increasingly difficult to intuit [5]. The ability to simulate events on and along DNA at base-pair resolution would benefit those interested in studying or attempting to control the consequences of molecular processes on system behavior. However, while currently available simulation techniques are sufficient to study the expression from a single operon at base-pair resolution, they become computational expensive if the system is of much greater scale or complexity [6]. Also, existing simulators cannot formally account for intermolecular events along DNA, such as collisions between polymerases.

There are several mathematical approaches for computing the dynamics of systems of biochemical reactions. For example, the chemical species that define a system can be modeled as continuous variables that change over time. In this "continuous" approach, reactions between chemical species are modeled as a set of coupled ordinary differential equations (ODEs) [7,8]. The set of ODEs are often numerically integrated using established algorithms to compute the dynamics of the system. As a second example, a system's chemical species can be treated as discrete variables that change over time. In this "discrete" approach, reactions between species are treated as individual events that update the system and can be combined into the chemical master equation [9]. The chemical master equation is usually computationally integrated to determine the time evolution of the system using stochastic simulation algorithms (SSAs). SSAs have some theoretical advantages over continuous formulations [10]. For instance, in some systems, an individual reaction event can cause a large difference in the likelihood that other reactions will occur, and so the precise order and timing of individual reaction events can influence overall system behavior. This situation may occur when the numbers of any particular reactant in a system are low, as in the case of a single copy of DNA bound by a protein. In these cases, SSAs provide exact calculations of the system dynamics, while the continuous approximations break down.

However, challenges exist in using either approach to study high-resolution models of gene expression. Both discrete and continuous approaches to solving the time evolution of biochemical reactions share the so-called "combinatorial explosion" problem, in which the number of possible states that need to be enumerated in a system becomes exceedingly large [11]. For example, the spatial and temporal control of gene *endo16 *expression during *S. purpuratu *development is controlled by a 2,300 base-pair (bp) sequence [12]. This control sequence contains 33 sites that bind 15 distinct proteins [13]. Developing a model that fully enumerates all the possible states of the *endo16 *gene regulatory region requires stating over 10^13 ^distinct protein:DNA species. Not only is this computational expensive, it seems unnecessary; the number of possible species far exceeds the copy numbers of the relevant DNA and protein molecules in the cell.

Two approaches have addressed the combinatorial explosion problem with respect to protein complex formation and modification during signaling [11,14,15]. The first approach, single-molecule simulation, taken by the StochSim simulator, tracks individual molecules and their state (e.g., what other molecules they are bound to) so that only the complexes formed at any given time are enumerated (and not all possible complexes) [11]. StochSim allows individual molecules to transition between states (e.g., chemical modification of a protein or complex formation), which in turn modulates the reaction rates of processes that the molecules participate in. These transition and state changes are specified at the start of simulation. Single-molecule simulators are computationally advantageous when the number of tracked molecules is much smaller than the number of possible states that the system can potentially achieve. A second approach, taken by the Molecularizer simulator, is to dynamically generate reactions as they occur based on automatic adjustments to reaction rates based on diffusion considerations [15]. Moleculizer can construct the set of reactants and reactions that are actually executed during simulation and can use this more manageable set of reactions as input into more traditional simulators. Dynamic reaction generation simulators such as Moleculizer work well when many of the possible states of the system are never realized, and thus the simulators need only ever represent a fraction of the total possible system.

StochSim and Moleculizer were designed to model the binding and unbinding of proteins within complexes. However, these simulators are not well suited for modeling systems in which components within a complex are processive (i.e., components transitioning among many binding states) or when the number of binding states is large. For example, simulating a single RNA polymerase transcribing a 3,000 bp gene at single-base resolution using a single-molecule simulator would require a model for a DNA molecule comprised of over 3,000 different states with enumerated rules for the transitions from one base to the next. Using a dynamic reaction generation simulator, such as Moleculizer, would not lead to significant speed enhancements because most of the states of the system are reached during a simulation, albeit rarely.

Recent efforts to stochastically simulate gene expression have focused on reducing the number of states and complexity of the system being studied by approximating the kinetic delays that occur during transcriptional and translational elongation processes. For example, the stochastic simulator Dizzy allows for modeling transcription elongation as a series of equivalent steps that can be sampled in total from the gamma distribution, as Gibson and Bruck suggest [16,17]. However, these simplifications cannot account for the effects that intermolecular interactions on the DNA may have on system dynamics. In addition, Roussel and Zhu approximated the delays in transcription that can take into account polymerase interactions using a reduced site-oriented Markov model that is accurate in cases where there are limited polymerase interactions [18,19]. However, without a priori knowledge for how polymerase interactions affect the expression of any particular gene, especially in the case of multi-promoter, multi-gene, multi-polymerase systems, we must use a more detailed model of gene expression.

Thus, in order to efficiently and more realistically simulate base-pair resolved models of gene expression, we developed a single-molecule stochastic simulator, Tabasco, optimized to handle molecular events specific to gene expression such as the initiation, elongation and termination of transcription and translation as well as interactions among protein-DNA complexes. Tabasco tracks individual molecules and reactions on the DNA at single base resolution (e.g., RNA polymerase transcribing DNA) in order to avoid the specification of many unoccupied system states. Tabasco also uses dynamic reaction generation based on encoded rules for gene expression in order to avoid specification of all possible states and transitions within the system (e.g., all RNA polymerase molecules transcribe DNA to produce RNA). This framework provides the accurate descriptions of gene expression dynamics while allowing analysis of phenomena such as how intermolecular events between DNA-protein complexes affect system-wide gene expression.

Our motivations for creating Tabasco started from our interest in simulating the dynamics of bacteriophage T7 gene expression during phage infection. For example, past models of T7 infection used ODEs to simulate T7 gene expression dynamics. The use of ODEs limited our ability to accurately simulate the kinetics of T7 gene expression and study how intermolecular events may impact the genome-wide allocation of expression resources [20,21]. For example, the *E. coli *RNA polymerase initiates expression of early T7 genes, including the gene encoding T7 RNA polymerase. Thus, newly synthesized T7 RNA polymerase first initiates transcription behind already transcribing *E. coli *RNA polymerase molecules. *E. coli *RNA polymerase has a ~45 nucleotide per second elongation rate; T7 RNA polymerase has a ~250 nucleotide per second elongation rate. What happens when a T7 RNA polymerase molecule overtakes an *E. coli *RNA polymerase is not well understood. However, the transition from *E. coli *to T7 RNA polymerase mediated genome entry will impact the timing of expression across the genome; cell-cell variation in the entry transition will limit the precision by which T7 can control infection. Thus, simulating possible polymerase-polymerase interaction models is an interesting research question. Using Tabasco, we can explore the dynamics of gene expression during T7 development at single-base resolution. In the case of T7, the increased modeling resolution afforded by Tabasco allows for direct evaluation of assumptions concerning polymerase interactions, transcriptional coupling to genome entry, and stochastic fluctuations on phage development. Although motivated by our interest in T7, Tabasco can also be used to represent other genetic systems and, as such, is generally useful to those interested in understanding how detailed molecular processes affect genome-wide gene expression.

## Results

### Algorithm

Tabasco is a stochastic simulator that tracks individual molecules of DNA and associated proteins at single base-pair resolution. Tabasco makes use of a Gibson-accelerated Gillespie SSA to compute the reaction event timing and the resultant time-evolution of the genetic system [17]. Tabasco uses predefined rules of transcription and translation such as initiation, elongation, termination, and protein interactions of polymerases, ribosomes, and other DNA/RNA-associated proteins. Based on these rules, Tabasco automatically updates the states of molecules and reaction events (Methods). For example, if a transcribing RNA polymerase temporarily occupies the DNA-binding site for a second protein, the simulator makes the site unavailable for binding until the polymerase is no longer occluding access to the binding site.

**Figure 1:**
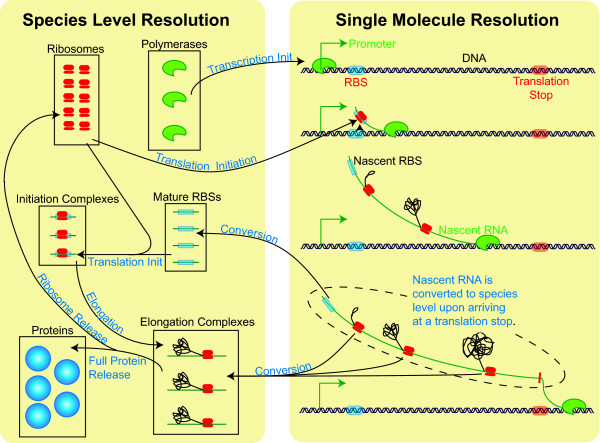
**Structure of TABASCO simulator**. A simplified flow diagram of gene expression shows how Tabasco transitions from tracking individual molecules (Single Molecule Resolution) to grouping them into species (Species Level Resolution). The arrows represent reactions that can occur (process labeled in blue).

Tabasco transitions between two levels of resolution while simulating gene expression: "single-molecule" and "species level" (Figure 1). Reactants and events that occur on the DNA are tracked at single-molecule resolution–each copy of DNA and proteins associated with them are tracked individually by the simulator. In this regime, events such as binding/un-binding and polymerase movements are dynamically generated based on the current state of the DNA molecules and proteins on the DNA. On the other hand, the species-level resolution is akin to traditional SSAs in which reactants are tracked as groups of equivalent species. For example, Tabasco tracks all cellular protein-protein interactions at the species level.

**Figure 2:**
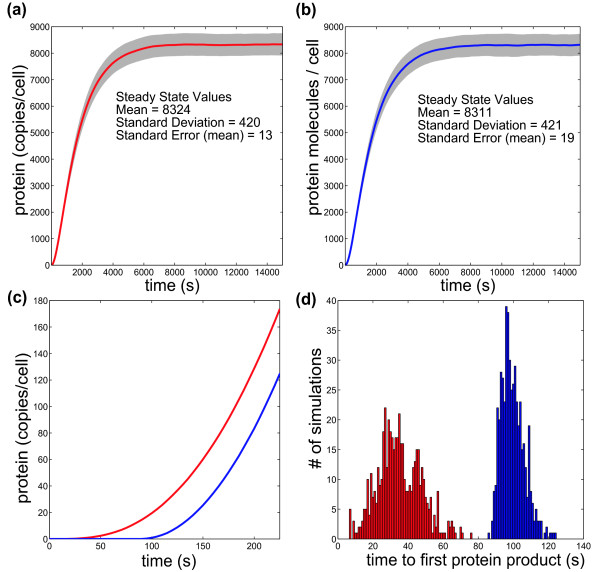
**Differences in single-step and multi-step elongation processes**. Simulating the elongation of RNA polymerase along 50 based of DNA as a one-step elongation process versus a chain of individual elongation events gives the same average elongation time, but produces marked differences in the distribution of those elongation times. The process shown here is either a single 1.0 second elongation step (red) or fifty 0.02 second elongation steps (blue). Each histogram is the distribution of resulting times from 20,000 independent simulations.

The transition between the single-molecule level and the species level occurs during the tracking of RNA abundances (Figure 1). Only those RNA molecules that are still attached to transcribing RNA polymerases are tracked as single molecules. As each coding domain on an RNA molecule is completed, it becomes part of the species-level simulation. At the species-level, each of the coding domains is treated separately as a species, and ribosomes that initiate translation on a coding domain are assumed not to interfere with one another. At both the single-molecule and species-level, translation is treated as a series of single amino acid polymerization steps, with the number of steps depending on the length of the coding domain. This gives a more accurate distribution of times for protein production than treating the whole elongation process as a single step. Based on the work of Gibson and Bruck, we compute translation steps in aggregate using the gamma distribution (Figure 2) [17].

**Figure 3:**
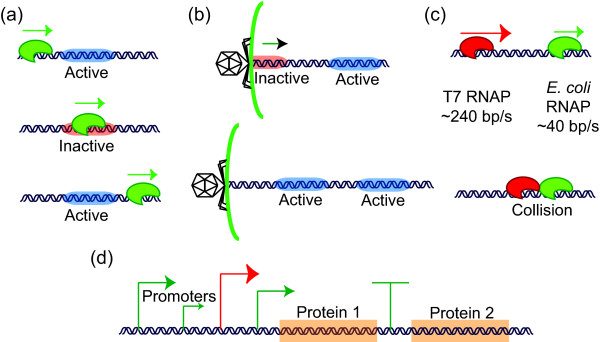
**Advantages of single molecule resolution**. Tracking individual molecules of DNA at a base-pair resolution allows for creation of implicit rules defined by the state of protein-DNA interactions and of the DNA itself. For example, when a traversing polymerase occupies a genetic element such as a protein-DNA binding site (a), the binding site is inactivated and prevented from binding a protein until the polymerase completely clears the site. Activation of genetic elements by entry of DNA into the cell (b) and inter-polymerase interactions (c) can be directly simulated as well. Finally, in complex genetic environments (d) where multiple promoters and terminators regulate the expression of genes, a priori transcription levels for each protein need not be calculated, for they are generated implicitly.

The structure of Tabasco confers at least four advantages. First, treating gene expression at base-pair resolution allows for more accurate representation of the kinetics of gene expression. For example, traditional SSAs often lump multi-step reactions as single steps causing inaccurate estimates on pre-steady state kinetics. Second, tracking the state of individual proteins on DNA and allowing internal logic to automatically generate reactions eliminates the need to enumerate all the possible states of polymerases and proteins associated with the DNA. For example, transcribing polymerases and processes such as genome entry into a cell can cause certain protein binding sites to be inaccessible (Figure 3a, b). This feature also allows us to consider and integrate many factors that may influence the rate of RNA polymerization for any particular gene, such as the binding of multiple transcription factors or the contribution of RNA polymerases that initiated transcription at a promoter connected to an upstream gene (Figure 3d). Third, protein-protein interactions that may occur on DNA, such as collisions between different polymerases, can be accounted for and simulated based on simple and explicit rules (Figure 3c). Fourth, Tabasco can be used to graphically depict the location and dynamics of individual RNA polymerase molecules transcribing DNA, providing a useful visual tool for considering genome-scale gene expression dynamics.

### Testing

#### Simple gene expression models

**Figure 4:**
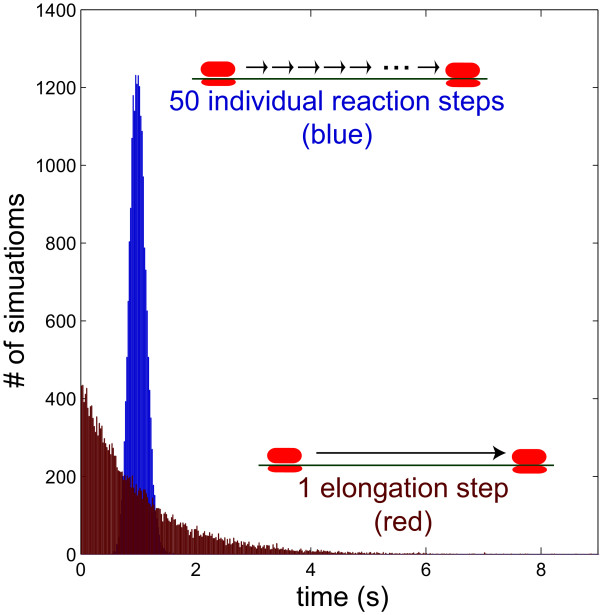
**Comparison of TABASCO to species-level simulations for a simple gene**. We simulated the expression of a 1,000 amino acid protein using Tabasco (blue), and a standard species-level simulator (red) in which the processes of elongation (transcription and translation) were lumped into single steps. While the steady-state averages and standard deviations (a, b) are statistically equivalent, the pre-steady state kinetics (c) are quite different. Production of the first protein product (d) is much faster in the standard species-level simulator than in Tabasco. In fact, the species-level simulator generates unrealistic times to first production of a a protein; elongation of the polymerase alone should take ~75 seconds for a gene of this size (see text). The data shown are averages of 500 individual simulations.

As an initial test, we simulated the expression of a 1,000 amino acid protein using both Tabasco and a standard species-level SSA (Figure 4). Both simulators used identical gene expression models except for one difference. In the standard SSA, processive transcription and translation elongation reactions along the DNA are treated as a lumped, single-step reaction sampled from an exponential distribution; in Tabasco these reactions are treated as a series of individual base-pair elongation steps. In addition, due to internal structural differences between the two simulators, the two underlying models had slightly different rate constants for RNA polymerase clearance from the promoter region (Methods). Thus, the clearance rate was adjusted in the species-level SSA to produce equal steady state levels of protein to the Tabasco simulation (Table 1). At steady state, both simulators produce statistically equivalent results (Figure 4a, b). However, an expected difference arises in the pre-steady state dynamics of the system (Figure 4c). The species-level SSA simulation first produces protein by ~30 seconds (Figure 4d). This is unrealistic; *E. coli *RNA polymerase transcribes at an average elongation rate of 40 bp per second, production of a 3,000 nucleotide messenger RNA should take ~75 seconds. Thus, even if a ribosome directly followed the first transcribing RNA polymerase, protein production should not take less time than RNA production. Tabasco produces protein only after a more accurate ~100 seconds (Figure 4d).

**Table 1:** Constants used in the Simple Gene Expression Model

**Constant**	**Tabasco**	**Species-Level**
*kon *(M^-1^s^-1^).	4E7	4E7
*koff *(s^-1^).	4	4
*kiniton *(s^-1^).	1.2	1.2
*% runoff*	0	N/A
*krecyc *(s^-1^).	0	N/A
*kelong *(s^-1^)	0.23	0.23
*kprot *(s^-1^)	0.645	0.645
*kribon *(M^-1^s^-1^)	1.15E4	1.15E4
*kclear *(s^-1^).	0.14*	0.1308
*rib elongation *(s^-1^)	20	20
*mRNA deg *(s^-1^).	2.5E-3	2.5E-3
*protein deg *(s^-1^)	7E-4	7E-4

We compared two models of expression of a single-gene varying only if mRNA and protein elongation reactions are treated as series of individual steps or a single lumped reaction (Figure 3; Figure 6). The kclear rates do not exactly match because of small differences in the model structures (see text). The Tabasco constant for *kclear *was adjusted to ensure the steady-state levels of mRNA and protein were equivalent in both simulations.

The discrepancy in the time for synthesis of a first protein product occurs for two reasons [17]. First, since the RNA polymerase and ribosome elongation steps in the species-level simulation are treated as single exponential elementary reactions, 63% of the reactions occur prior to the average reaction time (Figure 2). The second and more substantive reason is that SSAs assume that reactions are Markovian (i.e., reaction event timing only depends on the current state of the system, and not system history). In an SSA that treats elongation as a single step, a ribosome that has initiated translation has some non-zero chance of completing protein synthesis; since the SSA treats the reaction as Markovian, the greater the number of ribosomes that have initiated translation, the greater the chance that any single protein synthesis event will be completed. Thus, if many ribosomes initiate translation before any one ribosome has enough time to complete translation of a coding sequence, then then protein synthesis will be computed at completing in an unrealistically short time. This is physically unrealistic; lumped reactions representing processive reactions should not be modeled as Markovian. In other words, a ribosome that begins translation should not affect the speed at which downstream ribosomes will complete protein synthesis. The fact that Tabasco treats each elongation reaction as a series of elongation steps alleviates both of these problems. As a result, Tabasco provides a more accurate estimate of pre-steady state gene expression kinetics than simulators and models that lump transcription and translation processes into a single reaction. Accurate calculation of the pre-steady state kinetics is important for systems in which a steady state is never reached, such as during phage infection, cell cycle, or animal development, and is the reason that many groups have incorporated approximate delays into their simulation frameworks [18,19,22-27].

#### Polymerase Interactions

The first natural biological system that we studied using Tabasco is bacteriophage T7. During bacteriophage T7 infection, the *E. coli *RNA polymerase initiates expression of early T7 genes, including T7 RNA polymerase [28]. T7 RNA polymerase first initiates transcription behind already transcribing *E. coli *RNA polymerase molecules. At 37°C, *E. coli *RNA polymerase transcribes at ~45 bp per second; T7 RNA polymerase transcribes at ~250 bp per second [29]. Thus, T7 RNA polymerases will overtake *E. coli *RNA polymerases. How transcribing RNA polymerase molecules interact mechanistically is just beginning to be understood, but experimental studies show that these interactions are important [30-33].

**Figure 5:**
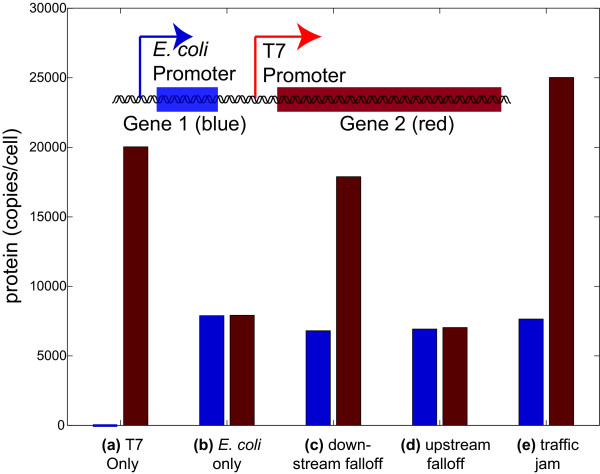
**Simulating models for inter-polymerase interactions**. Average steady-state protein levels from simulations of the DNA molecule with promoters for different polymerases (T7 and *E. coli *RNA polymerases) transcribing two genes (inset). The two polymerases are expected to interact since the T7 RNA polymerase is ~5 times faster than the *E. coli *RNA polymerase,. The graph shows different calculated steady-state levels of the two reporter proteins given different models for the interactions between the two polymerases. The (a) 'T7 only' and (b) '*E. coli *only' are controls that show expression without *E. coli *and T7 RNA polymerase, respectively. The 'downstream falloff' model (c) specifies that, upon a collision between two transcribing polymerases, the downstream polymerase will prematurely terminate transcription and release from the DNA. The 'upstream falloff' model (d) describes the opposite situation when the upstream polymerase falls off. Finally, the 'traffic jam' model (e) specifies that the upstream polymerase follows the downstream polymerase at the speed of the downstream polymerase.

In order to test how different models of polymerase-polymerase interactions impact gene expression, we first used Tabasco to simulate gene expression from a reduced genetic system that captures key features from the layout of the T7 genome. Briefly, this two-gene system has an *E. coli *promoter expressing two hypothetical genes encoded on a polycistronic mRNA (encoding Proteins 1 and 2), and a T7 promoter expressing a monocistronic mRNA that only encodes Protein 2 (Figure 5). We used Tabasco to test three distinct models for how co-transcribing RNA polymerases may interact: (1) the downstream polymerase terminates transcription allowing the upstream polymerase to continue (downstream falloff model), (2) the upstream polymerase terminates transcription while the downstream polymerase continues (upstream falloff model), or (3) the upstream polymerase follows at the speed of the downstream polymerase (traffic jam model). Each polymerase collision model leads to different levels of steady state protein production (Figure 5). For example, in the upstream falloff model, most of the T7 polymerases will prematurely terminate because they overtake slower *E. coli *RNA polymerases (Figure 5d). As a result, gene expression levels are similar to the situation where there is no T7 polymerase at all (Figure 5b).

### Application

#### Genome-scale models and simulation

In order to test the effectiveness of Tabasco on genome-scale systems, we simulated gene expression during bacteriophage T7 infection. The 39,937 base pair T7 genome, as represented in our test simulation, is comprised of 52 coding domains, 22 host and phage promoters, two transcriptional terminators, and three distinct transcriptional feedback loops; four T7 coding domains were not represented as they are the result of translational frameshifts or alternative start sites for other genes [28,34]. Because there are relatively few transcriptional terminators and many promoters, the rates of transcription for individual T7 genes depends on the combined levels of transcription initiation from all upstream promoters, as well as the presence of different types of RNA polymerases. In the case of T7, there is a further complication in that not all genes are available for transcription at once, since the entry of the T7 genome is a relatively slow process that is itself mediated by transcribing RNA polymerases.

Using realistic parameter sets, we simulated a single cell being simultaneously infected by three phage particles (Methods). The protein-DNA interactions on the DNA were tracked independently for each infecting phage, but use the same pools of soluble proteins in the cell; thus, these simulations track a combined 119,811 base pairs of DNA. We incorporated known mechanisms of cellular entry of T7 DNA into our simulations. We also developed a method to visualize the results of our simulations that allows for the graphical display of dynamic Tabasco output (Methods). Simulation of a full-scale genomic model of T7 gene expression on a single AMD Athlon MP 2100+ 1.8 GHz processor takes between 1–2 hours for a model that represents 30 minutes of real-time infection. Visualization of Tabasco output displays both T7 and *E. coli *RNA polymerases transcribing the DNA, the extent of genome entry, and the resulting mRNA and protein levels from the 52 encoded genes (Additional file 1). Over the course of the simulation, the phage DNA enters and is transcribed by the *E. coli *RNA polymerase. As the T7 RNA polymerase is produced, host transcription is attenuated while the T7 RNA polymerase takes over transcription and entry of the remaining T7 genes. For the first time, we are able to simulate gene expression at single base resolution for all gene expression in a particular organism during its development. In turn this allows us to test specific hypotheses of polymerase interactions, stochastic gene expression, and coupling of entry and transcription. An experimental analysis of the T7 gene expression program, using Tabasco, will be presented in a forthcoming paper [Keller H, Endy D, Kosuri S, *in preparation*].

## Discussion & Conclusion

We designed and developed Tabasco to revisit approximations encoded within previously available gene expression simulation algorithms, and to develop a method for efficiently computing single-molecule, base-pair resolved models of gene expression. Previous tools required either modeling smaller systems at increased resolution, supercomputers, or simplifications and assumptions of the biophysical models that result in reduced simulation accuracy. For example, Tabasco's implementation of a Gibson-accelerated Gillespie SSA allowed us to bypass simplifications that are often made for promoter binding by RNA polymerase and transcription initiation [18-20,35]. We have shown here that the increased resolution of Tabasco provides more accurate pre-steady state kinetics of gene expression, gives us the ability to test models of polymerase and protein interactions on the DNA, and allows us to fully simulate the dynamics of gene expression during development of bacteriophage T7 at base-pair resolution.

Like Moleculizer, Tabasco dynamically generates reactions based on rules. Specifically, Tabasco uses the mechanics of gene expression as rules in order to avoid specification of all the states and transitions prior to simulation [15]. Like Stochsim, Tabasco is a single-molecule simulator, which allows Tabasco to track the state of individual molecules of DNA, rather than tracking individually all states of a system that could be reached [11]. As a result, Tabasco differs from previous gene expression simulators in that it is able to automatically represent the effects of molecular collisions, such as polymerase-polymerase interactions and the occlusions of DNA elements by transiting molecules.

Since Tabasco tracks individual molecules of DNA, the processing power required for Tabasco simulation scales with the copy number and length of the template DNA. While requiring significant processing power, Tabasco is computationally tractable in regimes for which stochastic simulations are often needed to produce accurate results (for example, when the number of DNA molecules in the system is low). Those interested in using simulators similar to Tabasco to study detailed interactions at the RNA level would face challenges due to the higher numbers of RNA compared to DNA in the cell. Such challenges may be overcome in the future by incorporating the work of others on increasing the efficiency of SSAs without significantly sacrificing accuracy [36-39]. In particular, if the probabilistic simplifications of Russell and Zhu can be extended to simulations in larger, more complex systems such as T7, we would expect to see increases in simulation efficiency [19].

While Tabasco is an exact SSA, it only exactly simulates the kinetics of the already simplified models that we give it. For example, we model transcription elongation as a first-order reaction, even though we know that this process is more complicated [40]. In addition, the general use of physics models based on well-mixed elementary chemical reactions may sometimes be an inappropriate approximation of the inside of a cell. For example, it is known that the binding of proteins interacting with DNA often involves one-dimensional diffusion along a DNA template [41,42]. The effects of these simplifications on simulation accuracy are not well studied, and are common across currently available simulators of gene expression dynamics. Finally, other processes that affect transcription such as mRNA secondary structure, sequence-specific kinetics, regulated pausing and backtracking, and many other known biophysical phenomena are not considered here. Such processes can and should be incorporated into gene expression simulation frameworks as the particular biological system being studied or engineered warrants.

Executables, source code, documentation, and usage notes for Tabasco are freely available, and should facilitate future extensions on the current design (Availability and Requirements). We have already found Tabasco to be useful in constraining models of T7 gene expression by comparing simulator output to new experimental measurements of absolute copies of mRNA abundance during infection; these results will be presented in a forthcoming paper [Keller S, Endy D, Kosuri S, *in preparation*]. In addition, Tabasco should be a good base to further study interactions on DNA that lead to transcription and translation. For example, Tabasco provides a platform to explicitly simulate hypotheses how many transcription factors can interact to direct eukaryotic gene expression, such as in control of *endo16 *expression [13]. Finally, the general approach of using simulator-encoded logic and tracking of one-dimensional reaction systems should be useful for studying other biological phenomena – for example, oligosaccharide modifications of proteins.

## Methods

### Overall Simulator Structure

Tabasco implements a modified version of the Gibson Next Reaction Method (NRM) [17]. Gibson's NRM is an exact SSA that extends Gillespie's original First Reaction method by (1) updating only the minimum number of reactions through the use of a dependency graph and using absolute tentative reaction times, and (2) using an efficient data structure, the indexed priority queue, to store and sort reactions. At the start of the NRM, all reactions are defined and their tentative time of next execution ("tentative reaction time") is calculated and stored within an indexed priority queue. In addition, a dependency graph, which allows an executed reaction to call an update on only those reactions that are affected, is generated. The use of absolute times when calculating tentative reaction times allows reactions that are not affected by the execution of the last reaction to remain valid for the next iteration. The indexed priority queue sorts these reactions efficiently to allow quick searches for the minimum tentative time as well as quick lookups for any particular reaction. The reaction with the next tentative reaction time is executed, any tentative reaction times that are affected by the execution of the current reaction are updated, and the indexed priority queue structure is reordered to reflect the new times. The NRM does not change the tentative reaction times of reactions that are not affected by the currently executing reaction. The process is repeated to compute the time evolution of the entire system. Gibson and Bruck proved that the NRM is equivalent to the exact SSA algorithms developed by Gillespie.

The NRM uses a dependency graph to determine which reactions are affected by any particular reaction's execution. In the NRM, the dependency graph is constructed only once at the start of simulation and remains unchanged afterwards. However, since Tabasco creates reactions and complexes at the single-molecule level dynamically during simulation, a static dependency graph and indexed priority queue will not work. In order to solve this problem, Tabasco contains two specialized classes per DNA molecule within the overall indexed priority queue that are used to track a set of dynamically generated reactions. Each class contains a dynamic priority queue that stores the dynamically generated transcriptional and translational reactions and their tentative reaction times, as well the dependencies of any particular reaction (these specialized priority queues and the overall indexed priority queue are easy to confuse, and thus we will refer to the prior as the dynamic priority queue). The minimum tentative reaction time for all the dynamic reactions is set as the tentative reaction time of that dynamic priority queue with respect to the overall priority queue. Since, as in the NRM, Tabasco uses absolute times for determining the next reaction, the particular choice of the data structures containing the reactions does not affect the simulation results. The reason to separate the dynamic queues from the main indexed priority queue is to allow the size of the dynamic queues to change over time. As long as all dependencies are accurately updated upon reaction execution in the dynamic priority queues, the results are equivalent to the NRM. The structure of these dynamic priority queues will now be discussed in more detail.

### Transcription

A special class tracks the transcriptional processes for each DNA molecule. This class contains a dynamic priority queue that keeps track of all reactions related to that DNA molecule, such as transcriptional elongation processes. All reactions that are stored in this dynamic priority queue have tentative reaction times (as calculated by the NRM), and the minimum tentative reaction time is used to set when this class should be called to execute within the indexed priority queue. In order to determine what the effects of a particular binding or elongation reaction are, the class contains two arrays where each element represents one DNA base. Each element of the first array contains pointers to transcriptional elements encoded at that location such as promoters and terminators. The second array contains pointers to all DNA-protein complexes that reside on the DNA such as elongating RNA polymerase. Only one transcriptional control element or DNA complex can occupy any particular position at any time.

**Figure 6:**
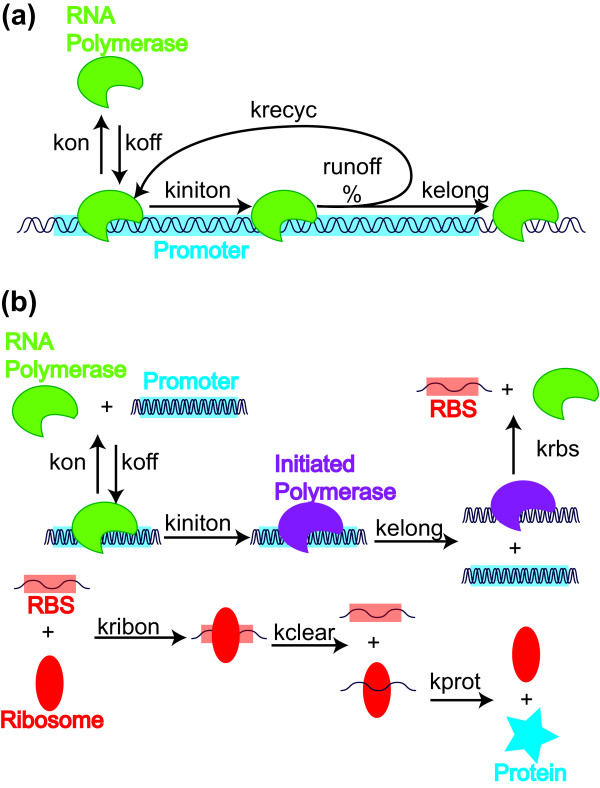
**Model structures for Tabasco and species-level simulations of expression of a single gene**. We simulated expression of a single gene using a standard species-level SSA, and using the Tabasco gene expression simulator (Figure 3). The Tabasco-based simulation uses a transcription initiation model (a) with the rest of gene expression using the general schematic shown in Figure 1. We constructed the model for the species-level simulator (b) to mimic the Tabasco-based model, except that elongation reactions were treated as single-lumped reactions.

The framework we use here to simulate protein-DNA binding is shown in Figure 6A. Interactions from the species level to this specialized reaction occur when proteins bind the DNA. For example, RNA polymerase or other proteins can bind free promoters to form a protein-DNA complex, which causes the DNA complex array to be updated, the number of available promoters and RNA polymerase to decrement, and finally places two reactions into the dynamic priority queue. The two reactions compete to either have the complex form an initiation complex or fall off the DNA. If an initiation complex is formed, a stochastic decision is made as to whether the complex will recycle back to an initiation complex with some characteristic time, or moves on to be become an elongation complex. This stochastic decision is instantaneous in reaction time and will be discussed below. The polymerase can undergo abortive initiation step, recycling to the initiation complex, or form an elongation complex. A promoter is not made available for rebinding until the footprint of an initiating polymerase has cleared the entire promoter region.

Once an elongation complex is formed, each elongation reaction causes the complex to move one base pair, and the elongation reaction is updated with a new time for the next elongation step. When an elongation reaction executes, the algorithm also checks whether another complex's footprint prevents the current polymerase from moving forward by checking the array containing all complex positions on the DNA. If there is another polymerase blocking the current polymerase's path, the polymerases will behave according to the polymerase interaction model chosen at the start of simulation. For example, the upstream polymerase can terminate, or signal the downstream polymerase to terminate the next time the polymerase is set to elongate, or simply hold its position until the next opportunity to elongate. The elongation reaction also does a check for transcriptional elements on the DNA such as promoters and transcriptional terminators. Upon arriving at a transcriptional terminator, a polymerase will either continue transcribing or terminate depending on a stochastic decision that will also be discussed below. If a transcribing polymerase occludes an open promoter, then the promoter will be decremented and unavailable for binding.

The "stochastic decisions" described above for transcriptional termination and abortive initiation on the promoter are used as methods to simplify parameterization in the models, so as to be consistent with how experimental data for such events is typically reported. In the case of termination, a uniform random number is chosen, and if that number is less than the termination efficiency, the polymerase will terminate transcription and fall off the DNA. Analogously, in the case of abortive initiation, if the uniform random number is less than the abortive initiation percentage, then the next reaction will be an abortive initiation step rather than formation of an elongation complex. These decisions take no simulator time, but cause changes in the downstream actions the complexes may take. This simplification is not done for computational efficiency, but more for model simplicity. Tabasco could be modified, with little computational load, to replace the stochastic decisions into competing rates of different reactions. However, these rates are not well understood, and thus we chose to incorporate the measurable models of termination efficiencies and abortive initiation frequencies into the simulator.

### Translation

The transcribing RNA polymerase complexes also produce mRNA, which are tracked by a separate class. Since there are many more copies of RNA than DNA during simulation, and because we were uncertain as to the importance of protein-protein interactions on the mRNA, we chose to treat the majority of translation at the species level. However, if, for example, an RNA polymerase prematurely terminates before reaching the translation stop site, the mRNA and the ribosomes translating it will not produce function proteins. Thus, so long as the coding sequence is still being transcribed, we must treat all translation events at the single-molecule level as well. However, as soon as the entire coding sequence of an open reading frame is transcribed, Tabasco transitions to tracking species of mRNA molecules.

We used two classes to model the formation of RBSs and their respective start sites, the Nascent RBSs and Mature RBSs, in order to differentiate when mRNA should be tracked at the single-molecule level or the species-level, respectively. At the start of simulation, one more array of genome length is created that contains the positions of translation start and stop sites on the DNA. As RNA polymerase elongates (as described above), if the polymerase transcribes past a translational start site, a Nascent RBS is made. This Nascent RBS is available for binding by free ribosomes, and a reaction is automatically created and placed into the dynamic priority queue for translation processes. Once the polymerase arrives at the corresponding translation termination site, the Nascent RBS is converted to a Mature RBS, which is tracked at the species-level in the overall Indexed Priority Queue.

Translation, both at the single-molecule level and species level, occurs in three steps. First, ribosomes can bind either Nascent or Mature RBSs; there is an initial reaction to form the (Nascent or Mature) Initiation Complex. Second, these initiation complexes are converted to (Nascent or Mature) Elongation Complexes and an RBS at a rate that depends on the speed of the ribosome and the length the ribosome must travel to clear the ribosome binding site. This reaction is treated as a single step, however the distribution of times is chosen from a gamma distribution, in order to better represent a series of individual elongation steps. Third, the Elongation Complex then forms a finished protein and a free ribosome at a rate proportional to the remaining length of the open reading frame. This reaction also is computed via a gamma distribution.

At the single molecule level, two additional mechanisms allow for the state of the transcribing RNA polymerase to affect translations. First, if the transcribing RNA polymerase terminates before reaching a corresponding translation stop site, all bound ribosomes are immediately released, the Nascent RBS is removed, and no protein product is formed. Also, for any particular Nascent RNA, the maximal number of Nascent Elongation Complexes that can be formed is capped at the length of the currently transcribed portion of the open reading frame divided by the footprint of the ribosome.

Finally, to note, all transitions between single-molecule tracking and species-level tracking occur when a reaction, such as RNA polymerase termination or Initiation Complex conversion into an Elongation Complex. Thus, the transition from single-molecule tracking back to the species level tracking should also not affect the validity of using the NRM.

### DNA entry

We developed multiple models to represent DNA entry into a cell or compartment (such a step can be useful in starting a simulation of infection or transformation). First, DNA can enter the cell via a zero-order constant reaction rate. Second, RNA polymerases have themselves been implicated as molecular motors that can drive DNA entry [29,43]. Thus, in Tabasco, RNA polymerases that reach the end of a DNA molecule that has not yet fully entered the cell can cause DNA internalization at the rate of transcription elongation. Both of these mechanisms were used during our simulation of T7 gene expression.

### Simulation, Data Output, Visualization, Code

Tabasco is written in Java^® ^1.4. The input file to the simulator is an XML file that describes and parameterizes the relevant genetic elements, initial conditions, and any other reactions that occur. The visualization is created by producing images that are then merged using Quicktime^® ^to create a movie. The source code, executables, along with documentation and instructions for use are available within Additional Files 2, 3 and 4 and via the TABASCO website (Availability & Requirements).

### Parameterization

All constants used are provided for completeness. Please see the Supplementary Materials for input files and exact constants used.

#### Gamma versus Exponential distribution

The distribution of expected times for a reaction to occur in a stochastic simulator depends on the underlying model (Figure 2). Elementary chemical reactions will follow an exponential distribution in arrival times. However, this is not true of non-elementary reactions. Treating an imaginary elongation process as one step versus 50 individual steps has significant consequences. To obtain the distribution time for the two cases, we used uniformly-distributed pseudorandom numbers and transformed them into exponential- or gamma-distributed random numbers. The exponential distribution is used for the single step representation. Exponentially distributed numbers are calculated by simply taking the negative natural log of a uniformly-distributed pseudorandom number. A series of exponentially distributed arrival times, as in the case of the multi-step elongation process, is given exactly by the gamma distribution. Gamma distributed numbers are calculated from uniformly-distributed pseudorandom numbers by an implementation of the rejection method [44]. Pseudorandom numbers are generated from Java's implementation (java.util.Random) of a linear congruential pseudorandom number generator with a 48-bit seed [45].

#### Simple Gene Expression model

We simulated two models of a promoter driving expression of a coding domain. The first model, termed single-molecule simulation, used the described Tabasco simulator to account for each reaction step during transcription and translation. The model uses the schemes shown in Figures 1 and 6a. The second model, termed the species-level simulation, treats transcriptional and translational elongation as a single step as shown in Figure 6b. The main parameters used in both models are shown in Table 1. Constants were adjusted slightly to account for small differences in model structure to give equal steady state values of mRNA and protein levels. Finally, the input files for the simulations can be found in the Supplementary Materials; the input files can be used to either run the simulation using Tabasco, or check all parameters used for the simulation.

#### Polymerase Interactions & Bacteriophage T7

The simulations for the polymerase interactions and bacteriophage T7 development used parameters that can be found in the input files in the Supplementary Materials. Table S1 details the meaning of each of the parameters in the input files. The parameters used were based on empirical measurements where possible. However, in general, the exact values of the constants are ancillary to this section, which is to show that Tabasco is able to simulate processes such as polymerase interactions and entire genetic systems such as T7. The parameter derivations are detailed are detailed elsewhere [46].

## Availability and Requirements

Project name: TABASCO;

Project home page: ;

Operating system(s): Platform independent;

Programming language: Java;

Other requirements: J2SE 1.4.2 or higher

License: Public domain;

Any restrictions to use by non-academics: n/a.

## Authors' contributions

SK and DE developed the idea of Tabasco. SK did all work on Tabasco design, code development, modeling and simulation, and drafted the manuscript. SK and JRK developed the Tabasco visualization software. SK and DE edited the manuscript. All authors read and approved the final manuscript.

## Supplementary Material

Additional File 1 Visualization of T7 genome simulation using Tabasco. We developed a general visualization tool for depicting graphically and dynamically depicting output from Tabasco. Here, we show output from a Tabasco simulation of three T7 phage simultaneously infecting a single cell over the course of 25 minutes. Each of the three white lines at the top represents a distinct copy of the 39,937 bp T7 genome. The T7 and *E. coli *RNA polymerases are shown as blue and yellow lines moving directly along and above the genomes; directly below the genomes are promoters (dark blue) and terminators (dark red). In addition, the extent of each genomes entry is shown as a solid white line that extends above and below the genome representation (while genome entry is occurring). Protein levels (solid bars) and mRNA levels (white lines) of T7 genes and other host RNA polymerase and ribosome levels are plotted as numbers of molecules per cell on a log-scale below the genome depictions. Each frame is a snapshot of the simulation taken at 5-second intervals; time is displayed in seconds directly below the genome representations. Click here for file

Additional File 2 Input files for simulations described in the text of the paper. Click here for file

Additional File 3 TABASCO website. Click here for file

Additional File 4 Parameter definitions within the input file. The table describes the parameter definitions that are used in the input files that are contained in the supplementary materials. Click here for file
