# Supplementary material for: TABASCO: A single molecule, base-pair resolved gene expression simulator
Source: BMC Bioinformatics. 2007 Dec 19;8:480. doi: 10.1186/1471-2105-8-480 (PMC2242808; doi:10.1186/1471-2105-8-480)
Supplement: Additional File 2 — Input files for simulations described in the text of the paper. [file 1471-2105-8-480-S2.pdf]

Supplementary Information

1. Input Files

Below are input files for simulations described in the text of the paper. These input files may be used as input to the Tabasco simulator (code available at http://openwetware.org/wiki/Tabasco) to recreate the results of the paper.

1.1. Input Files for Simple Gene Expression Models

Tabasco Input File for Single-molecule Simple Gene Expression model


```
<REQUEST>
<EXECUTE-SIMULATION runs="500" random_seed="34143" simulation_name="test-1" time_step="50" time_end="25000"
min_num_for_init_output="1">
<CELL volume="1E-15" growth_rate="0" polymerase_interaction_model="TRAFFIC_JAM">
<RIBOSOME initial_number="10000" speed="40" footprint="35" />
<POLYMERASE n="700" speed="40" id="2" organism_id="2" footprint="15" name="coli Pol" />
<SPECIES name="test" n="0" ID="3" organism="1" />
<REACTIONS>
<REACTION>
3>0;7E-4
</REACTION>
</REACTIONS>
<DNA_SYSTEM name="phage1" genome_length="3800" copy_number="1" entry_rate_constant="50000" entry_offsite="3800">
<PROMOTER start="405" stop="428" startsite="423" name="phi0L" organism_id="1">
        <POLYMERASE polymeraseID="2" aon="4e7" aoff="4" ainiton="1.2" runoff_percent="0" aelong="0.23" arecyc="0" />
</PROMOTER>
<TERMINATOR start="3600" stop="3610" stopsite="3610" name="TE"  organism="2">
<POLYMERASE ID="2" efficiency="0.99" />
</TERMINATOR>
<RBS start="495" stop="500" startsite="500"  initstepsize="20" strength="1.15e4" stopsite="522" protid="3" initRateConstant="0.14"
mrnadeg="2.5e-3" />
</DNA_SYSTEM>
</CELL>
</EXECUTE-SIMULATION>
</REQUEST>
```


Tabasco Input File for Species-level Simple Gene Expression model


```
<REQUEST>
<EXECUTE-SIMULATION runs="1000" random_seed="34143" simulation_name="test-1" time_step="50" time_end="25000"
min_num_for_init_output="1">
<CELL volume="1E-15" growth_rate="0" polymerase_interaction_model="TRAFFIC_JAM">
<RIBOSOME initial_number="10000" speed="40" footprint="35" />
<POLYMERASE n="700" speed="40" id="2" organism_id="2" footprint="15" name="coli Pol" />
<SPECIES name="rib" n="10000" ID="3" organism="1" />
<SPECIES name="pol" n="700" ID="4" organism="1" />
<SPECIES name="promoter" n="1" ID="5" organism="1" />
<SPECIES name="pol-promoter" n="0" ID="6" organism="1" />
<SPECIES name="pol-promoter-init" n="0" ID="7" organism="1" />
<SPECIES name="pol-dna-elong" n="0" ID="8" organism="1" />
<SPECIES name="rbs" n="0" ID="9" organism="1" />
<SPECIES name="rib-rbs" n="0" ID="10" organism="1" />
```

<SPECIES name="rib-elong" n="0" ID="11" organism="1" />
<SPECIES name="cust_prot" n="0" ID="12" organism="1" />
<REACTIONS>
<REACTION>
4+5>6;4e7
</REACTION>
<REACTION>
6>4+5;4
</REACTION>
<REACTION>
6>7;1.2
</REACTION>
<REACTION>
7>5+8;0.23
</REACTION>
<REACTION>
8>4+9;0.645
</REACTION>
<REACTION>
3+9>10;1.15e4
</REACTION>
<REACTION>
10>9+11;0.1308
</REACTION>
<REACTION>
11>3+12;20
</REACTION>
<REACTION>
12>0;7e-4
</REACTION>
<REACTION>
9>0;2.5e-3
</REACTION>
<REACTION>
10>3;2.5e-3
</REACTION>
</REACTIONS>
<DNA_SYSTEM name="phage1" genome_length="3800" copy_number="1" entry_rate_constant="50" entry_offsite="850">
<PROMOTER start="405" stop="428" startsite="423" name="phi0L" organism_id="1">
<POLYMERASE polymeraseID="2" aon="0" aoff="4" ainiton="1.2" runoff_percent="35" aelong="5" arecyc="0.7" />
</PROMOTER>
<TERMINATOR start="3600" stop="3610" stopsite="3610" name="TE" organism="2">
<POLYMERASE ID="2" efficiency="0.99" />
</TERMINATOR>
<RBS start="485" stop="500" startsite="500" initstepsize="50" strength="0" stopsite="3500" protid="3" initRateConstant="0.14" mrnadeg="2.5e-3" />
</DNA_SYSTEM>
</CELL>
</EXECUTE-SIMULATION>
</REQUEST>

1.2. Input Files for Polymerase Interaction Simulations

Tabasco Input File for Downstream Falloff

<REQUEST>
<EXECUTE-SIMULATION runs="100" random_seed="34206" simulation_name="test-1" time_step="50" time_end="25000" min_num_for_init_output="1">
<CELL volume="1E-15" growth_rate="0" polymerase_interaction_model="DOWNSTREAM_FALL_OFF">
<RIBOSOME initial_number="10000" speed="40" footprint="35" />
<POLYMERASE n="700" speed="300" id="2" organism_id="1" footprint="15" name="T7 pol" />
<POLYMERASE n="700" speed="40" id="3" organism_id="2" footprint="15" name="Coli pol" />
<SPECIES name="test-1" n="0" ID="4" organism="1" />
<SPECIES name="test-2" n="0" ID="5" organism="1" />
<REACTIONS>
<REACTION>

4>0;7E-4
</REACTION>
<REACTION>
5>0;7E-4
</REACTION>
</REACTIONS>
<DNA_SYSTEM name="phage1" genome_length="4000" copy_number="1" entry_rate_constant="5000" entry_offsite="3950">
<PROMOTER start="405" stop="428" startsite="423" name="A1" organism_id="2">
<POLYMERASE polymeraseID="3" aon="1.5e7" aoff="0.28" ainiton="0.2" runoff_percent="100" aelong="6" arecyc="0" />
</PROMOTER>
<PROMOTER start="1505" stop="1528" startsite="1523" name="phi10" organism_id="1">
<POLYMERASE polymeraseID="2" aon="1.26e8" aoff="0.2" ainiton="1.2" runoff_percent="70" aelong="5" arecyc="0.7" />
</PROMOTER>
<TERMINATOR start="3900" stop="3910" stopsite="3910" name="TE" organism="2">
<POLYMERASE ID="2" efficiency="0.99" />
<POLYMERASE ID="3" efficiency="0.99" />
</TERMINATOR>
<RBS start="485" stop="500" startsite="500" initstepsize="50" strength="1.15e4" stopsite="1499" protid="4"
initRateConstant="0.14" mrnadeg="2.5e-3" />
<RBS start="1585" stop="1600" startsite="1600" initstepsize="50" strength="1.15e4" stopsite="3600" protid="5"
initRateConstant="0.14" mrnadeg="2.5e-3" />
</DNA_SYSTEM>
</CELL>
</EXECUTE-SIMULATION>
</REQUEST>

Tabasco Input File for Upstream Falloff

<REQUEST>
<EXECUTE-SIMULATION runs="100" random_seed="34206" simulation_name="test-1" time_step="50" time_end="25000"
min_num_for_init_output="1">
<CELL volume="1E-15" growth_rate="0" polymerase_interaction_model="UPSTREAM_FALL_OFF">
<RIBOSOME initial_number="10000" speed="40" footprint="35" />
<POLYMERASE n="700" speed="300" id="2" organism_id="1" footprint="15" name="T7 pol" />
<POLYMERASE n="700" speed="40" id="3" organism_id="2" footprint="15" name="Coli pol" />
<SPECIES name="test-1" n="0" ID="4" organism="1" />
<SPECIES name="test-2" n="0" ID="5" organism="1" />
<REACTIONS>
<REACTION>
4>0;7E-4
</REACTION>
<REACTION>
5>0;7E-4
</REACTION>
</REACTIONS>
<DNA_SYSTEM name="phage1" genome_length="4000" copy_number="1" entry_rate_constant="5000" entry_offsite="3950">
<PROMOTER start="405" stop="428" startsite="423" name="A1" organism_id="2">
<POLYMERASE polymeraseID="3" aon="1.5e7" aoff="0.28" ainiton="0.2" runoff_percent="100" aelong="6" arecyc="0" />
</PROMOTER>
<PROMOTER start="1505" stop="1528" startsite="1523" name="phi10" organism_id="1">
<POLYMERASE polymeraseID="2" aon="1.26e8" aoff="0.2" ainiton="1.2" runoff_percent="70" aelong="5" arecyc="0.7" />
</PROMOTER>
<TERMINATOR start="3900" stop="3910" stopsite="3910" name="TE" organism="2">
<POLYMERASE ID="2" efficiency="0.99" />
<POLYMERASE ID="3" efficiency="0.99" />
</TERMINATOR>
<RBS start="485" stop="500" startsite="500" initstepsize="50" strength="1.15e4" stopsite="1499" protid="4"
initRateConstant="0.14" mrnadeg="2.5e-3" />
<RBS start="1585" stop="1600" startsite="1600" initstepsize="50" strength="1.15e4" stopsite="3600" protid="5"
initRateConstant="0.14" mrnadeg="2.5e-3" />
</DNA_SYSTEM>
</CELL>
</EXECUTE-SIMULATION>
</REQUEST>

Tabasco Input File for Traffic Jam


```
<REQUEST>
<EXECUTE-SIMULATION runs="100" random_seed="34206" simulation_name="test-1" time_step="50" time_end="25000"
min_num_for_init_output="1">
<CELL volume="1E-15" growth_rate="0" polymerase_interaction_model="TRAFFIC_JAM">
<RIBOSOME initial_number="10000" speed="40" footprint="35" />
<POLYMERASE n="700" speed="300" id="2" organism_id="1" footprint="15" name="T7 pol" />
<POLYMERASE n="700" speed="40" id="3" organism_id="2" footprint="15" name="Coli pol" />
<SPECIES name="test-1" n="0" ID="4" organism="1" />
<SPECIES name="test-2" n="0" ID="5" organism="1" />
<REACTIONS>
<REACTION>
4>0;7E-4
</REACTION>
<REACTION>
5>0;7E-4
</REACTION>
</REACTIONS>
<DNA_SYSTEM name="phage1" genome_length="4000" copy_number="1" entry_rate_constant="5000" entry_offsite="3950">
<PROMOTER start="405" stop="428" startsite="423" name="A1" organism_id="2">
        <POLYMERASE polymeraseID="3" aon="1.5e7" aoff="0.28" ainiton="0.2" runoff_percent="100" aelong="6" arecyc="0" />
</PROMOTER>
<PROMOTER start="1505" stop="1528" startsite="1523" name="phi10" organism_id="1">
        <POLYMERASE polymeraseID="2" aon="1.26e8" aoff="0.2" ainiton="1.2" runoff_percent="70" aelong="5" arecyc="0.7" />
</PROMOTER>
<TERMINATOR start="3900" stop="3910" stopsite="3910" name="TE"  organism="2">
<POLYMERASE ID="2" efficiency="0.99" />
<POLYMERASE ID="3" efficiency="0.99" />
</TERMINATOR>
<RBS start="485" stop="500" startsite="500"  initstepsize="50" strength="1.15e4" stopsite="1499" protid="4"
initRateConstant="0.14" mrnadeg="2.5e-3" />
<RBS start="1585" stop="1600" startsite="1600"  initstepsize="50" strength="1.15e4" stopsite="3600" protid="5"
initRateConstant="0.14" mrnadeg="2.5e-3" />
</DNA_SYSTEM>
</CELL>
</EXECUTE-SIMULATION>
</REQUEST>
```


Tabasco Input File for Traffic Jam with No T7 RNA Polymerase


```
<REQUEST>
<EXECUTE-SIMULATION runs="100" random_seed="34206" simulation_name="test-1" time_step="50" time_end="25000"
min_num_for_init_output="1">
<CELL volume="1E-15" growth_rate="0" polymerase_interaction_model="TRAFFIC_JAM">
<RIBOSOME initial_number="10000" speed="40" footprint="35" />
<POLYMERASE n="0" speed="300" id="2" organism_id="1" footprint="15" name="T7 pol" />
<POLYMERASE n="700" speed="40" id="3" organism_id="2" footprint="15" name="Coli pol" />
<SPECIES name="test-1" n="0" ID="4" organism="1" />
<SPECIES name="test-2" n="0" ID="5" organism="1" />
<REACTIONS>
<REACTION>
4>0;7E-4
</REACTION>
<REACTION>
5>0;7E-4
</REACTION>
</REACTIONS>
<DNA_SYSTEM name="phage1" genome_length="4000" copy_number="1" entry_rate_constant="5000" entry_offsite="3950">
<PROMOTER start="405" stop="428" startsite="423" name="A1" organism_id="2">
        <POLYMERASE polymeraseID="3" aon="1.5e7" aoff="0.28" ainiton="0.2" runoff_percent="100" aelong="6" arecyc="0" />
</PROMOTER>
```

```
<PROMOTER start="1505" stop="1528" startsite="1523" name="phi10" organism_id="1">
	<POLYMERASE polymeraseID="2" aon="1.26e8" aoff="0.2" ainiton="1.2" runoff_percent="70" aelong="5" arecyc="0.7" />
</PROMOTER>
<TERMINATOR start="3900" stop="3910" stopsite="3910" name="TE"  organism="2">
<POLYMERASE ID="2" efficiency="0.99" />
<POLYMERASE ID="3" efficiency="0.99" />
</TERMINATOR>
<RBS start="485" stop="500" startsite="500"  initstepsize="50" strength="1.15e4" stopsite="1499" protid="4"
initRateConstant="0.14" mrnadeg="2.5e-3" />
<RBS start="1585" stop="1600" startsite="1600"  initstepsize="50" strength="1.15e4" stopsite="3600" protid="5"
initRateConstant="0.14" mrnadeg="2.5e-3" />
</DNA_SYSTEM>
</CELL>
</EXECUTE-SIMULATION>
</REQUEST>
```


Tabasco Input File for Traffic Jam with No E. coli RNA polymerase


```
<REQUEST>
<EXECUTE-SIMULATION runs="100" random_seed="34206" simulation_name="test-1" time_step="50" time_end="25000"
min_num_for_init_output="1">
<CELL volume="1E-15" growth_rate="0" polymerase_interaction_model="TRAFFIC_JAM">
<RIBOSOME initial_number="10000" speed="40" footprint="35" />
<POLYMERASE n="700" speed="300" id="2" organism_id="1" footprint="15" name="T7 pol" />
<POLYMERASE n="0" speed="40" id="3" organism_id="2" footprint="15" name="Coli pol" />
<SPECIES name="test-1" n="0" ID="4" organism="1" />
<SPECIES name="test-2" n="0" ID="5" organism="1" />
<REACTIONS>
<REACTION>
4>0;7E-4
</REACTION>
<REACTION>
5>0;7E-4
</REACTION>
</REACTIONS>
<DNA_SYSTEM name="phage1" genome_length="4000" copy_number="1" entry_rate_constant="5000" entry_offsite="3950">
<PROMOTER start="405" stop="428" startsite="423" name="A1" organism_id="2">
	<POLYMERASE polymeraseID="3" aon="1.5e7" aoff="0.28" ainiton="0.2" runoff_percent="100" aelong="6" arecyc="0" />
</PROMOTER>
<PROMOTER start="1505" stop="1528" startsite="1523" name="phi10" organism_id="1">
	<POLYMERASE polymeraseID="2" aon="1.26e8" aoff="0.2" ainiton="1.2" runoff_percent="70" aelong="5" arecyc="0.7" />
</PROMOTER>
<TERMINATOR start="3900" stop="3910" stopsite="3910" name="TE"  organism="2">
<POLYMERASE ID="2" efficiency="0.99" />
<POLYMERASE ID="3" efficiency="0.99" />
</TERMINATOR>
<RBS start="485" stop="500" startsite="500"  initstepsize="50" strength="1.15e4" stopsite="1499" protid="4"
initRateConstant="0.14" mrnadeg="2.5e-3" />
<RBS start="1585" stop="1600" startsite="1600"  initstepsize="50" strength="1.15e4" stopsite="3600" protid="5"
initRateConstant="0.14" mrnadeg="2.5e-3" />
</DNA_SYSTEM>
</CELL>
</EXECUTE-SIMULATION>
</REQUEST>
```


1.3. Input Files for T7 Genome Simulation

Tabasco Input File for T7 Gene Expression Simulation:


```
<REQUEST>
```

<EXECUTE-SIMULATION runs="1" random_seed="34145" simulation_name="test-1" time_step="5" time_end="1500" write_D-
NA="no" write_InitRNA="yes" write_Energy="yes" compDeg="yes">
<CELL volume="8e-16" polymerase_interaction_model="TRAFFIC_JAM">
<RIBOSOME initial_number="10000" speed="45" footprint="40" />
<POLYMERASE n="1800" speed="45" id="2" organism_id="2" footprint="15" name="coli Pol" />
<POLYMERASE n="0" speed="45" id="3" organism_id="2" footprint="15" name="coli Pol-P" />
<POLYMERASE n="0" speed="45" id="4" organism_id="2" footprint="15" name="coli Pol-2.0" />
<POLYMERASE n="0" speed="45" id="5" organism_id="2" footprint="15" name="coli Pol-P-2.0" />
<POLYMERASE n="0" speed="230" id="6" organism_id="1" footprint="15" name="gp1" />
<POLYMERASE n="0" speed="230" id="7" organism_id="1" footprint="15" name="gp1-3.5" />
<SPECIES name="gp0.3" n="0" ID="8" organism="1" />
<SPECIES name="gp0.4" n="0" ID="9" organism="1" />
<SPECIES name="gp0.5" n="0" ID="10" organism="1" />
<SPECIES name="gp0.6A" n="0" ID="11" organism="1" />
<SPECIES name="gp0.7" n="0" ID="12" organism="1" />
<SPECIES name="gp1.1" n="0" ID="13" organism="1" />
<SPECIES name="gp1.2" n="0" ID="14" organism="1" />
<SPECIES name="gp1.3" n="0" ID="15" organism="1" />
<SPECIES name="gp1.4" n="0" ID="16" organism="1" />
<SPECIES name="gp1.5" n="0" ID="17" organism="1" />
<SPECIES name="gp1.6" n="0" ID="18" organism="1" />
<SPECIES name="gp1.7" n="0" ID="19" organism="1" />
<SPECIES name="gp1.8" n="0" ID="20" organism="1" />
<SPECIES name="gp2.0" n="0" ID="21" organism="1" />
<SPECIES name="gp2.5" n="0" ID="22" organism="1" />
<SPECIES name="gp2.8" n="0" ID="23" organism="1" />
<SPECIES name="gp3.0" n="0" ID="24" organism="1" />
<SPECIES name="gp3.5" n="0" ID="25" organism="1" />
<SPECIES name="gp3.8" n="0" ID="26" organism="1" />
<SPECIES name="gp4A" n="0" ID="27" organism="1" />
<SPECIES name="gp4.2" n="0" ID="28" organism="1" />
<SPECIES name="gp4.3" n="0" ID="29" organism="1" />
<SPECIES name="gp4.5" n="0" ID="30" organism="1" />
<SPECIES name="gp4.7" n="0" ID="31" organism="1" />
<SPECIES name="gp5.0" n="0" ID="32" organism="1" />
<SPECIES name="gp5.3" n="0" ID="33" organism="1" />
<SPECIES name="gp5.5" n="0" ID="34" organism="1" />
<SPECIES name="gp5.7" n="0" ID="35" organism="1" />
<SPECIES name="gp5.9" n="0" ID="36" organism="1" />
<SPECIES name="gp6.0" n="0" ID="37" organism="1" />
<SPECIES name="gp6.3" n="0" ID="38" organism="1" />
<SPECIES name="gp6.5" n="0" ID="39" organism="1" />
<SPECIES name="gp6.7" n="0" ID="40" organism="1" />
<SPECIES name="gp7" n="0" ID="41" organism="1" />
<SPECIES name="gp7.3" n="0" ID="42" organism="1" />
<SPECIES name="gp7.7" n="0" ID="43" organism="1" />
<SPECIES name="gp8" n="0" ID="44" organism="1" />
<SPECIES name="gp9" n="0" ID="45" organism="1" />
<SPECIES name="gp10A" n="0" ID="46" organism="1" />
<SPECIES name="gp11" n="0" ID="47" organism="1" />
<SPECIES name="gp12" n="0" ID="48" organism="1" />
<SPECIES name="gp13" n="0" ID="49" organism="1" />
<SPECIES name="gp14" n="0" ID="50" organism="1" />
<SPECIES name="gp15" n="0" ID="51" organism="1" />
<SPECIES name="gp16" n="0" ID="52" organism="1" />
<SPECIES name="gp17" n="0" ID="53" organism="1" />
<SPECIES name="gp17.5" n="0" ID="54" organism="1" />
<SPECIES name="gp18" n="0" ID="55" organism="1" />
<SPECIES name="gp18.5" n="0" ID="56" organism="1" />
<SPECIES name="gp19" n="0" ID="57" organism="1" />
<SPECIES name="gp19.5" n="0" ID="58" organism="1" />
<REACTIONS>
<REACTION>
12+2>3+11;3.8E7
</REACTION>
<REACTION>
12+4>5+11;3.8E7
</REACTION>
<REACTION>
21+2>4;3.8E7
</REACTION>
<REACTION>

21+3>5;3.8E7
</REACTION>
<REACTION>
4>21+2;1.1
</REACTION>
<REACTION>
5>21+3;1.1
</REACTION>
<REACTION>
25+6>7;3.8E7
</REACTION>
<REACTION>
7>6+25;3.5
</REACTION>
</REACTIONS>
<DNA_SYSTEM name="phage1" genome_length="39937" copy_number="3" entry_rate_constant="70" entry_offsite="850">
<PROMOTER start="405" stop="428" startsite="423" name="phi0L" organism_id="1">
<POLYMERASE polymeraseID="6" aon="1.82e8" aoff="0.2" ainiton="3.5" runoff_percent="50" aelong="5.8"
arecyc="0.88" />
<POLYMERASE polymeraseID="7" aon="1.82e8" aoff="0.2" ainiton="0.875" runoff_percent="18" aelong="1.45"
arecyc="0.22" />
</PROMOTER>
<PROMOTER start="498" stop="548" startsite="542" name="A1" organism_id="2">
<POLYMERASE polymeraseID="2" aon="1.5e7" aoff=".28" ainiton="0.4" runoff_percent="100" aelong="6" arecyc="0"/>
<POLYMERASE polymeraseID="3" aon="1.5e7" aoff=".28" ainiton="0.4" runoff_percent="70" aelong="6" arecyc="0.19"/>
<POLYMERASE polymeraseID="4" aon="0" aoff="0" ainiton="0" runoff_percent="0" aelong="0" arecyc="0"/>
<POLYMERASE polymeraseID="5" aon="0" aoff="0" ainiton="0" runoff_percent="0" aelong="0" arecyc="0"/>
</PROMOTER>
<PROMOTER start="626" stop="676" startsite="670" name="A2" organism_id="2">
<POLYMERASE polymeraseID="2" aon="1.5e7" aoff=".28" ainiton="0.4" runoff_percent="100" aelong="6" arecyc="0"/>
<POLYMERASE polymeraseID="3" aon="1.5e7" aoff=".28" ainiton="0.4" runoff_percent="70" aelong="6" arecyc="0.19"/>
<POLYMERASE polymeraseID="4" aon="0" aoff="0" ainiton="0" runoff_percent="0" aelong="0" arecyc="0"/>
<POLYMERASE polymeraseID="5" aon="0" aoff="0" ainiton="0" runoff_percent="0" aelong="0" arecyc="0"/>
</PROMOTER>
<PROMOTER start="750" stop="800" startsite="794" name="A3" organism_id="2">
<POLYMERASE polymeraseID="2" aon="1.5e7" aoff=".28" ainiton="0.4" runoff_percent="100" aelong="6" arecyc="0"/>
<POLYMERASE polymeraseID="3" aon="1.5e7" aoff=".28" ainiton="0.4" runoff_percent="70" aelong="6" arecyc="0.19"/>
<POLYMERASE polymeraseID="4" aon="0" aoff="0" ainiton="0" runoff_percent="0" aelong="0" arecyc="0"/>
<POLYMERASE polymeraseID="5" aon="0" aoff="0" ainiton="0" runoff_percent="0" aelong="0" arecyc="0"/>
</PROMOTER>
<PROMOTER start="1514" stop="1564" startsite="1558" name="B" organism_id="2">
<POLYMERASE polymeraseID="2" aon="1.5e7" aoff="2.8" ainiton="0.19" runoff_percent="100" aelong="6" arecyc="0"/>
<POLYMERASE polymeraseID="3" aon="1.5e7" aoff="2.8" ainiton="0.19" runoff_percent="70" aelong="6"
arecyc="0.19"/>
<POLYMERASE polymeraseID="4" aon="0" aoff="0" ainiton="0" runoff_percent="0" aelong="0" arecyc="0"/>
<POLYMERASE polymeraseID="5" aon="0" aoff="0" ainiton="0" runoff_percent="0" aelong="0" arecyc="0"/>
</PROMOTER>
<PROMOTER start="3113" stop="3163" startsite="3157" name="C" organism_id="2">
<POLYMERASE polymeraseID="2" aon="1.5e7" aoff="2.8" ainiton="0.19" runoff_percent="100" aelong="6" arecyc="0"/>
<POLYMERASE polymeraseID="3" aon="1.5e7" aoff="2.8" ainiton="0.19" runoff_percent="70" aelong="6"
arecyc="0.19"/>
<POLYMERASE polymeraseID="4" aon="0" aoff="0" ainiton="0" runoff_percent="0" aelong="0" arecyc="0"/>
<POLYMERASE polymeraseID="5" aon="0" aoff="0" ainiton="0" runoff_percent="0" aelong="0" arecyc="0"/>
</PROMOTER>
<PROMOTER start="5848" stop="5871" startsite="5866" name="phi1.1A" organism_id="1">
<POLYMERASE polymeraseID="6" aon="6E7" aoff="18" ainiton="3.5" runoff_percent="50" aelong="5.8" arecyc="0.88" />
<POLYMERASE polymeraseID="7" aon="6e7" aoff="18" ainiton="0.875" runoff_percent="18" aelong="1.45"
arecyc="0.22" />
</PROMOTER>
<PROMOTER start="5923" stop="5946" startsite="5941" name="phi1.1B" organism_id="1">
<POLYMERASE polymeraseID="6" aon="1.82e8" aoff="0.2" ainiton="3.5" runoff_percent="50" aelong="5.8"
arecyc="0.88" />
<POLYMERASE polymeraseID="7" aon="1.82e8" aoff="0.2" ainiton="0.875" runoff_percent="18" aelong="1.45"
arecyc="0.22" />
</PROMOTER>
<PROMOTER start="6409" stop="6432" startsite="6427" name="phi1.3" organism_id="1">
<POLYMERASE polymeraseID="6" aon="6e7" aoff="18" ainiton="3.5" runoff_percent="50" aelong="5.8" arecyc="0.88" />
<POLYMERASE polymeraseID="7" aon="6e7" aoff="18" ainiton="0.875" runoff_percent="18" aelong="1.45"
arecyc="0.22" />
</PROMOTER>
<PROMOTER start="7778" stop="7801" startsite="7796" name="phi1.5" organism_id="1">

<POLYMERASE polymeraseID="6" aon="1.82e8" aoff="0.2" ainiton="3.5" runoff_percent="50" aelong="5.8" arecyc="0.88" />
<POLYMERASE polymeraseID="7" aon="1.82e8" aoff="0.2" ainiton="0.875" runoff_percent="18" aelong="1.45" arecyc="0.22" />
</PROMOTER>
<PROMOTER start="7895" stop="7918" startsite="7913" name="phi1.6" organism_id="1">
<POLYMERASE polymeraseID="6" aon="1.82e8" aoff="0.2" ainiton="3.5" runoff_percent="50" aelong="5.8" arecyc="0.88" />
<POLYMERASE polymeraseID="7" aon="1.82e8" aoff="0.2" ainiton="0.875" runoff_percent="18" aelong="1.45" arecyc="0.22" />
</PROMOTER>
<PROMOTER start="9107" stop="9130" startsite="9125" name="phi2.5" organism_id="1">
<POLYMERASE polymeraseID="6" aon="1.82e8" aoff="0.2" ainiton="3.5" runoff_percent="50" aelong="5.8" arecyc="0.88" />
<POLYMERASE polymeraseID="7" aon="1.82e8" aoff="0.2" ainiton="0.875" runoff_percent="18" aelong="1.45" arecyc="0.22" />
</PROMOTER>
<PROMOTER start="11180" stop="11203" startsite="11198" name="phi3.8" organism_id="1">
<POLYMERASE polymeraseID="6" aon="6e7" aoff="18" ainiton="3.5" runoff_percent="50" aelong="5.8" arecyc="0.88" />
<POLYMERASE polymeraseID="7" aon="6e7" aoff="18" ainiton="0.875" runoff_percent="18" aelong="1.45" arecyc="0.22" />
</PROMOTER>
<PROMOTER start="12671" stop="12694" startsite="12689" name="phi4c" organism_id="1">
<POLYMERASE polymeraseID="6" aon="6e7" aoff="18" ainiton="3.5" runoff_percent="50" aelong="5.8" arecyc="0.88" />
<POLYMERASE polymeraseID="7" aon="6e7" aoff="18" ainiton="0.875" runoff_percent="18" aelong="1.45" arecyc="0.22" />
</PROMOTER>
<PROMOTER start="13341" stop="13364" startsite="13359" name="phi4.3" organism_id="1">
<POLYMERASE polymeraseID="6" aon="1.82e8" aoff="0.2" ainiton="3.5" runoff_percent="50" aelong="5.8" arecyc="0.88" />
<POLYMERASE polymeraseID="7" aon="1.82e8" aoff="0.2" ainiton="0.875" runoff_percent="18" aelong="1.45" arecyc="0.22" />
</PROMOTER>
<PROMOTER start="13915" stop="13938" startsite="13933" name="phi4.7" organism_id="1">
<POLYMERASE polymeraseID="6" aon="6e7" aoff="18" ainiton="3.5" runoff_percent="50" aelong="5.8" arecyc="0.88" />
<POLYMERASE polymeraseID="7" aon="6e7" aoff="18" ainiton="0.875" runoff_percent="18" aelong="1.45" arecyc="0.22" />
</PROMOTER>
<PROMOTER start="18545" stop="18568" startsite="18563" name="phi6.5" organism_id="1">
<POLYMERASE polymeraseID="6" aon="1.82e8" aoff="0.2" ainiton="3.5" runoff_percent="100" aelong="5.8" arecyc="0.88" />
<POLYMERASE polymeraseID="7" aon="1.82e8" aoff="0.2" ainiton="0.875" runoff_percent="42" aelong="1.45" arecyc="0.22" />
</PROMOTER>
<PROMOTER start="21865" stop="21888" startsite="21883" name="phi9" organism_id="1">
<POLYMERASE polymeraseID="6" aon="1.82e8" aoff="0.2" ainiton="3.5" runoff_percent="100" aelong="5.8" arecyc="0.88" />
<POLYMERASE polymeraseID="7" aon="1.82e8" aoff="0.2" ainiton="0.875" runoff_percent="42" aelong="1.45" arecyc="0.22" />
</PROMOTER>
<PROMOTER start="22904" stop="22927" startsite="22922" name="phi10" organism_id="1">
<POLYMERASE polymeraseID="6" aon="1.82e8" aoff="0.2" ainiton="3.5" runoff_percent="100" aelong="5.8" arecyc="0.88" />
<POLYMERASE polymeraseID="7" aon="1.82e8" aoff="0.2" ainiton="0.875" runoff_percent="42" aelong="1.45" arecyc="0.22" />
</PROMOTER>
<PROMOTER start="27274" stop="27297" startsite="27292" name="phi13" organism_id="1">
<POLYMERASE polymeraseID="6" aon="1.82e8" aoff="0.2" ainiton="3.5" runoff_percent="100" aelong="5.8" arecyc="0.88" />
<POLYMERASE polymeraseID="7" aon="1.82e8" aoff="0.2" ainiton="0.875" runoff_percent="42" aelong="1.45" arecyc="0.22" />
</PROMOTER>
<PROMOTER start="34566" stop="34589" startsite="34584" name="phi17" organism_id="1">
<POLYMERASE polymeraseID="6" aon="1.82e8" aoff="0.2" ainiton="3.5" runoff_percent="100" aelong="5.8" arecyc="0.88" />
<POLYMERASE polymeraseID="7" aon="1.82e8" aoff="0.2" ainiton="0.875" runoff_percent="42" aelong="1.45" arecyc="0.22" />
</PROMOTER>
<PROMOTER start="39229" stop="39252" startsite="39247" name="phiOR" organism_id="1">
<POLYMERASE polymeraseID="6" aon="1.82e8" aoff="0.2" ainiton="3.5" runoff_percent="100" aelong="5.8" arecyc="0.88" />

<POLYMERASE polymeraseID="7" aon="1.82e8" aoff="0.2" ainiton="0.875" runoff_percent="42" aelong="1.45" arecyc="0.22" />
</PROMOTER>
<TERMINATOR start="7546" stop="7610" stopsite="7588" name="TE" organism="2">
<POLYMERASE ID="2" efficiency="1" />
<POLYMERASE ID="3" efficiency="1" />
<POLYMERASE ID="4" efficiency="1" />
<POLYMERASE ID="5" efficiency="1" />
<POLYMERASE ID="6" efficiency="0" />
<POLYMERASE ID="7" efficiency="0" />
</TERMINATOR>
<TERMINATOR start="24158" stop="24230" stopsite="24209" name="Tphi" organism="1">
<POLYMERASE ID="2" efficiency="0" />
<POLYMERASE ID="3" efficiency="0" />
<POLYMERASE ID="4" efficiency="0" />
<POLYMERASE ID="5" efficiency="0" />
<POLYMERASE ID="6" efficiency="0.8" />
<POLYMERASE ID="7" efficiency="0.8" />
</TERMINATOR>
<RBS start="910" stop="925" startsite="925" initstepsize="50" elongstepsize="" strength="1e7" stopsite="1278" protid="8" initRateConstant="0.3" mrnadeg="8.33e-3" />
<RBS start="1262" stop="1277" startsite="1277" initstepsize="50" elongstepsize="" strength="1e7" stopsite="1433" protid="9" initRateConstant="0.3" mrnadeg="8.33e-3" />
<RBS start="1454" stop="1469" startsite="1469" initstepsize="50" elongstepsize="" strength="1e7" stopsite="1639" protid="10" initRateConstant="0.3" mrnadeg="8.33e-3" />
<RBS start="1621" stop="1636" startsite="1636" initstepsize="50" elongstepsize="" strength="1e7" stopsite="1797" protid="11" initRateConstant="0.3" mrnadeg="8.33e-3" />
<RBS start="2006" stop="2021" startsite="2021" initstepsize="50" elongstepsize="" strength="1e7" stopsite="3100" protid="12" initRateConstant="0.3" mrnadeg="1.67e-2" />
<RBS start="3156" stop="3171" startsite="3171" initstepsize="50" elongstepsize="" strength="1e7" stopsite="5822" protid="6" initRateConstant="0.3" mrnadeg="1.67e-2" />
<RBS start="5992" stop="6007" startsite="6007" initstepsize="50" elongstepsize="" strength="1e7" stopsite="6135" protid="13" initRateConstant="0.3" mrnadeg="3.33e-3" />
<RBS start="6119" stop="6134" startsite="6134" initstepsize="50" elongstepsize="" strength="1e7" stopsite="6394" protid="14" initRateConstant="0.3" mrnadeg="8.33e-3" />
<RBS start="6460" stop="6475" startsite="6475" initstepsize="50" elongstepsize="" strength="1e7" stopsite="7554" protid="15" initRateConstant="0.3" mrnadeg="1.67e-2" />
<RBS start="7593" stop="7608" startsite="7608" initstepsize="50" elongstepsize="" strength="1e7" stopsite="7763" protid="16" initRateConstant="0.3" mrnadeg="8.33e-3" />
<RBS start="7776" stop="7791" startsite="7791" initstepsize="50" elongstepsize="" strength="1e7" stopsite="7880" protid="17" initRateConstant="0.3" mrnadeg="5.56e-3" />
<RBS start="7891" stop="7906" startsite="7906" initstepsize="50" elongstepsize="" strength="1e7" stopsite="8166" protid="18" initRateConstant="0.3" mrnadeg="5.56e-3" />
<RBS start="8150" stop="8165" startsite="8165" initstepsize="50" elongstepsize="" strength="1e7" stopsite="8756" protid="19" initRateConstant="0.3" mrnadeg="3.33e-3" />
<RBS start="8734" stop="8749" startsite="8749" initstepsize="50" elongstepsize="" strength="1e7" stopsite="8895" protid="20" initRateConstant="0.3" mrnadeg="5.56e-3" />
<RBS start="8879" stop="8894" startsite="8894" initstepsize="50" elongstepsize="" strength="1e7" stopsite="9092" protid="21" initRateConstant="0.3" mrnadeg="0" />
<RBS start="9136" stop="9152" startsite="9158" initstepsize="50" elongstepsize="" strength="1e7" stopsite="9856" protid="22" initRateConstant="0.3" mrnadeg="2.78e-3" />
<RBS start="9840" stop="9855" startsite="9855" initstepsize="50" elongstepsize="" strength="1e7" stopsite="10276" protid="23" initRateConstant="0.3" mrnadeg="5.56e-3" />
<RBS start="10242" stop="10257" startsite="10257" initstepsize="50" elongstepsize="" strength="1e7" stopsite="10706" protid="24" initRateConstant="0.3" mrnadeg="5.56e-3" />
<RBS start="10690" stop="10705" startsite="10705" initstepsize="50" elongstepsize="" strength="1e7" stopsite="11161" protid="25" initRateConstant="0.3" mrnadeg="1.11e-3" />
<RBS start="11210" stop="11225" startsite="11225" initstepsize="50" elongstepsize="" strength="1e7" stopsite="11590" protid="26" initRateConstant="0.3" mrnadeg="5.56e-3" />
<RBS start="11550" stop="11565" startsite="11565" initstepsize="50" elongstepsize="" strength="1e7" stopsite="13265" protid="27" initRateConstant="0.3" mrnadeg="5.56e-3" />
<RBS start="12973" stop="12988" startsite="12988" initstepsize="50" elongstepsize="" strength="1e7" stopsite="13326" protid="28" initRateConstant="0.3" mrnadeg="5.56e-3" />
<RBS start="13337" stop="13352" startsite="13352" initstepsize="50" elongstepsize="" strength="1e7" stopsite="13564" protid="29" initRateConstant="0.3" mrnadeg="5.56e-3" />
<RBS start="13575" stop="13584" startsite="13584" initstepsize="50" elongstepsize="" strength="1e7" stopsite="13853" protid="30" initRateConstant="0.3" mrnadeg="5.56e-3" />
<RBS start="13912" stop="13927" startsite="13927" initstepsize="50" elongstepsize="" strength="1e7" stopsite="14334" protid="31" initRateConstant="0.3" mrnadeg="5.56e-3" />
<RBS start="14338" stop="14353" startsite="14353" initstepsize="50" elongstepsize="" strength="1e7" stopsite="16467" protid="32" initRateConstant="0.3" mrnadeg="2.78e-3" />

<RBS start="16468" stop="16483" startsite="16483" initstepsize="50" elongstepsize="" strength="1e7" stopsite="16838" protid="33" initRateConstant="0.3" mrnadeg="5.56e-3" />
<RBS start="16839" stop="16851" startsite="16851" initstepsize="50" elongstepsize="" strength="1e7" stopsite="17159" protid="34" initRateConstant="0.3" mrnadeg="5.56e-3" />
<RBS start="17124" stop="17149" startsite="17149" initstepsize="50" elongstepsize="" strength="1e7" stopsite="17359" protid="35" initRateConstant="0.3" mrnadeg="5.56e-3" />
<RBS start="17343" stop="17358" startsite="17358" initstepsize="50" elongstepsize="" strength="1e7" stopsite="17517" protid="36" initRateConstant="0.3" mrnadeg="5.56e-3" />
<RBS start="17489" stop="17504" startsite="17504" initstepsize="50" elongstepsize="" strength="1e7" stopsite="18406" protid="37" initRateConstant="0.3" mrnadeg="4.17e-3" />
<RBS start="18379" stop="18394" startsite="18394" initstepsize="50" elongstepsize="" strength="1e7" stopsite="18507" protid="38" initRateConstant="0.3" mrnadeg="5.56e-3" />
<RBS start="18590" stop="18605" startsite="18605" initstepsize="50" elongstepsize="" strength="1e7" stopsite="18859" protid="39" initRateConstant="0.3" mrnadeg="5.56e-3" />
<RBS start="18860" stop="18864" startsite="18864" initstepsize="50" elongstepsize="" strength="1e7" stopsite="19131" protid="40" initRateConstant="0.3" mrnadeg="8.33e-3" />
<RBS start="19114" stop="19129" startsite="19130" initstepsize="50" elongstepsize="" strength="1e7" stopsite="19531" protid="41" initRateConstant="0.3" mrnadeg="8.33e-3" />
<RBS start="19515" stop="19530" startsite="19530" initstepsize="50" elongstepsize="" strength="1e7" stopsite="19834" protid="42" initRateConstant="0.3" mrnadeg="8.33e-3" />
<RBS start="19835" stop="19848" startsite="19848" initstepsize="50" elongstepsize="" strength="1e7" stopsite="20240" protid="43" initRateConstant="0.3" mrnadeg="8.33e-3" />
<RBS start="20224" stop="20239" startsite="20239" initstepsize="50" elongstepsize="" strength="1e7" stopsite="21850" protid="44" initRateConstant="0.3" mrnadeg="8.33e-3" />
<RBS start="21935" stop="21950" startsite="21950" initstepsize="50" elongstepsize="" strength="1e7" stopsite="22873" protid="45" initRateConstant="0.3" mrnadeg="1.11e-3" />
<RBS start="22942" stop="22967" startsite="22967" initstepsize="50" elongstepsize="" strength="1e7" stopsite="24004" protid="46" initRateConstant="0.3" mrnadeg="0" />
<RBS start="24213" stop="24228" startsite="24228" initstepsize="50" elongstepsize="" strength="1e7" stopsite="24818" protid="47" initRateConstant="0.3" mrnadeg="0" />
<RBS start="24827" stop="24842" startsite="24842" initstepsize="50" elongstepsize="" strength="1e7" stopsite="27226" protid="48" initRateConstant="0.3" mrnadeg="8.33e-3" />
<RBS start="27292" stop="27307" startsite="27307" initstepsize="50" elongstepsize="" strength="1e7" stopsite="27723" protid="49" initRateConstant="0.3" mrnadeg="8.33e-3" />
<RBS start="27707" stop="27722" startsite="27728" initstepsize="50" elongstepsize="" strength="1e7" stopsite="28318" protid="50" initRateConstant="0.3" mrnadeg="8.33e-3" />
<RBS start="28302" stop="28317" startsite="28325" initstepsize="50" elongstepsize="" strength="1e7" stopsite="30568" protid="51" initRateConstant="0.3" mrnadeg="0" />
<RBS start="30580" stop="30595" startsite="30595" initstepsize="50" elongstepsize="" strength="1e7" stopsite="34551" protid="52" initRateConstant="0.3" mrnadeg="2.38e-3" />
<RBS start="34609" stop="34624" startsite="34624" initstepsize="50" elongstepsize="" strength="1e7" stopsite="36285" protid="53" initRateConstant="0.3" mrnadeg="8.33e-3" />
<RBS start="36329" stop="36344" startsite="36344" initstepsize="50" elongstepsize="" strength="1e7" stopsite="36547" protid="54" initRateConstant="0.3" mrnadeg="1.11e-3" />
<RBS start="36531" stop="36546" startsite="36553" initstepsize="50" elongstepsize="" strength="1e7" stopsite="36822" protid="55" initRateConstant="0.3" mrnadeg="8.33e-3" />
<RBS start="36902" stop="36917" startsite="36917" initstepsize="50" elongstepsize="" strength="1e7" stopsite="37348" protid="56" initRateConstant="0.3" mrnadeg="8.33e-3" />
<RBS start="37355" stop="37370" startsite="37370" initstepsize="50" elongstepsize="" strength="1e7" stopsite="39130" protid="57" initRateConstant="0.3" mrnadeg="8.33e-3" />
<RBS start="39374" stop="39389" startsite="39389" initstepsize="50" elongstepsize="" strength="1e7" stopsite="39538" protid="58" initRateConstant="0.3" mrnadeg="8.33e-3" />
</DNA_SYSTEM>
</CELL>
</EXECUTE-SIMULATION>
</REQUEST>
