# Supplementary material for: TABASCO: A single molecule, base-pair resolved gene expression simulator
Source: BMC Bioinformatics. 2007 Dec 19;8:480. doi: 10.1186/1471-2105-8-480 (PMC2242808; doi:10.1186/1471-2105-8-480)
Supplement: Additional File 3 — TABASCO website. [file 1471-2105-8-480-S3.zip › doc/allclasses-frame.html]

All Classes


**All Classes**
  

|  |
| --- |
| Averager   Cell   DNA   GIntegrator   ImageToJpeg   Phage   PriorityQueue   Protein   RNA   Reaction   TabascoDraw   TabascoJpegMake   TabascoRead   TabascoReadMol   TabascoSimulator   TabascoWrite   TabascoXML   XMLObject |
