# Supplementary material for: TABASCO: A single molecule, base-pair resolved gene expression simulator
Source: BMC Bioinformatics. 2007 Dec 19;8:480. doi: 10.1186/1471-2105-8-480 (PMC2242808; doi:10.1186/1471-2105-8-480)
Supplement: Additional File 3 — TABASCO website. [file 1471-2105-8-480-S3.zip › doc/Averager.html]

Averager


|  |  |  |  |  |  |  |  |  |  |  |
| --- | --- | --- | --- | --- | --- | --- | --- | --- | --- | --- |
| |  |  |  |  |  |  |  | | --- | --- | --- | --- | --- | --- | --- | | Package | | **Class** | **Tree** | **Deprecated** | **Index** | **Help** | | | |  |
| PREV CLASS   **NEXT CLASS** | **FRAMES**    **NO FRAMES**     **All Classes** |
| SUMMARY: NESTED | FIELD | CONSTR | METHOD | DETAIL: FIELD | CONSTR | METHOD |


---


## Class Averager

```
java.lang.Object
  Averager
```

---

public class **Averager** extends java.lang.Object

Averages existing output files

**See Also:**: `TabascoSimulator`

---

|  |  |
| --- | --- |
| **Constructor Summary** | |
| `Averager()` |


|  |  |
| --- | --- |
| **Method Summary** | |
| `static void` | `main(java.lang.String[] args)`             Run from command line to average a set of output files. |

|  |
| --- |
| **Methods inherited from class java.lang.Object** |
| `clone, equals, finalize, getClass, hashCode, notify, notifyAll, toString, wait, wait, wait` |

|  |
| --- |
| **Constructor Detail** |

### Averager

```
public Averager()
```


|  |
| --- |
| **Method Detail** |

### main

```
public static void main(java.lang.String[] args)
```

:   Run from command line to average a set of output files. Outputs to text files in the directory the program is run.

    :   **Parameters:**: `args` - The command line input specifying the number of iterations, and the output file prefix. Assumes after the prefix are Mol\_sim#.txt or RNA\_sim#.txt.


---


|  |  |  |  |  |  |  |  |  |  |  |
| --- | --- | --- | --- | --- | --- | --- | --- | --- | --- | --- |
| |  |  |  |  |  |  |  | | --- | --- | --- | --- | --- | --- | --- | | Package | | **Class** | **Tree** | **Deprecated** | **Index** | **Help** | | | |  |
| PREV CLASS   **NEXT CLASS** | **FRAMES**    **NO FRAMES**     **All Classes** |
| SUMMARY: NESTED | FIELD | CONSTR | METHOD | DETAIL: FIELD | CONSTR | METHOD |


---
