# Supplementary material for: TABASCO: A single molecule, base-pair resolved gene expression simulator
Source: BMC Bioinformatics. 2007 Dec 19;8:480. doi: 10.1186/1471-2105-8-480 (PMC2242808; doi:10.1186/1471-2105-8-480)
Supplement: Additional File 3 — TABASCO website. [file 1471-2105-8-480-S3.zip › doc/Cell.html]

Cell


|  |  |  |  |  |  |  |  |  |  |  |
| --- | --- | --- | --- | --- | --- | --- | --- | --- | --- | --- |
| |  |  |  |  |  |  |  | | --- | --- | --- | --- | --- | --- | --- | | Package | | **Class** | **Tree** | **Deprecated** | **Index** | **Help** | | | |  |
| **PREV CLASS**   **NEXT CLASS** | **FRAMES**    **NO FRAMES**     **All Classes** |
| SUMMARY: NESTED | FIELD | CONSTR | METHOD | DETAIL: FIELD | CONSTR | METHOD |


---


## Class Cell

```
java.lang.Object
  Cell
```

---

public class **Cell** extends java.lang.Object

A class representing a cell.
The cell class contains the arrays for the Molecules in the cell, the cell volume, and
how the transcribing polymerases interact on the DNA. Also includes the pointers to
the DNA/phage that are entering or currently in the cell.

**See Also:**: `Molecule`, `Phage`

---

|  |  |
| --- | --- |
| **Constructor Summary** | |
| `Cell(TabascoSimulator mysim)`             Creates an empty Cell structure. |


|  |  |
| --- | --- |
| **Method Summary** | |
| `long[]` | `resetTranscriptionEvents()`             Resets the counter on the number of transcription events that have taken place for each polymerase. |

|  |
| --- |
| **Methods inherited from class java.lang.Object** |
| `clone, equals, finalize, getClass, hashCode, notify, notifyAll, toString, wait, wait, wait` |

|  |
| --- |
| **Constructor Detail** |

### Cell

```
public Cell(TabascoSimulator mysim)
     throws java.io.IOException
```

:   Creates an empty Cell structure. In order to fill the contents of this
    class, the TabascoXML.fillCell() method needs to be invoked.

    **Parameters:**: `mysim` - The pointer to the simulator that this cell resides in. **See Also:**: `TabascoXML#fillCell()`


|  |
| --- |
| **Method Detail** |

### resetTranscriptionEvents

```
public long[] resetTranscriptionEvents()
```

:   Resets the counter on the number of transcription events that have taken place for each polymerase.

    :   **Returns:**: a vector containing the number of transcription elongation events that have occured since the method was last invoked


---


|  |  |  |  |  |  |  |  |  |  |  |
| --- | --- | --- | --- | --- | --- | --- | --- | --- | --- | --- |
| |  |  |  |  |  |  |  | | --- | --- | --- | --- | --- | --- | --- | | Package | | **Class** | **Tree** | **Deprecated** | **Index** | **Help** | | | |  |
| **PREV CLASS**   **NEXT CLASS** | **FRAMES**    **NO FRAMES**     **All Classes** |
| SUMMARY: NESTED | FIELD | CONSTR | METHOD | DETAIL: FIELD | CONSTR | METHOD |


---
