# Supplementary material for: TABASCO: A single molecule, base-pair resolved gene expression simulator
Source: BMC Bioinformatics. 2007 Dec 19;8:480. doi: 10.1186/1471-2105-8-480 (PMC2242808; doi:10.1186/1471-2105-8-480)
Supplement: Additional File 3 — TABASCO website. [file 1471-2105-8-480-S3.zip › doc/DNA.html]

DNA


|  |  |  |  |  |  |  |  |  |  |  |
| --- | --- | --- | --- | --- | --- | --- | --- | --- | --- | --- |
| |  |  |  |  |  |  |  | | --- | --- | --- | --- | --- | --- | --- | | Package | | **Class** | **Tree** | **Deprecated** | **Index** | **Help** | | | |  |
| **PREV CLASS**   **NEXT CLASS** | **FRAMES**    **NO FRAMES**     **All Classes** |
| SUMMARY: NESTED | FIELD | CONSTR | METHOD | DETAIL: FIELD | CONSTR | METHOD |


---


## Class DNA

```
java.lang.Object
  DNA
```

**All Implemented Interfaces:**: Molecule

---

public class **DNA** extends java.lang.Object implements Molecule

Generic class that describes features on a piece of DNA (or Phage).

**See Also:**: `Phage`

---

|  |  |
| --- | --- |
| **Constructor Summary** | |
| `DNA(int start, int stop, int ID)`             The default constructure of a DNA feature. |


|  |  |
| --- | --- |
| **Method Summary** | |
| `int` | `getCopyNumber()`             Retrieves the copy number of the DNA feature. |
| `Reaction` | `getEntryReaction()`             Retrieves the reaction to execute upon unblocking or entry of the DNA feature. |
| `int` | `getID()`             Retrieves the ID of this DNA feature. |
| `Reaction` | `getLossReaction()`             Retrieves the reaction to execute upon blocking by a DPComplex. |
| `int` | `getStart()`             Retrieves the start position of the DNA feature. |
| `int` | `getStop()`             Retrieves the stop position of the DNA feature. |
| `void` | `incrementCopyNumber(int inc)`             Increments the copy number of the DNA feature. |
| `void` | `setEntryReaction(Reaction rec)`             Sets the entryReaction as teh reaction that is input. |
| `void` | `setLossReaction(Reaction rec)`             Sets the lossReaction as the reaction that is input. |

|  |
| --- |
| **Methods inherited from class java.lang.Object** |
| `clone, equals, finalize, getClass, hashCode, notify, notifyAll, toString, wait, wait, wait` |

|  |
| --- |
| **Constructor Detail** |

### DNA

```
public DNA(int start,
           int stop,
           int ID)
```

:   The default constructure of a DNA feature.

    **Parameters:**: `start` - the most upstream position of the DNA feature: `stop` - the most downstream position of the DNA feature.: `ID` - the ID of the DNA feature.


|  |
| --- |
| **Method Detail** |

### getStart

```
public int getStart()
```

:   Retrieves the start position of the DNA feature.

    :   **Returns:**: the start position of the DNA feature.

---


### getStop

```
public int getStop()
```

:   Retrieves the stop position of the DNA feature.

    :   **Returns:**: the start position of the DNA feature

---


### getCopyNumber

```
public int getCopyNumber()
```

:   Retrieves the copy number of the DNA feature.

    :   **Specified by:**: `getCopyNumber` in interface `Molecule`
    :   **Returns:**: the copy number of the DNA feature

---


### incrementCopyNumber

```
public void incrementCopyNumber(int inc)
```

:   Increments the copy number of the DNA feature.

    :   **Specified by:**: `incrementCopyNumber` in interface `Molecule`
    :   **Parameters:**: `inc` - the value to increment the current copyNumber by

---


### getID

```
public int getID()
```

:   Retrieves the ID of this DNA feature.

    :   **Specified by:**: `getID` in interface `Molecule`
    :   **Returns:**: the ID of this DNA feature

---


### getLossReaction

```
public Reaction getLossReaction()
```

:   Retrieves the reaction to execute upon blocking by a DPComplex.

    :   **Returns:**: the reaction to execute upon blocking by a DPComplex

---


### setLossReaction

```
public void setLossReaction(Reaction rec)
```

:   Sets the lossReaction as the reaction that is input.

    :   **Parameters:**: `rec` - the reaction that is to be set as this DNA feature's lossReaction

---


### getEntryReaction

```
public Reaction getEntryReaction()
```

:   Retrieves the reaction to execute upon unblocking or entry of the DNA feature.

    :   **Returns:**: the reaction to execute upon unblocking or entry of the DNA feature.

---


### setEntryReaction

```
public void setEntryReaction(Reaction rec)
```

:   Sets the entryReaction as teh reaction that is input.

    :   **Parameters:**: `rec` - the reaction that is to be set as this DNA feature's entryReaction


---


|  |  |  |  |  |  |  |  |  |  |  |
| --- | --- | --- | --- | --- | --- | --- | --- | --- | --- | --- |
| |  |  |  |  |  |  |  | | --- | --- | --- | --- | --- | --- | --- | | Package | | **Class** | **Tree** | **Deprecated** | **Index** | **Help** | | | |  |
| **PREV CLASS**   **NEXT CLASS** | **FRAMES**    **NO FRAMES**     **All Classes** |
| SUMMARY: NESTED | FIELD | CONSTR | METHOD | DETAIL: FIELD | CONSTR | METHOD |


---
