# Supplementary material for: TABASCO: A single molecule, base-pair resolved gene expression simulator
Source: BMC Bioinformatics. 2007 Dec 19;8:480. doi: 10.1186/1471-2105-8-480 (PMC2242808; doi:10.1186/1471-2105-8-480)
Supplement: Additional File 3 — TABASCO website. [file 1471-2105-8-480-S3.zip › doc/GIntegrator.html]

GIntegrator


|  |  |  |  |  |  |  |  |  |  |  |
| --- | --- | --- | --- | --- | --- | --- | --- | --- | --- | --- |
| |  |  |  |  |  |  |  | | --- | --- | --- | --- | --- | --- | --- | | Package | | **Class** | **Tree** | **Deprecated** | **Index** | **Help** | | | |  |
| **PREV CLASS**   **NEXT CLASS** | **FRAMES**    **NO FRAMES**     **All Classes** |
| SUMMARY: NESTED | FIELD | CONSTR | METHOD | DETAIL: FIELD | CONSTR | METHOD |


---


## Class GIntegrator

```
java.lang.Object
  GIntegrator
```

---

public class **GIntegrator** extends java.lang.Object

Class that orchestrates the Gillespie-based integrator and stores the priority queue.

**See Also:**: `IndexedPriorityQueue`

---

|  |  |
| --- | --- |
| **Constructor Summary** | |
| `GIntegrator(Reaction[] thereacs, long key)`             The only constructor of GIntegrator. |


|  |  |
| --- | --- |
| **Method Summary** | |
| `void` | `runLoop()`             The main loop that is run find the next reaction to execute, update the time, execute the reaction, and reinsert into the priority queue. |

|  |
| --- |
| **Methods inherited from class java.lang.Object** |
| `clone, equals, finalize, getClass, hashCode, notify, notifyAll, toString, wait, wait, wait` |

|  |
| --- |
| **Constructor Detail** |

### GIntegrator

```
public GIntegrator(Reaction[] thereacs,
                   long key)
```

:   The only constructor of GIntegrator.

    **Parameters:**: `thereacs` - A vector of reactions that are defined at the start of simulation using TabascoXML.: `key` - A key to create the random number generator.


|  |
| --- |
| **Method Detail** |

### runLoop

```
public void runLoop()
```

:   The main loop that is run find the next reaction to execute, update the time, execute the reaction, and reinsert into the priority queue.


---


|  |  |  |  |  |  |  |  |  |  |  |
| --- | --- | --- | --- | --- | --- | --- | --- | --- | --- | --- |
| |  |  |  |  |  |  |  | | --- | --- | --- | --- | --- | --- | --- | | Package | | **Class** | **Tree** | **Deprecated** | **Index** | **Help** | | | |  |
| **PREV CLASS**   **NEXT CLASS** | **FRAMES**    **NO FRAMES**     **All Classes** |
| SUMMARY: NESTED | FIELD | CONSTR | METHOD | DETAIL: FIELD | CONSTR | METHOD |


---
