# Supplementary material for: TABASCO: A single molecule, base-pair resolved gene expression simulator
Source: BMC Bioinformatics. 2007 Dec 19;8:480. doi: 10.1186/1471-2105-8-480 (PMC2242808; doi:10.1186/1471-2105-8-480)
Supplement: Additional File 3 — TABASCO website. [file 1471-2105-8-480-S3.zip › doc/ImageToJpeg.html]

ImageToJpeg


|  |  |  |  |  |  |  |  |  |  |  |
| --- | --- | --- | --- | --- | --- | --- | --- | --- | --- | --- |
| |  |  |  |  |  |  |  | | --- | --- | --- | --- | --- | --- | --- | | Package | | **Class** | **Tree** | **Deprecated** | **Index** | **Help** | | | |  |
| **PREV CLASS**   **NEXT CLASS** | **FRAMES**    **NO FRAMES**     **All Classes** |
| SUMMARY: NESTED | FIELD | CONSTR | METHOD | DETAIL: FIELD | CONSTR | METHOD |


---


## Class ImageToJpeg

```
java.lang.Object
  ImageToJpeg
```

---

public class **ImageToJpeg** extends java.lang.Object

A class that is used to convert an image into jpeg format. This code was derived from a developer.com article by Benoit Marchal "JDK 1.2 does JPEG" 11/25/1998

---

|  |  |
| --- | --- |
| **Constructor Summary** | |
| `ImageToJpeg()` |


|  |  |
| --- | --- |
| **Method Summary** | |
| `static void` | `EncodeIt(java.awt.image.BufferedImage img, java.io.OutputStream out)` |

|  |
| --- |
| **Methods inherited from class java.lang.Object** |
| `clone, equals, finalize, getClass, hashCode, notify, notifyAll, toString, wait, wait, wait` |

|  |
| --- |
| **Constructor Detail** |

### ImageToJpeg

```
public ImageToJpeg()
```


|  |
| --- |
| **Method Detail** |

### EncodeIt

```
public static void EncodeIt(java.awt.image.BufferedImage img,
                            java.io.OutputStream out)
                     throws java.io.IOException
```

:   **Throws:**: `java.io.IOException`


---


|  |  |  |  |  |  |  |  |  |  |  |
| --- | --- | --- | --- | --- | --- | --- | --- | --- | --- | --- |
| |  |  |  |  |  |  |  | | --- | --- | --- | --- | --- | --- | --- | | Package | | **Class** | **Tree** | **Deprecated** | **Index** | **Help** | | | |  |
| **PREV CLASS**   **NEXT CLASS** | **FRAMES**    **NO FRAMES**     **All Classes** |
| SUMMARY: NESTED | FIELD | CONSTR | METHOD | DETAIL: FIELD | CONSTR | METHOD |


---
