# Supplementary material for: TABASCO: A single molecule, base-pair resolved gene expression simulator
Source: BMC Bioinformatics. 2007 Dec 19;8:480. doi: 10.1186/1471-2105-8-480 (PMC2242808; doi:10.1186/1471-2105-8-480)
Supplement: Additional File 3 — TABASCO website. [file 1471-2105-8-480-S3.zip › doc/index-all.html]

Index


|  |  |  |  |  |  |  |  |  |  |
| --- | --- | --- | --- | --- | --- | --- | --- | --- | --- |
| |  |  |  |  |  |  | | --- | --- | --- | --- | --- | --- | | Package | Class | **Tree** | **Deprecated** | **Index** | **Help** | | | |  |
| PREV   NEXT | **FRAMES**    **NO FRAMES**     **All Classes** |


A C D E F G I L M N P R S T W X 

---


## **A**

**Averager** - class Averager.: Averages existing output files **Averager()** - Constructor for class Averager: **addChild(XMLObject)** - Method in class XMLObject: Adds a child node **addChild(String)** - Method in class XMLObject: Adds a child node **affects()** - Method in class Reaction: Returns all the molecules that are affected upon the execution of this reaction. **attributes** - Variable in class XMLObject

---


## **C**

**Cell** - class Cell.: A class representing a cell. **Cell(TabascoSimulator)** - Constructor for class Cell: Creates an empty Cell structure. **calculateA()** - Method in class Reaction: Calculate the propensity of the reaction. **calculateTime(double, double)** - Method in class Reaction: Calculate the time the reaction will next execute. **childrenNodes** - Variable in class XMLObject: **cleanUp()** - Method in class TabascoRead: **cleanUp()** - Method in class TabascoWrite: **clearMemFast()** - Method in class TabascoRead: **close()** - Method in class TabascoReadMol: **combineData(int, String, String, String)** - Static method in class TabascoSimulator: Averages the molecule file data across multiple simulations **combineEnergyData(int, String, String, String, int)** - Static method in class TabascoSimulator: Averages the energy output files from individual simulations. **combineInitRnaData(int, String, String, String, int)** - Static method in class TabascoSimulator: Averages the RNA initiation output files from individual simulations. **compareTo(Reaction)** - Method in class Reaction: Compares this reaction time to execute to another reaction. **contents** - Variable in class XMLObject: **convertFeatureToElement(int)** - Method in class Phage: Takes a position on the DNA and returns what DNA feature is encoded upon it, if any. **copyNumber** - Variable in class Protein: The number of copies of the protein **createTrackers()** - Method in class Phage: A method that is run at the creation of the variables that track the DNA at single base resolution.

---


## **D**

**DNA** - class DNA.: Generic class that describes features on a piece of DNA (or Phage). **DNA(int, int, int)** - Constructor for class DNA: The default constructure of a DNA feature. **delete(int)** - Method in class PriorityQueue: Removes an object from the priority queue **deleteMin()** - Method in class PriorityQueue: Remove the smallest item from the priority queue. **dependsOn()** - Method in class Reaction: Returns the set of reactants that this reaction depends on.

---


## **E**

**EncodeIt(BufferedImage, OutputStream)** - Static method in class ImageToJpeg: **elementAt(int)** - Method in class PriorityQueue: Retrieve a particular element in the priority queue **execute()** - Method in class Reaction: Executes the reaction.

---


## **F**

**fillCell(Cell, XMLObject, int)** - Static method in class TabascoXML: Parses parameters to initialize the Cell class **fillPhage(Cell, Phage, XMLObject, int)** - Static method in class TabascoXML: Parses parameters to initialize the Phage class **fillPhageReactions(Phage, int, int)** - Static method in class TabascoXML: Parses parameters to fill in reactions from phage class **fillSim(TabascoSimulator, XMLObject)** - Static method in class TabascoXML: Parses parameters to initialize the Simulator class **findAffects(Reaction)** - Method in class Reaction: A method that is used upon initialization to populate the updateRxn vector. **findMin()** - Method in class PriorityQueue: Find the smallest item in the priority queue.

---


## **G**

**GENOME\_LENGTH** - Variable in class Phage: The length of the DNA molecule that this instance of the Phage class represents. **GIntegrator** - class GIntegrator.: Class that orchestrates the Gillespie-based integrator and stores the priority queue. **GIntegrator(Reaction[], long)** - Constructor for class GIntegrator: The only constructor of GIntegrator. **getA()** - Method in class Reaction: Returns the current propensity of the reaction. **getAttribute(String)** - Method in class XMLObject: Returns the value of the attribute with the key given **getAttributes()** - Method in class XMLObject: Returns the attributes of this XML Object **getChild(String)** - Method in class XMLObject: **getChildren()** - Method in class XMLObject: Returns the XML children nodes for this object **getComplexArrayFast()** - Method in class TabascoRead: **getContents()** - Method in class XMLObject: **getCopyNumber()** - Method in class DNA: Retrieves the copy number of the DNA feature. **getCopyNumber()** - Method in class Protein: Returns the number of copies of the protein **getCopyNumber()** - Method in class RNA: Returns the current copy number of the RNA **getCustomProtNames()** - Method in class TabascoReadMol: **getCustomProteinCopyNumbers()** - Method in class TabascoReadMol: **getDNAArray()** - Method in class TabascoRead: **getEnteredDNA(int)** - Method in class TabascoRead: **getEnteredDNAFast()** - Method in class TabascoRead: **getEntryReaction()** - Method in class DNA: Retrieves the reaction to execute upon unblocking or entry of the DNA feature. **getGenomeSize()** - Method in class TabascoRead: **getID()** - Method in class DNA: Retrieves the ID of this DNA feature. **getID()** - Method in class Protein: Returns the ID of the protein. **getID()** - Method in class RNA: Returns the ID of the RNA. **getID()** - Method in class Reaction: Returns the ID of the reaction. **getLength()** - Method in class RNA: Returns the current length of the RNA. **getLossReaction()** - Method in class DNA: Retrieves the reaction to execute upon blocking by a DPComplex. **getMRNACopyNumber(int, int)** - Method in class TabascoRead: **getMRNACopyNumberFast()** - Method in class TabascoRead: **getMRNALength(int, int)** - Method in class TabascoRead: **getMRNALengthFast()** - Method in class TabascoRead: **getMRNAStart(int, int)** - Method in class TabascoRead: **getMRNAStartFast()** - Method in class TabascoRead: **getNVPairs(String, String, String)** - Method in class XMLObject: **getName()** - Method in class Protein: Returns the name of the protein. **getName()** - Method in class XMLObject: Returns the name of this XML Object **getNamedChildren(String)** - Method in class XMLObject: **getNumOfTimePoints()** - Method in class TabascoReadMol: **getNumberOfCodingRgns()** - Method in class TabascoRead: **getNumberOfColiPolymerase()** - Method in class TabascoReadMol: **getNumberOfRNA()** - Method in class TabascoReadMol: **getNumberOfT7Polymerase()** - Method in class TabascoReadMol: **getOrganism()** - Method in class Protein: Returns the organism of the protein. **getPolymeraseCopyNumbers()** - Method in class TabascoReadMol: **getPolymeraseNames()** - Method in class TabascoReadMol: **getProperty(String)** - Method in class XMLObject: **getRNACopyNumbers()** - Method in class TabascoReadMol: **getRNANames()** - Method in class TabascoReadMol: **getRibosomeCopyNumber()** - Method in class TabascoReadMol: **getStart()** - Method in class DNA: Retrieves the start position of the DNA feature. **getStart()** - Method in class RNA: Returns the most upstream position of the RNA in the DNA coordinates from where it is transcribed. **getStop()** - Method in class DNA: Retrieves the stop position of the DNA feature. **getStop()** - Method in class RNA: Returns the most dowsntream position of the RNA in the DNA coordinates from where it is transcribed. **getTime()** - Method in class Reaction: Returns the time that this reaction is next expected to execute. **getTime(int)** - Method in class TabascoRead: **getTime()** - Method in class TabascoReadMol: **getTimeEnd()** - Method in class TabascoRead: **getTimeFast()** - Method in class TabascoRead: **getTimePoints()** - Method in class TabascoRead: **getTimeStep()** - Method in class TabascoRead

---


## **I**

**ID** - Variable in class Reaction: The reactions ID. **ImageToJpeg** - class ImageToJpeg.: A class that is used to convert an image into jpeg format. **ImageToJpeg()** - Constructor for class ImageToJpeg: **IsThereNextComplexArray()** - Method in class TabascoRead: **incrementCopyNumber(int)** - Method in class DNA: Increments the copy number of the DNA feature. **incrementCopyNumber(int)** - Method in class Protein: Incremements the copy number of the protein by inc. **incrementCopyNumber(int)** - Method in class RNA: Increments the number of copies of RNA by inc. **insert(Comparable)** - Method in class PriorityQueue: Insert into the priority queue. **isEmpty()** - Method in class PriorityQueue: Test if the priority queue is logically empty.

---


## **L**

**loadNextDNAArrayFast()** - Method in class TabascoRead: **loadNextMoleculeArrayFast()** - Method in class TabascoReadMol

---


## **M**

**main(String[])** - Static method in class Averager: Run from command line to average a set of output files. **main(String[])** - Static method in class TabascoJpegMake: The class that is called when running this class from the command-line. **main(String[])** - Static method in class TabascoReadMol: **main(String[])** - Static method in class TabascoSimulator: The method run from the command line to run Tabasco Simulations. **makeDNAGraphic(Graphics2D, String[], int, int, int, int, int, int, int, int, int, int, int, int[])** - Static method in class TabascoDraw: This method takes a Graphics2D object and adds a representation of the DNA with complexes and genetic elements annotated onto it. **makeEmpty()** - Method in class PriorityQueue: Make the priority queue logically empty. **makeMRNAGraphic(Graphics2D, int[], int[], int[], int, int, Color[], int, int)** - Static method in class TabascoDraw: This method takes in a Graphics2D object and draws an image depicting the nascentRNA levels of all the coding domains on the DNA. **makeMoleculeGraph(String[], int[], Color[], double, int, int, int[], BufferedImage)** - Method in class TabascoDraw: This method takes in an image, and returns one with a molecule graph added. **makeSingleGraphic(String[], int[], int[], int[], int, int, int, BufferedImage, int[])** - Static method in class TabascoDraw: This is the method that sets the characteristics of the DNA visualization, and runs the other methods in this class within it.

---


## **N**

**name** - Variable in class XMLObject

---


## **P**

**Phage** - class Phage.: The class that is a container for the DNA being simulated. **Phage(Cell, int)** - Constructor for class Phage: The only constructor for a Phage instance. **PriorityQueue** - class PriorityQueue.: **PriorityQueue()** - Constructor for class PriorityQueue: Construct the binary heap. **Protein** - class Protein.: A class for proteins that are contained in the cell. **Protein(String, int, int, int)** - Constructor for class Protein: **print()** - Method in class XMLObject: Returns a string containing the XML text representation of this object

---


## **R**

**RNA** - class RNA.: General class for RNA molecules. **RNA(int, int)** - Constructor for class RNA: A constructor for an RNA molecule **RNA(int, int, int)** - Constructor for class RNA: A constructor for an RNA molecule **RNA(int)** - Constructor for class RNA: A constructor for an RNA molecule **Reaction** - class Reaction.: A general class for Reactions. **Reaction(int, double)** - Constructor for class Reaction: A default empty constructor. **Reaction(int, Molecule[], Molecule[], double)** - Constructor for class Reaction: A constructor that takes in reactant and product molecules as well. **removeAttribute(String)** - Method in class XMLObject: Removes an attribute with the key given **resetBr(BufferedReader, String)** - Method in class TabascoRead: **resetTranscriptionEvents()** - Method in class Cell: Resets the counter on the number of transcription events that have taken place for each polymerase. **returnTokenRow(int)** - Method in class TabascoRead: **runLoop()** - Method in class GIntegrator: The main loop that is run find the next reaction to execute, update the time, execute the reaction, and reinsert into the priority queue.

---


## **S**

**setAttribute(String, String)** - Method in class XMLObject: Sets an Attribute for the XML object **setAttributes(Properties)** - Method in class XMLObject: **setContents(String)** - Method in class XMLObject: **setEntryReaction(Reaction)** - Method in class DNA: Sets the entryReaction as teh reaction that is input. **setLossReaction(Reaction)** - Method in class DNA: Sets the lossReaction as the reaction that is input. **setName(String)** - Method in class XMLObject: Sets the name of this XML Object **setProperty(String, String)** - Method in class XMLObject: **setTime(double)** - Method in class Reaction: Sets the time that this reaction is next expected to execute. **size()** - Method in class PriorityQueue: Returns size.

---


## **T**

**TabascoDraw** - class TabascoDraw.: Tabasco Draw is part of the visualization suite for the Tabasco stochastic simulator. **TabascoDraw(TabascoRead)** - Constructor for class TabascoDraw: The only constructor for this class. **TabascoJpegMake** - class TabascoJpegMake.: TabascoJpegMake is class to make visualization of the output by making a stack of images of individual timepoints. **TabascoJpegMake()** - Constructor for class TabascoJpegMake: **TabascoRead** - class TabascoRead.: TabascoRead is the class that is used to read DNA output files of simulations. **TabascoRead(String)** - Constructor for class TabascoRead: **TabascoReadMol** - class TabascoReadMol.: TabascoReadMol is the class that is used to read molecule output files of simulations. **TabascoReadMol(String)** - Constructor for class TabascoReadMol: **TabascoSimulator** - class TabascoSimulator.: TabascoSimulator is the class used to run simulations and produce output files. **TabascoSimulator()** - Constructor for class TabascoSimulator: **TabascoWrite** - class TabascoWrite.: TabascoWrite is the class that is used to write DNA output files of simulations. **TabascoWrite(String)** - Constructor for class TabascoWrite: **TabascoXML** - class TabascoXML.: TabascoXML parses the XML input file that specifies simulation parameters **TabascoXML()** - Constructor for class TabascoXML

---


## **W**

**writeComplexes(DPComplex[])** - Method in class TabascoWrite: **writeDNAFeatures(short[])** - Method in class TabascoWrite: **writeEntry(int)** - Method in class TabascoWrite: **writeFiles(String[], String, String)** - Static method in class TabascoJpegMake: The main method used to write files. **writeFirstLine(Phage)** - Method in class TabascoWrite: **writeGenomeSize(int)** - Method in class TabascoWrite: **writeMRNA(Molecule[], Phage, int, int, int)** - Method in class TabascoWrite: **writeMainLine(double, Phage, int)** - Method in class TabascoWrite: **writeNumberOfCodingRgns(int)** - Method in class TabascoWrite: **writeNumberOfTimepoints(int)** - Method in class TabascoWrite: **writeTime(double)** - Method in class TabascoWrite: **writeTimeEnd(int)** - Method in class TabascoWrite: **writeTimeStep(int)** - Method in class TabascoWrite

---


## **X**

**XMLObject** - class XMLObject.: **XMLObject()** - Constructor for class XMLObject: **XMLObject(String)** - Constructor for class XMLObject: Main constructor for an XML Object, takes the XML text and creates an XML Object encapsulating and enabling access to taht data **XMLObject(File)** - Constructor for class XMLObject

---

A C D E F G I L M N P R S T W X


|  |  |  |  |  |  |  |  |  |  |
| --- | --- | --- | --- | --- | --- | --- | --- | --- | --- |
| |  |  |  |  |  |  | | --- | --- | --- | --- | --- | --- | | Package | Class | **Tree** | **Deprecated** | **Index** | **Help** | | | |  |
| PREV   NEXT | **FRAMES**    **NO FRAMES**     **All Classes** |


---
