# Supplementary material for: TABASCO: A single molecule, base-pair resolved gene expression simulator
Source: BMC Bioinformatics. 2007 Dec 19;8:480. doi: 10.1186/1471-2105-8-480 (PMC2242808; doi:10.1186/1471-2105-8-480)
Supplement: Additional File 3 — TABASCO website. [file 1471-2105-8-480-S3.zip › doc/overview-tree.html]

Class Hierarchy


|  |  |  |  |  |  |  |  |  |  |
| --- | --- | --- | --- | --- | --- | --- | --- | --- | --- |
| |  |  |  |  |  |  | | --- | --- | --- | --- | --- | --- | | Package | Class | **Tree** | **Deprecated** | **Index** | **Help** | | | |  |
| PREV   NEXT | **FRAMES**    **NO FRAMES**     **All Classes** |


---


## Hierarchy For All Packages

## Class Hierarchy

- class java.lang.Object
  - class **Averager**- class **Cell**- class **DNA** (implements Molecule)- class **GIntegrator**- class **ImageToJpeg**- class **Phage**- class **PriorityQueue**- class **Protein** (implements Molecule)- class **Reaction**- class **RNA** (implements Molecule)- class **TabascoDraw**- class **TabascoJpegMake**- class **TabascoRead**- class **TabascoReadMol**- class **TabascoSimulator**- class **TabascoWrite**- class **TabascoXML**- class **XMLObject**

---


|  |  |  |  |  |  |  |  |  |  |
| --- | --- | --- | --- | --- | --- | --- | --- | --- | --- |
| |  |  |  |  |  |  | | --- | --- | --- | --- | --- | --- | | Package | Class | **Tree** | **Deprecated** | **Index** | **Help** | | | |  |
| PREV   NEXT | **FRAMES**    **NO FRAMES**     **All Classes** |


---
