# Supplementary material for: TABASCO: A single molecule, base-pair resolved gene expression simulator
Source: BMC Bioinformatics. 2007 Dec 19;8:480. doi: 10.1186/1471-2105-8-480 (PMC2242808; doi:10.1186/1471-2105-8-480)
Supplement: Additional File 3 — TABASCO website. [file 1471-2105-8-480-S3.zip › doc/Phage.html]

Phage


|  |  |  |  |  |  |  |  |  |  |  |
| --- | --- | --- | --- | --- | --- | --- | --- | --- | --- | --- |
| |  |  |  |  |  |  |  | | --- | --- | --- | --- | --- | --- | --- | | Package | | **Class** | **Tree** | **Deprecated** | **Index** | **Help** | | | |  |
| **PREV CLASS**   **NEXT CLASS** | **FRAMES**    **NO FRAMES**     **All Classes** |
| SUMMARY: NESTED | FIELD | CONSTR | METHOD | DETAIL: FIELD | CONSTR | METHOD |


---


## Class Phage

```
java.lang.Object
  Phage
```

---

public class **Phage** extends java.lang.Object

The class that is a container for the DNA being simulated. Each instance of this class within a cell has its own
set of genetic elements and mapping to position, set of complexes on the DNA, et cetera.

**See Also:**: `Cell`

---

|  |  |
| --- | --- |
| **Field Summary** | |
| `int` | `GENOME_LENGTH`             The length of the DNA molecule that this instance of the Phage class represents. |


|  |  |
| --- | --- |
| **Constructor Summary** | |
| `Phage(Cell mycell, int ID)`             The only constructor for a Phage instance. |


|  |  |
| --- | --- |
| **Method Summary** | |
| `DNA` | `convertFeatureToElement(int featurePosition)`             Takes a position on the DNA and returns what DNA feature is encoded upon it, if any. |
| `protected  void` | `createTrackers()`             A method that is run at the creation of the variables that track the DNA at single base resolution. |

|  |
| --- |
| **Methods inherited from class java.lang.Object** |
| `clone, equals, finalize, getClass, hashCode, notify, notifyAll, toString, wait, wait, wait` |

|  |
| --- |
| **Field Detail** |

### GENOME\_LENGTH

```
public int GENOME_LENGTH
```

:   The length of the DNA molecule that this instance of the Phage class represents.


|  |
| --- |
| **Constructor Detail** |

### Phage

```
public Phage(Cell mycell,
             int ID)
```

:   The only constructor for a Phage instance.

    **Parameters:**: `mycell` - The Cell that this Phage/DNA is contained within: `ID` - The ID of the Phage/DNA


|  |
| --- |
| **Method Detail** |

### convertFeatureToElement

```
public DNA convertFeatureToElement(int featurePosition)
```

:   Takes a position on the DNA and returns what DNA feature is encoded upon it, if any.

    :   **Parameters:**: `featurePosition` - the position on the DNA

---


### createTrackers

```
protected void createTrackers()
```

:   A method that is run at the creation of the variables that track the DNA at single base resolution.


---


|  |  |  |  |  |  |  |  |  |  |  |
| --- | --- | --- | --- | --- | --- | --- | --- | --- | --- | --- |
| |  |  |  |  |  |  |  | | --- | --- | --- | --- | --- | --- | --- | | Package | | **Class** | **Tree** | **Deprecated** | **Index** | **Help** | | | |  |
| **PREV CLASS**   **NEXT CLASS** | **FRAMES**    **NO FRAMES**     **All Classes** |
| SUMMARY: NESTED | FIELD | CONSTR | METHOD | DETAIL: FIELD | CONSTR | METHOD |


---
