# Supplementary material for: TABASCO: A single molecule, base-pair resolved gene expression simulator
Source: BMC Bioinformatics. 2007 Dec 19;8:480. doi: 10.1186/1471-2105-8-480 (PMC2242808; doi:10.1186/1471-2105-8-480)
Supplement: Additional File 3 — TABASCO website. [file 1471-2105-8-480-S3.zip › doc/PriorityQueue.html]

PriorityQueue


|  |  |  |  |  |  |  |  |  |  |  |
| --- | --- | --- | --- | --- | --- | --- | --- | --- | --- | --- |
| |  |  |  |  |  |  |  | | --- | --- | --- | --- | --- | --- | --- | | Package | | **Class** | **Tree** | **Deprecated** | **Index** | **Help** | | | |  |
| **PREV CLASS**   **NEXT CLASS** | **FRAMES**    **NO FRAMES**     **All Classes** |
| SUMMARY: NESTED | FIELD | CONSTR | METHOD | DETAIL: FIELD | CONSTR | METHOD |


---


## Class PriorityQueue

```
java.lang.Object
  PriorityQueue
```

---

public class **PriorityQueue** extends java.lang.Object

---

|  |  |
| --- | --- |
| **Constructor Summary** | |
| `PriorityQueue()`             Construct the binary heap. |


|  |  |
| --- | --- |
| **Method Summary** | |
| `java.lang.Comparable` | `delete(int hole)`             Removes an object from the priority queue |
| `java.lang.Comparable` | `deleteMin()`             Remove the smallest item from the priority queue. |
| `java.lang.Comparable` | `elementAt(int i)`             Retrieve a particular element in the priority queue |
| `java.lang.Comparable` | `findMin()`             Find the smallest item in the priority queue. |
| `void` | `insert(java.lang.Comparable x)`             Insert into the priority queue. |
| `boolean` | `isEmpty()`             Test if the priority queue is logically empty. |
| `void` | `makeEmpty()`             Make the priority queue logically empty. |
| `int` | `size()`             Returns size. |

|  |
| --- |
| **Methods inherited from class java.lang.Object** |
| `clone, equals, finalize, getClass, hashCode, notify, notifyAll, toString, wait, wait, wait` |

|  |
| --- |
| **Constructor Detail** |

### PriorityQueue

```
public PriorityQueue()
```

:   Construct the binary heap.


|  |
| --- |
| **Method Detail** |

### insert

```
public void insert(java.lang.Comparable x)
```

:   Insert into the priority queue.
    Duplicates are allowed.

    :   **Parameters:**: `x` - the item to insert. **Returns:**: null, signifying that decreaseKey cannot be used.

---


### elementAt

```
public java.lang.Comparable elementAt(int i)
                               throws java.io.IOException
```

:   Retrieve a particular element in the priority queue

    :   **Parameters:**: `i` - the index **Returns:**: the Comparable element requested **Throws:**: `java.io.IOException` - if index is outside valid indices.

---


### findMin

```
public java.lang.Comparable findMin()
                             throws java.io.IOException
```

:   Find the smallest item in the priority queue.

    :   **Returns:**: the smallest item. **Throws:**: `UnderflowException` - if empty.: `java.io.IOException`

---


### deleteMin

```
public java.lang.Comparable deleteMin()
                               throws java.io.IOException
```

:   Remove the smallest item from the priority queue.

    :   **Returns:**: the smallest item. **Throws:**: `UnderflowException` - if empty.: `java.io.IOException`

---


### delete

```
public java.lang.Comparable delete(int hole)
                            throws java.io.IOException
```

:   Removes an object from the priority queue

    :   **Returns:**: the deleted item **Throws:**: `java.io.IOException` - if object is the first object

---


### isEmpty

```
public boolean isEmpty()
```

:   Test if the priority queue is logically empty.

    :   **Returns:**: true if empty, false otherwise.

---


### size

```
public int size()
```

:   Returns size.

    :   **Returns:**: current size.

---


### makeEmpty

```
public void makeEmpty()
```

:   Make the priority queue logically empty.


---


|  |  |  |  |  |  |  |  |  |  |  |
| --- | --- | --- | --- | --- | --- | --- | --- | --- | --- | --- |
| |  |  |  |  |  |  |  | | --- | --- | --- | --- | --- | --- | --- | | Package | | **Class** | **Tree** | **Deprecated** | **Index** | **Help** | | | |  |
| **PREV CLASS**   **NEXT CLASS** | **FRAMES**    **NO FRAMES**     **All Classes** |
| SUMMARY: NESTED | FIELD | CONSTR | METHOD | DETAIL: FIELD | CONSTR | METHOD |


---
