# Supplementary material for: TABASCO: A single molecule, base-pair resolved gene expression simulator
Source: BMC Bioinformatics. 2007 Dec 19;8:480. doi: 10.1186/1471-2105-8-480 (PMC2242808; doi:10.1186/1471-2105-8-480)
Supplement: Additional File 3 — TABASCO website. [file 1471-2105-8-480-S3.zip › doc/Protein.html]

Protein


|  |  |  |  |  |  |  |  |  |  |  |
| --- | --- | --- | --- | --- | --- | --- | --- | --- | --- | --- |
| |  |  |  |  |  |  |  | | --- | --- | --- | --- | --- | --- | --- | | Package | | **Class** | **Tree** | **Deprecated** | **Index** | **Help** | | | |  |
| **PREV CLASS**   **NEXT CLASS** | **FRAMES**    **NO FRAMES**     **All Classes** |
| SUMMARY: NESTED | FIELD | CONSTR | METHOD | DETAIL: FIELD | CONSTR | METHOD |


---


## Class Protein

```
java.lang.Object
  Protein
```

**All Implemented Interfaces:**: Molecule

---

public class **Protein** extends java.lang.Object implements Molecule

A class for proteins that are contained in the cell.

**See Also:**: `Molecule`

---

|  |  |
| --- | --- |
| **Field Summary** | |
| `protected  int` | `copyNumber`             The number of copies of the protein |


|  |  |
| --- | --- |
| **Constructor Summary** | |
| `Protein(java.lang.String name, int copyNumber, int ID, int organism)` |


|  |  |
| --- | --- |
| **Method Summary** | |
| `int` | `getCopyNumber()`             Returns the number of copies of the protein |
| `int` | `getID()`             Returns the ID of the protein. |
| `java.lang.String` | `getName()`             Returns the name of the protein. |
| `int` | `getOrganism()`             Returns the organism of the protein. |
| `void` | `incrementCopyNumber(int inc)`             Incremements the copy number of the protein by inc. |

|  |
| --- |
| **Methods inherited from class java.lang.Object** |
| `clone, equals, finalize, getClass, hashCode, notify, notifyAll, toString, wait, wait, wait` |

|  |
| --- |
| **Field Detail** |

### copyNumber

```
protected int copyNumber
```

:   The number of copies of the protein


|  |
| --- |
| **Constructor Detail** |

### Protein

```
public Protein(java.lang.String name,
               int copyNumber,
               int ID,
               int organism)
```


|  |
| --- |
| **Method Detail** |

### getName

```
public java.lang.String getName()
```

:   Returns the name of the protein.

    :   **Returns:**: the name of the protein.

---


### getCopyNumber

```
public int getCopyNumber()
```

:   Returns the number of copies of the protein

    :   **Specified by:**: `getCopyNumber` in interface `Molecule`
    :   **Returns:**: the number of copies of the protein

---


### incrementCopyNumber

```
public void incrementCopyNumber(int inc)
```

:   Incremements the copy number of the protein by inc.

    :   **Specified by:**: `incrementCopyNumber` in interface `Molecule`
    :   **Parameters:**: `inc` - Increments the copy number of protein by inc. This can be negative or positive.

---


### getID

```
public int getID()
```

:   Returns the ID of the protein.

    :   **Specified by:**: `getID` in interface `Molecule`
    :   **Returns:**: the ID of the protein. This ID is indexed to the position in the myMolecules vector minus 1.

---


### getOrganism

```
public int getOrganism()
```

:   Returns the organism of the protein. Depracated.

    :   **Returns:**: the organism ID of the protein.


---


|  |  |  |  |  |  |  |  |  |  |  |
| --- | --- | --- | --- | --- | --- | --- | --- | --- | --- | --- |
| |  |  |  |  |  |  |  | | --- | --- | --- | --- | --- | --- | --- | | Package | | **Class** | **Tree** | **Deprecated** | **Index** | **Help** | | | |  |
| **PREV CLASS**   **NEXT CLASS** | **FRAMES**    **NO FRAMES**     **All Classes** |
| SUMMARY: NESTED | FIELD | CONSTR | METHOD | DETAIL: FIELD | CONSTR | METHOD |


---
