# Supplementary material for: TABASCO: A single molecule, base-pair resolved gene expression simulator
Source: BMC Bioinformatics. 2007 Dec 19;8:480. doi: 10.1186/1471-2105-8-480 (PMC2242808; doi:10.1186/1471-2105-8-480)
Supplement: Additional File 3 — TABASCO website. [file 1471-2105-8-480-S3.zip › doc/Reaction.html]

Reaction


|  |  |  |  |  |  |  |  |  |  |  |
| --- | --- | --- | --- | --- | --- | --- | --- | --- | --- | --- |
| |  |  |  |  |  |  |  | | --- | --- | --- | --- | --- | --- | --- | | Package | | **Class** | **Tree** | **Deprecated** | **Index** | **Help** | | | |  |
| **PREV CLASS**   **NEXT CLASS** | **FRAMES**    **NO FRAMES**     **All Classes** |
| SUMMARY: NESTED | FIELD | CONSTR | METHOD | DETAIL: FIELD | CONSTR | METHOD |


---


## Class Reaction

```
java.lang.Object
  Reaction
```

---

public class **Reaction** extends java.lang.Object

A general class for Reactions. Most instantiations of Reaction actually instantiate from subclasses. This class should perhaps become abstract.

---

|  |  |
| --- | --- |
| **Field Summary** | |
| `protected  int` | `ID`             The reactions ID. |


|  |  |
| --- | --- |
| **Constructor Summary** | |
| `protected` | `Reaction(int ID, double rc)`             A default empty constructor. |
|  | `Reaction(int ID, Molecule[] reac, Molecule[] prod, double rc)`             A constructor that takes in reactant and product molecules as well. |


|  |  |
| --- | --- |
| **Method Summary** | |
| `Molecule[]` | `affects()`             Returns all the molecules that are affected upon the execution of this reaction. |
| `void` | `calculateA()`             Calculate the propensity of the reaction. |
| `void` | `calculateTime(double random, double t)`             Calculate the time the reaction will next execute. |
| `double` | `compareTo(Reaction rhs)`             Compares this reaction time to execute to another reaction. |
| `Molecule[]` | `dependsOn()`             Returns the set of reactants that this reaction depends on. |
| `void` | `execute()`             Executes the reaction. |
| `void` | `findAffects(Reaction r)`             A method that is used upon initialization to populate the updateRxn vector. |
| `double` | `getA()`             Returns the current propensity of the reaction. |
| `int` | `getID()`             Returns the ID of the reaction. |
| `double` | `getTime()`             Returns the time that this reaction is next expected to execute. |
| `void` | `setTime(double t)`             Sets the time that this reaction is next expected to execute. |

|  |
| --- |
| **Methods inherited from class java.lang.Object** |
| `clone, equals, finalize, getClass, hashCode, notify, notifyAll, toString, wait, wait, wait` |

|  |
| --- |
| **Field Detail** |

### ID

```
protected int ID
```

:   The reactions ID. Relates to the indices in the IndexedPriorityQueue.


|  |
| --- |
| **Constructor Detail** |

### Reaction

```
protected Reaction(int ID,
                   double rc)
```

:   A default empty constructor. Assumes very little about the number of reactants and products.

    **Parameters:**: `ID` - the reaction ID: `rc` - the rate constant of the reaction.

---


### Reaction

```
public Reaction(int ID,
                Molecule[] reac,
                Molecule[] prod,
                double rc)
```

:   A constructor that takes in reactant and product molecules as well.

    **Parameters:**: `ID` - the reaction ID: `reac` - The reactants to be decremented upon execution of this reaction.: `prod` - The products to be incremented upon execution of this reaction.: `rc` - The mesoscopic rate constant of the reaction.


|  |
| --- |
| **Method Detail** |

### getID

```
public int getID()
```

:   Returns the ID of the reaction. This is linked to the address in Gintegrator.

    :   **Returns:**: the ID of the reaction.

---


### getTime

```
public double getTime()
```

:   Returns the time that this reaction is next expected to execute.

    :   **Returns:**: the time that this reaction is next expected to execute.

---


### setTime

```
public void setTime(double t)
```

:   Sets the time that this reaction is next expected to execute.

    :   **Parameters:**: `t` - The time that this reaction is next expected to execute.

---


### getA

```
public double getA()
```

:   Returns the current propensity of the reaction. The propensity, a, is usually defined as the mesoscopic rate constant times the number of molecules of each reactant.

    :   **Returns:**: the current propensity of the reaction.

---


### compareTo

```
public double compareTo(Reaction rhs)
```

:   Compares this reaction time to execute to another reaction. It subtracts rhs.time from the current time of this reaction. This is useful for ordering in the IndexedPriorityQueue.

    :   **Parameters:**: `rhs` - The reaction to compare time to next execution with.

---


### calculateA

```
public void calculateA()
```

:   Calculate the propensity of the reaction. This is accomplished here as the mesoscopic rate constant times the number of molecules of each reactant.

---


### calculateTime

```
public void calculateTime(double random,
                          double t)
```

:   Calculate the time the reaction will next execute. Assumes an exponential distribution, witht he propensity being the average.

    :   **Parameters:**: `random` - A randomly generated double value between [0,1) that will be used to choose a time based on the distribution.: `t` - The current time of the simulation.

---


### execute

```
public void execute()
```

:   Executes the reaction. Increments the copy numbers of the reactants and decrements the copy numbers of the products.

---


### dependsOn

```
public Molecule[] dependsOn()
```

:   Returns the set of reactants that this reaction depends on. In this case, just the reactants.

    :   **Returns:**: an array of reactant molecules that this reaction depends on.

---


### affects

```
public Molecule[] affects()
```

:   Returns all the molecules that are affected upon the execution of this reaction. In this case, all the reactants and products.

    :   **Returns:**: an array of reactant molecules that this reaction affects.

---


### findAffects

```
public void findAffects(Reaction r)
```

:   A method that is used upon initialization to populate the updateRxn vector. Checks if this reaction affects a particular reaction R (in which case this the particular
    reaction will be added to the reaction's updateRxn vector) and if that particular reaction R affects this reaction (in which case the particular reactions updateRxn vector will be appended).


---


|  |  |  |  |  |  |  |  |  |  |  |
| --- | --- | --- | --- | --- | --- | --- | --- | --- | --- | --- |
| |  |  |  |  |  |  |  | | --- | --- | --- | --- | --- | --- | --- | | Package | | **Class** | **Tree** | **Deprecated** | **Index** | **Help** | | | |  |
| **PREV CLASS**   **NEXT CLASS** | **FRAMES**    **NO FRAMES**     **All Classes** |
| SUMMARY: NESTED | FIELD | CONSTR | METHOD | DETAIL: FIELD | CONSTR | METHOD |


---
