# Supplementary material for: TABASCO: A single molecule, base-pair resolved gene expression simulator
Source: BMC Bioinformatics. 2007 Dec 19;8:480. doi: 10.1186/1471-2105-8-480 (PMC2242808; doi:10.1186/1471-2105-8-480)
Supplement: Additional File 3 — TABASCO website. [file 1471-2105-8-480-S3.zip › doc/RNA.html]

RNA


|  |  |  |  |  |  |  |  |  |  |  |
| --- | --- | --- | --- | --- | --- | --- | --- | --- | --- | --- |
| |  |  |  |  |  |  |  | | --- | --- | --- | --- | --- | --- | --- | | Package | | **Class** | **Tree** | **Deprecated** | **Index** | **Help** | | | |  |
| **PREV CLASS**   **NEXT CLASS** | **FRAMES**    **NO FRAMES**     **All Classes** |
| SUMMARY: NESTED | FIELD | CONSTR | METHOD | DETAIL: FIELD | CONSTR | METHOD |


---


## Class RNA

```
java.lang.Object
  RNA
```

**All Implemented Interfaces:**: Molecule

---

public class **RNA** extends java.lang.Object implements Molecule

General class for RNA molecules.

**See Also:**: `TabascoSimulator`

---

|  |  |
| --- | --- |
| **Constructor Summary** | |
| `RNA(int ID)`             A constructor for an RNA molecule |
| `RNA(int start, int stop)`             A constructor for an RNA molecule |
| `RNA(int start, int stop, int ID)`             A constructor for an RNA molecule |


|  |  |
| --- | --- |
| **Method Summary** | |
| `int` | `getCopyNumber()`             Returns the current copy number of the RNA |
| `int` | `getID()`             Returns the ID of the RNA. |
| `int` | `getLength()`             Returns the current length of the RNA. |
| `int` | `getStart()`             Returns the most upstream position of the RNA in the DNA coordinates from where it is transcribed. |
| `int` | `getStop()`             Returns the most dowsntream position of the RNA in the DNA coordinates from where it is transcribed. |
| `void` | `incrementCopyNumber(int inc)`             Increments the number of copies of RNA by inc. |

|  |
| --- |
| **Methods inherited from class java.lang.Object** |
| `clone, equals, finalize, getClass, hashCode, notify, notifyAll, toString, wait, wait, wait` |

|  |
| --- |
| **Constructor Detail** |

### RNA

```
public RNA(int start,
           int stop)
```

:   A constructor for an RNA molecule

    **Parameters:**: `start` - The most upstream position of the RNA in the DNA coordinates from where it is transcribed.: `stop` - The most downstream position of the RNA in the DNA coordinates from where it is transcribed.

---


### RNA

```
public RNA(int start,
           int stop,
           int ID)
```

:   A constructor for an RNA molecule

    **Parameters:**: `start` - The most upstream position of the RNA in the DNA coordinates from where it is transcribed.: `stop` - The most downstream position of the RNA in the DNA coordinates from where it is transcribed.: `ID` - the ID of the RNA molecule

---


### RNA

```
public RNA(int ID)
```

:   A constructor for an RNA molecule

    **Parameters:**: `ID` - the ID of the RNA molecule


|  |
| --- |
| **Method Detail** |

### getStart

```
public int getStart()
```

:   Returns the most upstream position of the RNA in the DNA coordinates from where it is transcribed.

    :   **Returns:**: the most upstream position of the RNA in the DNA coordinates from where it is transcribed.

---


### incrementCopyNumber

```
public void incrementCopyNumber(int inc)
```

:   Increments the number of copies of RNA by inc.

    :   **Specified by:**: `incrementCopyNumber` in interface `Molecule`
    :   **Parameters:**: `inc` - The number of copies of RNA to increment the current copy number by.

---


### getCopyNumber

```
public int getCopyNumber()
```

:   Returns the current copy number of the RNA

    :   **Specified by:**: `getCopyNumber` in interface `Molecule`
    :   **Returns:**: the current copy number of the RNA.

---


### getStop

```
public int getStop()
```

:   Returns the most dowsntream position of the RNA in the DNA coordinates from where it is transcribed.

    :   **Returns:**: the most downstream position of the RNA in the DNA coordinates from where it is transcribed.

---


### getLength

```
public int getLength()
```

:   Returns the current length of the RNA. Simply returns (stop-start+1).

---


### getID

```
public int getID()
```

:   Returns the ID of the RNA.

    :   **Specified by:**: `getID` in interface `Molecule`
    :   **Returns:**: the ID of the RNA.


---


|  |  |  |  |  |  |  |  |  |  |  |
| --- | --- | --- | --- | --- | --- | --- | --- | --- | --- | --- |
| |  |  |  |  |  |  |  | | --- | --- | --- | --- | --- | --- | --- | | Package | | **Class** | **Tree** | **Deprecated** | **Index** | **Help** | | | |  |
| **PREV CLASS**   **NEXT CLASS** | **FRAMES**    **NO FRAMES**     **All Classes** |
| SUMMARY: NESTED | FIELD | CONSTR | METHOD | DETAIL: FIELD | CONSTR | METHOD |


---
