# Supplementary material for: TABASCO: A single molecule, base-pair resolved gene expression simulator
Source: BMC Bioinformatics. 2007 Dec 19;8:480. doi: 10.1186/1471-2105-8-480 (PMC2242808; doi:10.1186/1471-2105-8-480)
Supplement: Additional File 3 — TABASCO website. [file 1471-2105-8-480-S3.zip › doc/TabascoDraw.html]

TabascoDraw


|  |  |  |  |  |  |  |  |  |  |  |
| --- | --- | --- | --- | --- | --- | --- | --- | --- | --- | --- |
| |  |  |  |  |  |  |  | | --- | --- | --- | --- | --- | --- | --- | | Package | | **Class** | **Tree** | **Deprecated** | **Index** | **Help** | | | |  |
| **PREV CLASS**   **NEXT CLASS** | **FRAMES**    **NO FRAMES**     **All Classes** |
| SUMMARY: NESTED | FIELD | CONSTR | METHOD | DETAIL: FIELD | CONSTR | METHOD |


---


## Class TabascoDraw

```
java.lang.Object
  TabascoDraw
```

---

public class **TabascoDraw** extends java.lang.Object

Tabasco Draw is part of the visualization suite for the Tabasco stochastic simulator.
\* Note: should consider making a single method to be implemented by the makeGraphic() and makeSingleGraphic() methods,
\* since they are essentially identical. Problem is my lack of understanding of combining Images. Soemthing for later.
\*
\* Should also consider adding a check for the ScreenHeight, since the program blows up if it gets too big (Aside from it being
\* just non-user friendly with some super small scroll bar) Instead could break it up into a few windows, something to
\* consider adding somewhere, maybe in the TabascoReader.java file, it could break a large file up into pieces.
\* @author Jason Kelly
\* @author Sriram Kosuri
\* @version 1.0

---

|  |  |
| --- | --- |
| **Constructor Summary** | |
| `TabascoDraw(TabascoRead tRead)`             The only constructor for this class. |


|  |  |
| --- | --- |
| **Method Summary** | |
| `static java.awt.Graphics2D` | `makeDNAGraphic(java.awt.Graphics2D g, java.lang.String[] complexArray, int enteredDNA, int startX, int startY, int BP_PER_PIXEL, int POLYMERASE_HEIGHT, int PROMOTER_HEIGHT, int TERMINATOR_HEIGHT, int NORMAL_DNA_HEIGHT, int NUM_BETWEEN_TRACERS, int ENTERED_DNA_WIDTH, int ENTERED_DNA_HEIGHT, int[] dnaArray)`             This method takes a Graphics2D object and adds a representation of the DNA with complexes and genetic elements annotated onto it. |
| `java.awt.image.BufferedImage` | `makeMoleculeGraph(java.lang.String[] molNames, int[] molCopyNumber, java.awt.Color[] molColors, double time, int molGraphHeight, int Y_MARGIN, int[] rcopynumber, java.awt.image.BufferedImage myImage)`             This method takes in an image, and returns one with a molecule graph added. |
| `static java.awt.Graphics2D` | `makeMRNAGraphic(java.awt.Graphics2D theG, int[] rStart, int[] rLength, int[] rCopyNumber, int startX, int startY, java.awt.Color[] colorArray, int heightScale, int bpPerPixel)`             This method takes in a Graphics2D object and draws an image depicting the nascentRNA levels of all the coding domains on the DNA. |
| `static java.awt.image.BufferedImage` | `makeSingleGraphic(java.lang.String[] ca, int[] rstart, int[] rlength, int[] rcopynumber, int entDNA, int rowNum, int totRows, java.awt.image.BufferedImage myImage, int[] dnaArray)`             This is the method that sets the characteristics of the DNA visualization, and runs the other methods in this class within it. |

|  |
| --- |
| **Methods inherited from class java.lang.Object** |
| `clone, equals, finalize, getClass, hashCode, notify, notifyAll, toString, wait, wait, wait` |

|  |
| --- |
| **Constructor Detail** |

### TabascoDraw

```
public TabascoDraw(TabascoRead tRead)
```

:   The only constructor for this class.
    \* @param tRead The instance of TabascoRead that is reading the output of a simulation to be visualized


|  |
| --- |
| **Method Detail** |

### makeMoleculeGraph

```
public java.awt.image.BufferedImage makeMoleculeGraph(java.lang.String[] molNames,
                                                      int[] molCopyNumber,
                                                      java.awt.Color[] molColors,
                                                      double time,
                                                      int molGraphHeight,
                                                      int Y_MARGIN,
                                                      int[] rcopynumber,
                                                      java.awt.image.BufferedImage myImage)
```

:   This method takes in an image, and returns one with a molecule graph added. This graph shows the levels of all the proteins and mRNA species on a log plot.
    \* @param molNames A vector containing the names of the molecules to be listed
    \* @param molCopyNumber A vector that corresponds to the molNames containing the copy number of the particular molecule
    \* @param molColors A vector containing a set of colors to iterate through when visualizing the protein levels
    \* @param time The time to be displayed for this particular image
    \* @param molGraphHeight The height of the molecule graph should take up in the original image
    \* @return A new BufferedImage with a molecule graph replacing the lower portion of the image.

---


### makeMRNAGraphic

```
public static java.awt.Graphics2D makeMRNAGraphic(java.awt.Graphics2D theG,
                                                  int[] rStart,
                                                  int[] rLength,
                                                  int[] rCopyNumber,
                                                  int startX,
                                                  int startY,
                                                  java.awt.Color[] colorArray,
                                                  int heightScale,
                                                  int bpPerPixel)
```

:   This method takes in a Graphics2D object and draws an image depicting the nascentRNA levels of all the coding domains on the DNA.
    \* @param theG The graphics2D object to edit from
    \* @param rStart An array containing the startsites in base pairs of the coding domains on the DNA
    \* @param rLength An array containing the lengths in base pairs of the coding domains on the DNA. The indices correspond to those in rStart
    \* @param rCopyNumber An array containing the copy numbers of the coding domains on the DNA. THe indices correspond to those in rStart
    \* @param startX The x position in the Graphics2D context where the drawing of the genome begins
    \* @param startY The y position in the Graphics2D context where the drawing of the genome begins
    \* @param colorArray The set of colors to use for the polymerasess and proteins displayed on the DNA
    \* @param heightScale A scaling factor that is used to scale different sized simulations
    \* @param bpPerPixel The number of base pairs that are representative of a single pixel.
    \* @returns The updated Graphics2d containing the visualization of the nascentRNA.

---


### makeSingleGraphic

```
public static java.awt.image.BufferedImage makeSingleGraphic(java.lang.String[] ca,
                                                             int[] rstart,
                                                             int[] rlength,
                                                             int[] rcopynumber,
                                                             int entDNA,
                                                             int rowNum,
                                                             int totRows,
                                                             java.awt.image.BufferedImage myImage,
                                                             int[] dnaArray)
```

:   This is the method that sets the characteristics of the DNA visualization, and runs the other methods in this class within it.
    \* @param ca An array containing a string of numbers that defines the type and instance of complexes on the dna.
    \* @param rstart An array containing the startsites in base pairs of the coding domains on the DNA
    \* @param rlength An array containing the lengths in base pairs of the coding domains on the DNA. The indices correspond to those in rStart
    \* @param rcopynumber An array containing the copy numbers of the coding domains on the DNA. THe indices correspond to those in rStart
    \* @param entDNA The last position on the genome that has entered the cell
    \* @param rowNum The current DNA molecules to display; Sometimes mutiple pieces of DNA will be shown on different rows for the same simulation
    \* @param totRows The total number of DNA molecules to display.
    \* @param myImage The image to draw the DNA on
    \* @param dnaArray An array describing the elements on the DNA at each DNA position.

---


### makeDNAGraphic

```
public static java.awt.Graphics2D makeDNAGraphic(java.awt.Graphics2D g,
                                                 java.lang.String[] complexArray,
                                                 int enteredDNA,
                                                 int startX,
                                                 int startY,
                                                 int BP_PER_PIXEL,
                                                 int POLYMERASE_HEIGHT,
                                                 int PROMOTER_HEIGHT,
                                                 int TERMINATOR_HEIGHT,
                                                 int NORMAL_DNA_HEIGHT,
                                                 int NUM_BETWEEN_TRACERS,
                                                 int ENTERED_DNA_WIDTH,
                                                 int ENTERED_DNA_HEIGHT,
                                                 int[] dnaArray)
```

:   This method takes a Graphics2D object and adds a representation of the DNA with complexes and genetic elements annotated onto it.
    \* @param g The Graphics2D object that the representation will be added to.
    \* @param complexArray The Graphics2D object that
    \* @param enteredDNA
    \* @param startX
    \* @param startY
    \* @param BP\_PER\_PIXEL
    \* @param POLYMERASE\_HEIGHT
    \* @param PROMOTER\_HEIGHT
    \* @param TERMINATOR\_HEIGHT
    \* @param NORMAL\_DNA\_HEIGHT
    \* @param NUM\_BETWEEN\_TRACERS
    \* @param ENTERED\_DNA\_WIDTH
    \* @param ENTERED\_DNA\_HEIGHT
    \* @param dnaArray


---


|  |  |  |  |  |  |  |  |  |  |  |
| --- | --- | --- | --- | --- | --- | --- | --- | --- | --- | --- |
| |  |  |  |  |  |  |  | | --- | --- | --- | --- | --- | --- | --- | | Package | | **Class** | **Tree** | **Deprecated** | **Index** | **Help** | | | |  |
| **PREV CLASS**   **NEXT CLASS** | **FRAMES**    **NO FRAMES**     **All Classes** |
| SUMMARY: NESTED | FIELD | CONSTR | METHOD | DETAIL: FIELD | CONSTR | METHOD |


---
