# Supplementary material for: TABASCO: A single molecule, base-pair resolved gene expression simulator
Source: BMC Bioinformatics. 2007 Dec 19;8:480. doi: 10.1186/1471-2105-8-480 (PMC2242808; doi:10.1186/1471-2105-8-480)
Supplement: Additional File 3 — TABASCO website. [file 1471-2105-8-480-S3.zip › doc/TabascoJpegMake.html]

TabascoJpegMake


|  |  |  |  |  |  |  |  |  |  |  |
| --- | --- | --- | --- | --- | --- | --- | --- | --- | --- | --- |
| |  |  |  |  |  |  |  | | --- | --- | --- | --- | --- | --- | --- | | Package | | **Class** | **Tree** | **Deprecated** | **Index** | **Help** | | | |  |
| **PREV CLASS**   **NEXT CLASS** | **FRAMES**    **NO FRAMES**     **All Classes** |
| SUMMARY: NESTED | FIELD | CONSTR | METHOD | DETAIL: FIELD | CONSTR | METHOD |


---


## Class TabascoJpegMake

```
java.lang.Object
  TabascoJpegMake
```

---

public class **TabascoJpegMake** extends java.lang.Object

TabascoJpegMake is class to make visualization of the output by making a stack of images of individual timepoints. One can compile these images into a movie using standard programs (ie, QuickTime)
\*
\* Currently this class is optimized to output an image that fits the 40,000 bp genome of T7 and its encoded genes. The code would have to be recompiled to fit other types of genomes.
\*
\* @author Sriram Kosuri
\* @author Jason Kelly
\* @version 1.0

---

|  |  |
| --- | --- |
| **Constructor Summary** | |
| `TabascoJpegMake()` |


|  |  |
| --- | --- |
| **Method Summary** | |
| `static void` | `main(java.lang.String[] args)`             The class that is called when running this class from the command-line. |
| `static void` | `writeFiles(java.lang.String[] phageInputFiles, java.lang.String molFileName, java.lang.String jpegOutputFile)`             The main method used to write files. |

|  |
| --- |
| **Methods inherited from class java.lang.Object** |
| `clone, equals, finalize, getClass, hashCode, notify, notifyAll, toString, wait, wait, wait` |

|  |
| --- |
| **Constructor Detail** |

### TabascoJpegMake

```
public TabascoJpegMake()
```


|  |
| --- |
| **Method Detail** |

### main

```
public static void main(java.lang.String[] args)
```

:   The class that is called when running this class from the command-line.
    \* @param args the arguments that are supposed to be passed is the output file prefix, the input file that ran the simulation, and the individual DNA files that are to be visualized

---


### writeFiles

```
public static void writeFiles(java.lang.String[] phageInputFiles,
                              java.lang.String molFileName,
                              java.lang.String jpegOutputFile)
```

:   The main method used to write files.
    \* @param phageInputFiles A string array with locations of the phage DNA files to open
    \* @param molFileName A string with the location of the molecule output file from the simulation
    \* @param jpegoutputFile A string with the prefix for the output files.


---


|  |  |  |  |  |  |  |  |  |  |  |
| --- | --- | --- | --- | --- | --- | --- | --- | --- | --- | --- |
| |  |  |  |  |  |  |  | | --- | --- | --- | --- | --- | --- | --- | | Package | | **Class** | **Tree** | **Deprecated** | **Index** | **Help** | | | |  |
| **PREV CLASS**   **NEXT CLASS** | **FRAMES**    **NO FRAMES**     **All Classes** |
| SUMMARY: NESTED | FIELD | CONSTR | METHOD | DETAIL: FIELD | CONSTR | METHOD |


---
