# Supplementary material for: TABASCO: A single molecule, base-pair resolved gene expression simulator
Source: BMC Bioinformatics. 2007 Dec 19;8:480. doi: 10.1186/1471-2105-8-480 (PMC2242808; doi:10.1186/1471-2105-8-480)
Supplement: Additional File 3 — TABASCO website. [file 1471-2105-8-480-S3.zip › doc/TabascoRead.html]

TabascoRead


|  |  |  |  |  |  |  |  |  |  |  |
| --- | --- | --- | --- | --- | --- | --- | --- | --- | --- | --- |
| |  |  |  |  |  |  |  | | --- | --- | --- | --- | --- | --- | --- | | Package | | **Class** | **Tree** | **Deprecated** | **Index** | **Help** | | | |  |
| **PREV CLASS**   **NEXT CLASS** | **FRAMES**    **NO FRAMES**     **All Classes** |
| SUMMARY: NESTED | FIELD | CONSTR | METHOD | DETAIL: FIELD | CONSTR | METHOD |


---


## Class TabascoRead

```
java.lang.Object
  TabascoRead
```

---

public class **TabascoRead** extends java.lang.Object

TabascoRead is the class that is used to read DNA output files of simulations.

---

|  |  |
| --- | --- |
| **Constructor Summary** | |
| `TabascoRead(java.lang.String filename)` |


|  |  |
| --- | --- |
| **Method Summary** | |
| `void` | `cleanUp()` |
| `void` | `clearMemFast()` |
| `java.lang.String[]` | `getComplexArrayFast()` |
| `int[]` | `getDNAArray()` |
| `int` | `getEnteredDNA(int stepNumber)` |
| `int` | `getEnteredDNAFast()` |
| `int` | `getGenomeSize()` |
| `int` | `getMRNACopyNumber(int stepNumber, int codingRgn)` |
| `int[]` | `getMRNACopyNumberFast()` |
| `int` | `getMRNALength(int stepNumber, int codingRgn)` |
| `int[]` | `getMRNALengthFast()` |
| `int` | `getMRNAStart(int stepNumber, int codingRgn)` |
| `int[]` | `getMRNAStartFast()` |
| `int` | `getNumberOfCodingRgns()` |
| `double` | `getTime(int stepNumber)` |
| `int` | `getTimeEnd()` |
| `double` | `getTimeFast()` |
| `int` | `getTimePoints()` |
| `int` | `getTimeStep()` |
| `boolean` | `IsThereNextComplexArray()` |
| `void` | `loadNextDNAArrayFast()` |
| `java.io.BufferedReader` | `resetBr(java.io.BufferedReader brToBeReset, java.lang.String filename)` |
| `java.util.StringTokenizer` | `returnTokenRow(int rowNum)` |

|  |
| --- |
| **Methods inherited from class java.lang.Object** |
| `clone, equals, finalize, getClass, hashCode, notify, notifyAll, toString, wait, wait, wait` |

|  |
| --- |
| **Constructor Detail** |

### TabascoRead

```
public TabascoRead(java.lang.String filename)
            throws java.io.IOException
```


|  |
| --- |
| **Method Detail** |

### loadNextDNAArrayFast

```
public void loadNextDNAArrayFast()
```

---


### clearMemFast

```
public void clearMemFast()
```

---


### getTimeFast

```
public double getTimeFast()
```

---


### getEnteredDNAFast

```
public int getEnteredDNAFast()
```

---


### getComplexArrayFast

```
public java.lang.String[] getComplexArrayFast()
```

---


### getMRNAStartFast

```
public int[] getMRNAStartFast()
```

---


### getMRNALengthFast

```
public int[] getMRNALengthFast()
```

---


### getMRNACopyNumberFast

```
public int[] getMRNACopyNumberFast()
```

---


### getTime

```
public double getTime(int stepNumber)
               throws java.io.IOException
```

:   **Throws:**: `java.io.IOException`

---


### getEnteredDNA

```
public int getEnteredDNA(int stepNumber)
                  throws java.io.IOException
```

:   **Throws:**: `java.io.IOException`

---


### getMRNAStart

```
public int getMRNAStart(int stepNumber,
                        int codingRgn)
                 throws java.io.IOException
```

:   **Throws:**: `java.io.IOException`

---


### getMRNALength

```
public int getMRNALength(int stepNumber,
                         int codingRgn)
                  throws java.io.IOException
```

:   **Throws:**: `java.io.IOException`

---


### getMRNACopyNumber

```
public int getMRNACopyNumber(int stepNumber,
                             int codingRgn)
                      throws java.io.IOException
```

:   **Throws:**: `java.io.IOException`

---


### getTimeStep

```
public int getTimeStep()
```

---


### getNumberOfCodingRgns

```
public int getNumberOfCodingRgns()
```

---


### getTimeEnd

```
public int getTimeEnd()
```

---


### getTimePoints

```
public int getTimePoints()
```

---


### getGenomeSize

```
public int getGenomeSize()
```

---


### getDNAArray

```
public int[] getDNAArray()
```

---


### returnTokenRow

```
public java.util.StringTokenizer returnTokenRow(int rowNum)
                                         throws java.io.IOException
```

:   **Throws:**: `java.io.IOException`

---


### resetBr

```
public java.io.BufferedReader resetBr(java.io.BufferedReader brToBeReset,
                                      java.lang.String filename)
                               throws java.io.IOException
```

:   **Throws:**: `java.io.IOException`

---


### cleanUp

```
public void cleanUp()
```

---


### IsThereNextComplexArray

```
public boolean IsThereNextComplexArray()
```


---


|  |  |  |  |  |  |  |  |  |  |  |
| --- | --- | --- | --- | --- | --- | --- | --- | --- | --- | --- |
| |  |  |  |  |  |  |  | | --- | --- | --- | --- | --- | --- | --- | | Package | | **Class** | **Tree** | **Deprecated** | **Index** | **Help** | | | |  |
| **PREV CLASS**   **NEXT CLASS** | **FRAMES**    **NO FRAMES**     **All Classes** |
| SUMMARY: NESTED | FIELD | CONSTR | METHOD | DETAIL: FIELD | CONSTR | METHOD |


---
