# Supplementary material for: TABASCO: A single molecule, base-pair resolved gene expression simulator
Source: BMC Bioinformatics. 2007 Dec 19;8:480. doi: 10.1186/1471-2105-8-480 (PMC2242808; doi:10.1186/1471-2105-8-480)
Supplement: Additional File 3 — TABASCO website. [file 1471-2105-8-480-S3.zip › doc/TabascoReadMol.html]

TabascoReadMol


|  |  |  |  |  |  |  |  |  |  |  |
| --- | --- | --- | --- | --- | --- | --- | --- | --- | --- | --- |
| |  |  |  |  |  |  |  | | --- | --- | --- | --- | --- | --- | --- | | Package | | **Class** | **Tree** | **Deprecated** | **Index** | **Help** | | | |  |
| **PREV CLASS**   **NEXT CLASS** | **FRAMES**    **NO FRAMES**     **All Classes** |
| SUMMARY: NESTED | FIELD | CONSTR | METHOD | DETAIL: FIELD | CONSTR | METHOD |


---


## Class TabascoReadMol

```
java.lang.Object
  TabascoReadMol
```

---

public class **TabascoReadMol** extends java.lang.Object

TabascoReadMol is the class that is used to read molecule output files of simulations.

---

|  |  |
| --- | --- |
| **Constructor Summary** | |
| `TabascoReadMol(java.lang.String MolFileLoc)` |


|  |  |
| --- | --- |
| **Method Summary** | |
| `void` | `close()` |
| `int[]` | `getCustomProteinCopyNumbers()` |
| `java.lang.String[]` | `getCustomProtNames()` |
| `int` | `getNumberOfColiPolymerase()` |
| `int` | `getNumberOfRNA()` |
| `int` | `getNumberOfT7Polymerase()` |
| `int` | `getNumOfTimePoints()` |
| `int[]` | `getPolymeraseCopyNumbers()` |
| `java.lang.String[]` | `getPolymeraseNames()` |
| `int` | `getRibosomeCopyNumber()` |
| `int[]` | `getRNACopyNumbers()` |
| `java.lang.String[]` | `getRNANames()` |
| `double` | `getTime()` |
| `void` | `loadNextMoleculeArrayFast()` |
| `static void` | `main(java.lang.String[] args)` |

|  |
| --- |
| **Methods inherited from class java.lang.Object** |
| `clone, equals, finalize, getClass, hashCode, notify, notifyAll, toString, wait, wait, wait` |

|  |
| --- |
| **Constructor Detail** |

### TabascoReadMol

```
public TabascoReadMol(java.lang.String MolFileLoc)
```


|  |
| --- |
| **Method Detail** |

### close

```
public void close()
           throws java.io.IOException
```

:   **Throws:**: `java.io.IOException`

---


### loadNextMoleculeArrayFast

```
public void loadNextMoleculeArrayFast()
```

---


### getTime

```
public double getTime()
```

---


### getPolymeraseCopyNumbers

```
public int[] getPolymeraseCopyNumbers()
```

---


### getRibosomeCopyNumber

```
public int getRibosomeCopyNumber()
```

---


### getCustomProteinCopyNumbers

```
public int[] getCustomProteinCopyNumbers()
```

---


### getNumberOfColiPolymerase

```
public int getNumberOfColiPolymerase()
```

---


### getNumberOfT7Polymerase

```
public int getNumberOfT7Polymerase()
```

---


### getNumberOfRNA

```
public int getNumberOfRNA()
```

---


### getRNACopyNumbers

```
public int[] getRNACopyNumbers()
```

---


### getRNANames

```
public java.lang.String[] getRNANames()
```

---


### getPolymeraseNames

```
public java.lang.String[] getPolymeraseNames()
```

---


### getCustomProtNames

```
public java.lang.String[] getCustomProtNames()
```

---


### getNumOfTimePoints

```
public int getNumOfTimePoints()
```

---


### main

```
public static void main(java.lang.String[] args)
```


---


|  |  |  |  |  |  |  |  |  |  |  |
| --- | --- | --- | --- | --- | --- | --- | --- | --- | --- | --- |
| |  |  |  |  |  |  |  | | --- | --- | --- | --- | --- | --- | --- | | Package | | **Class** | **Tree** | **Deprecated** | **Index** | **Help** | | | |  |
| **PREV CLASS**   **NEXT CLASS** | **FRAMES**    **NO FRAMES**     **All Classes** |
| SUMMARY: NESTED | FIELD | CONSTR | METHOD | DETAIL: FIELD | CONSTR | METHOD |


---
