# Supplementary material for: TABASCO: A single molecule, base-pair resolved gene expression simulator
Source: BMC Bioinformatics. 2007 Dec 19;8:480. doi: 10.1186/1471-2105-8-480 (PMC2242808; doi:10.1186/1471-2105-8-480)
Supplement: Additional File 3 — TABASCO website. [file 1471-2105-8-480-S3.zip › doc/TabascoSimulator.html]

TabascoSimulator


|  |  |  |  |  |  |  |  |  |  |  |
| --- | --- | --- | --- | --- | --- | --- | --- | --- | --- | --- |
| |  |  |  |  |  |  |  | | --- | --- | --- | --- | --- | --- | --- | | Package | | **Class** | **Tree** | **Deprecated** | **Index** | **Help** | | | |  |
| **PREV CLASS**   **NEXT CLASS** | **FRAMES**    **NO FRAMES**     **All Classes** |
| SUMMARY: NESTED | FIELD | CONSTR | METHOD | DETAIL: FIELD | CONSTR | METHOD |


---


## Class TabascoSimulator

```
java.lang.Object
  TabascoSimulator
```

---

public class **TabascoSimulator** extends java.lang.Object

TabascoSimulator is the class used to run simulations and produce output files.

---

|  |  |
| --- | --- |
| **Constructor Summary** | |
| `TabascoSimulator()` |


|  |  |
| --- | --- |
| **Method Summary** | |
| `static int` | `combineData(int iterations, java.lang.String OUTPUT_FILE_LOC, java.lang.String STD_FILE_LOC, java.lang.String AVG_FILE_LOC)`             Averages the molecule file data across multiple simulations |
| `static void` | `combineEnergyData(int iterations, java.lang.String OUTPUT_FILE_LOC, java.lang.String STD_FILE_LOC, java.lang.String AVG_FILE_LOC, int num_of_time_points)`             Averages the energy output files from individual simulations. |
| `static void` | `combineInitRnaData(int iterations, java.lang.String OUTPUT_FILE_LOC, java.lang.String STD_FILE_LOC, java.lang.String AVG_FILE_LOC, int num_of_time_points)`             Averages the RNA initiation output files from individual simulations. |
| `static void` | `main(java.lang.String[] args)`             The method run from the command line to run Tabasco Simulations. |

|  |
| --- |
| **Methods inherited from class java.lang.Object** |
| `clone, equals, finalize, getClass, hashCode, notify, notifyAll, toString, wait, wait, wait` |

|  |
| --- |
| **Constructor Detail** |

### TabascoSimulator

```
public TabascoSimulator()
```


|  |
| --- |
| **Method Detail** |

### main

```
public static void main(java.lang.String[] args)
```

:   The method run from the command line to run Tabasco Simulations.

    :   **Parameters:**: `args` - The arguments passed to the simulator from the command line. The common usage is the input file, the output file prefix, and an optional random seed.

---


### combineEnergyData

```
public static void combineEnergyData(int iterations,
                                     java.lang.String OUTPUT_FILE_LOC,
                                     java.lang.String STD_FILE_LOC,
                                     java.lang.String AVG_FILE_LOC,
                                     int num_of_time_points)
                              throws java.io.IOException
```

:   Averages the energy output files from individual simulations.

    :   **Parameters:**: `iterations` - The number of simulations to average: `OUTPUT_FILE_LOC` - The prefix for the output file location: `STD_FILE_LOC` - The standard deviation output file location: `AVG_FILE_LOC` - THe average output file location: `num_of_time_points` - The number of timepoints in each simulation **Throws:**: `java.io.IOException`

---


### combineInitRnaData

```
public static void combineInitRnaData(int iterations,
                                      java.lang.String OUTPUT_FILE_LOC,
                                      java.lang.String STD_FILE_LOC,
                                      java.lang.String AVG_FILE_LOC,
                                      int num_of_time_points)
                               throws java.io.IOException
```

:   Averages the RNA initiation output files from individual simulations.

    :   **Parameters:**: `iterations` - The number of simulations to average: `OUTPUT_FILE_LOC` - The prefix for the output file location: `STD_FILE_LOC` - The standard deviation output file location: `AVG_FILE_LOC` - THe average output file location: `num_of_time_points` - The number of timepoints in each simulation **Throws:**: `java.io.IOException`

---


### combineData

```
public static int combineData(int iterations,
                              java.lang.String OUTPUT_FILE_LOC,
                              java.lang.String STD_FILE_LOC,
                              java.lang.String AVG_FILE_LOC)
                       throws java.io.IOException
```

:   Averages the molecule file data across multiple simulations

    :   **Parameters:**: `iterations` - The number of simulations to average: `OUTPUT_FILE_LOC` - The prefix for the output file loation: `STD_FILE_LOC` - The location of the standard deviation output file: `AVG_FILE_LOC` - The location of the average output file **Throws:**: `java.io.IOException`


---


|  |  |  |  |  |  |  |  |  |  |  |
| --- | --- | --- | --- | --- | --- | --- | --- | --- | --- | --- |
| |  |  |  |  |  |  |  | | --- | --- | --- | --- | --- | --- | --- | | Package | | **Class** | **Tree** | **Deprecated** | **Index** | **Help** | | | |  |
| **PREV CLASS**   **NEXT CLASS** | **FRAMES**    **NO FRAMES**     **All Classes** |
| SUMMARY: NESTED | FIELD | CONSTR | METHOD | DETAIL: FIELD | CONSTR | METHOD |


---
