# Supplementary material for: TABASCO: A single molecule, base-pair resolved gene expression simulator
Source: BMC Bioinformatics. 2007 Dec 19;8:480. doi: 10.1186/1471-2105-8-480 (PMC2242808; doi:10.1186/1471-2105-8-480)
Supplement: Additional File 3 — TABASCO website. [file 1471-2105-8-480-S3.zip › doc/TabascoWrite.html]

TabascoWrite


|  |  |  |  |  |  |  |  |  |  |  |
| --- | --- | --- | --- | --- | --- | --- | --- | --- | --- | --- |
| |  |  |  |  |  |  |  | | --- | --- | --- | --- | --- | --- | --- | | Package | | **Class** | **Tree** | **Deprecated** | **Index** | **Help** | | | |  |
| **PREV CLASS**   **NEXT CLASS** | **FRAMES**    **NO FRAMES**     **All Classes** |
| SUMMARY: NESTED | FIELD | CONSTR | METHOD | DETAIL: FIELD | CONSTR | METHOD |


---


## Class TabascoWrite

```
java.lang.Object
  TabascoWrite
```

---

public class **TabascoWrite** extends java.lang.Object

TabascoWrite is the class that is used to write DNA output files of simulations.

---

|  |  |
| --- | --- |
| **Constructor Summary** | |
| `TabascoWrite(java.lang.String filename)` |


|  |  |
| --- | --- |
| **Method Summary** | |
| `void` | `cleanUp()` |
| `void` | `writeComplexes(DPComplex[] DNAComplexArray)` |
| `void` | `writeDNAFeatures(short[] DNAFeatures)` |
| `void` | `writeEntry(int entry)` |
| `void` | `writeFirstLine(Phage thephage)` |
| `void` | `writeGenomeSize(int GenomeSize)` |
| `void` | `writeMainLine(double time, Phage thephage, int whichphage)` |
| `void` | `writeMRNA(Molecule[] MoleculesArray, Phage thephage, int lastProtID, int whichPhage, int numOfCodingRgn)` |
| `void` | `writeNumberOfCodingRgns(int numOfCodingRgn)` |
| `void` | `writeNumberOfTimepoints(int numOfTimePoints)` |
| `void` | `writeTime(double time)` |
| `void` | `writeTimeEnd(int timeEnd)` |
| `void` | `writeTimeStep(int timeStep)` |

|  |
| --- |
| **Methods inherited from class java.lang.Object** |
| `clone, equals, finalize, getClass, hashCode, notify, notifyAll, toString, wait, wait, wait` |

|  |
| --- |
| **Constructor Detail** |

### TabascoWrite

```
public TabascoWrite(java.lang.String filename)
             throws java.io.IOException
```


|  |
| --- |
| **Method Detail** |

### writeFirstLine

```
public void writeFirstLine(Phage thephage)
                    throws java.io.IOException
```

:   **Throws:**: `java.io.IOException`

---


### writeMainLine

```
public void writeMainLine(double time,
                          Phage thephage,
                          int whichphage)
                   throws java.io.IOException
```

:   **Throws:**: `java.io.IOException`

---


### writeNumberOfCodingRgns

```
public void writeNumberOfCodingRgns(int numOfCodingRgn)
                             throws java.io.IOException
```

:   **Throws:**: `java.io.IOException`

---


### writeTimeStep

```
public void writeTimeStep(int timeStep)
                   throws java.io.IOException
```

:   **Throws:**: `java.io.IOException`

---


### writeTimeEnd

```
public void writeTimeEnd(int timeEnd)
                  throws java.io.IOException
```

:   **Throws:**: `java.io.IOException`

---


### writeGenomeSize

```
public void writeGenomeSize(int GenomeSize)
                     throws java.io.IOException
```

:   **Throws:**: `java.io.IOException`

---


### writeNumberOfTimepoints

```
public void writeNumberOfTimepoints(int numOfTimePoints)
                             throws java.io.IOException
```

:   **Throws:**: `java.io.IOException`

---


### writeTime

```
public void writeTime(double time)
               throws java.io.IOException
```

:   **Throws:**: `java.io.IOException`

---


### writeEntry

```
public void writeEntry(int entry)
                throws java.io.IOException
```

:   **Throws:**: `java.io.IOException`

---


### writeComplexes

```
public void writeComplexes(DPComplex[] DNAComplexArray)
                    throws java.io.IOException
```

:   **Throws:**: `java.io.IOException`

---


### writeDNAFeatures

```
public void writeDNAFeatures(short[] DNAFeatures)
                      throws java.io.IOException
```

:   **Throws:**: `java.io.IOException`

---


### writeMRNA

```
public void writeMRNA(Molecule[] MoleculesArray,
                      Phage thephage,
                      int lastProtID,
                      int whichPhage,
                      int numOfCodingRgn)
               throws java.io.IOException
```

:   **Throws:**: `java.io.IOException`

---


### cleanUp

```
public void cleanUp()
             throws java.io.IOException
```

:   **Throws:**: `java.io.IOException`


---


|  |  |  |  |  |  |  |  |  |  |  |
| --- | --- | --- | --- | --- | --- | --- | --- | --- | --- | --- |
| |  |  |  |  |  |  |  | | --- | --- | --- | --- | --- | --- | --- | | Package | | **Class** | **Tree** | **Deprecated** | **Index** | **Help** | | | |  |
| **PREV CLASS**   **NEXT CLASS** | **FRAMES**    **NO FRAMES**     **All Classes** |
| SUMMARY: NESTED | FIELD | CONSTR | METHOD | DETAIL: FIELD | CONSTR | METHOD |


---
