# Supplementary material for: TABASCO: A single molecule, base-pair resolved gene expression simulator
Source: BMC Bioinformatics. 2007 Dec 19;8:480. doi: 10.1186/1471-2105-8-480 (PMC2242808; doi:10.1186/1471-2105-8-480)
Supplement: Additional File 3 — TABASCO website. [file 1471-2105-8-480-S3.zip › doc/TabascoXML.html]

TabascoXML


|  |  |  |  |  |  |  |  |  |  |  |
| --- | --- | --- | --- | --- | --- | --- | --- | --- | --- | --- |
| |  |  |  |  |  |  |  | | --- | --- | --- | --- | --- | --- | --- | | Package | | **Class** | **Tree** | **Deprecated** | **Index** | **Help** | | | |  |
| **PREV CLASS**   **NEXT CLASS** | **FRAMES**    **NO FRAMES**     **All Classes** |
| SUMMARY: NESTED | FIELD | CONSTR | METHOD | DETAIL: FIELD | CONSTR | METHOD |


---


## Class TabascoXML

```
java.lang.Object
  TabascoXML
```

---

public class **TabascoXML** extends java.lang.Object

TabascoXML parses the XML input file that specifies simulation parameters

---

|  |  |
| --- | --- |
| **Constructor Summary** | |
| `TabascoXML()` |


|  |  |
| --- | --- |
| **Method Summary** | |
| `static Reaction[]` | `fillCell(Cell myCell, XMLObject simxmo, int reactionid)`             Parses parameters to initialize the Cell class |
| `static void` | `fillPhage(Cell myCell, Phage myPhage, XMLObject phagexmo, int moleculeID)`             Parses parameters to initialize the Phage class |
| `static Reaction[]` | `fillPhageReactions(Phage myPhage, int reactionid, int GJRNAINDEX)`             Parses parameters to fill in reactions from phage class |
| `static int` | `fillSim(TabascoSimulator mySim, XMLObject simxmo)`             Parses parameters to initialize the Simulator class |

|  |
| --- |
| **Methods inherited from class java.lang.Object** |
| `clone, equals, finalize, getClass, hashCode, notify, notifyAll, toString, wait, wait, wait` |

|  |
| --- |
| **Constructor Detail** |

### TabascoXML

```
public TabascoXML()
```


|  |
| --- |
| **Method Detail** |

### fillSim

```
public static int fillSim(TabascoSimulator mySim,
                          XMLObject simxmo)
                   throws java.io.IOException
```

:   Parses parameters to initialize the Simulator class

    :   **Parameters:**: `mySim` - A pointer to the simulation class to initialize: `simxmo` - The XML object from which to parse information **Throws:**: `java.io.IOException`

---


### fillCell

```
public static Reaction[] fillCell(Cell myCell,
                                  XMLObject simxmo,
                                  int reactionid)
                           throws java.io.IOException
```

:   Parses parameters to initialize the Cell class

    :   **Parameters:**: `myCell` - A pointer to the cell class to initialize: `simxmo` - The XML object from which to parse information: `reactionid` - The reactionID to begin adding reactions **Returns:**: A vector containing reactions to add to the simulation class. **Throws:**: `java.io.IOException`

---


### fillPhage

```
public static void fillPhage(Cell myCell,
                             Phage myPhage,
                             XMLObject phagexmo,
                             int moleculeID)
                      throws java.io.IOException
```

:   Parses parameters to initialize the Phage class

    :   **Parameters:**: `myCell` - A pointer to the cell class the phage is contained within: `myPhage` - A pointer to the phage class to initialize: `phagexmo` - The XML object from which to parse information: `moleculeID` - The moleculeID to begin adding molecules (Cell class already added molecules) **Throws:**: `java.io.IOException`

---


### fillPhageReactions

```
public static Reaction[] fillPhageReactions(Phage myPhage,
                                            int reactionid,
                                            int GJRNAINDEX)
```

:   Parses parameters to fill in reactions from phage class

    :   **Parameters:**: `myPhage` - A pointer to the phage that is being initialized: `reactionid` - The reactionID to add reactions from.: `GJRNAINDEX` - The index in the molecules vector that the GammaJumpRNA start. **Returns:**: A vector containing reactions to add to the simulation class.


---


|  |  |  |  |  |  |  |  |  |  |  |
| --- | --- | --- | --- | --- | --- | --- | --- | --- | --- | --- |
| |  |  |  |  |  |  |  | | --- | --- | --- | --- | --- | --- | --- | | Package | | **Class** | **Tree** | **Deprecated** | **Index** | **Help** | | | |  |
| **PREV CLASS**   **NEXT CLASS** | **FRAMES**    **NO FRAMES**     **All Classes** |
| SUMMARY: NESTED | FIELD | CONSTR | METHOD | DETAIL: FIELD | CONSTR | METHOD |


---
