# Supplementary material for: TABASCO: A single molecule, base-pair resolved gene expression simulator
Source: BMC Bioinformatics. 2007 Dec 19;8:480. doi: 10.1186/1471-2105-8-480 (PMC2242808; doi:10.1186/1471-2105-8-480)
Supplement: Additional File 3 — TABASCO website. [file 1471-2105-8-480-S3.zip › doc/XMLObject.html]

XMLObject


|  |  |  |  |  |  |  |  |  |  |  |
| --- | --- | --- | --- | --- | --- | --- | --- | --- | --- | --- |
| |  |  |  |  |  |  |  | | --- | --- | --- | --- | --- | --- | --- | | Package | | **Class** | **Tree** | **Deprecated** | **Index** | **Help** | | | |  |
| **PREV CLASS**   NEXT CLASS | **FRAMES**    **NO FRAMES**     **All Classes** |
| SUMMARY: NESTED | FIELD | CONSTR | METHOD | DETAIL: FIELD | CONSTR | METHOD |


---


## Class XMLObject

```
java.lang.Object
  XMLObject
```

---

public class **XMLObject** extends java.lang.Object

---

|  |  |
| --- | --- |
| **Field Summary** | |
| `java.util.Properties` | `attributes` |
| `java.util.Vector` | `childrenNodes` |
| `java.lang.String` | `contents` |
| `java.lang.String` | `name` |


|  |  |
| --- | --- |
| **Constructor Summary** | |
| `XMLObject()` |
| `XMLObject(java.io.File file)` |
| `XMLObject(java.lang.String xml)`             Main constructor for an XML Object, takes the XML text and creates an XML Object encapsulating and enabling access to taht data |


|  |  |
| --- | --- |
| **Method Summary** | |
| `void` | `addChild(java.lang.String xml)`             Adds a child node |
| `void` | `addChild(XMLObject child)`             Adds a child node |
| `java.lang.String` | `getAttribute(java.lang.String key)`             Returns the value of the attribute with the key given |
| `java.util.Properties` | `getAttributes()`             Returns the attributes of this XML Object |
| `XMLObject` | `getChild(java.lang.String blockHeader)` |
| `java.util.Vector` | `getChildren()`             Returns the XML children nodes for this object |
| `java.lang.String` | `getContents()` |
| `java.lang.String` | `getName()`             Returns the name of this XML Object |
| `java.util.Vector` | `getNamedChildren(java.lang.String name)` |
| `java.util.Properties` | `getNVPairs(java.lang.String attrTag, java.lang.String nameTag, java.lang.String valTag)` |
| `java.lang.String` | `getProperty(java.lang.String key)` |
| `java.lang.String` | `print()`             Returns a string containing the XML text representation of this object |
| `void` | `removeAttribute(java.lang.String key)`             Removes an attribute with the key given |
| `void` | `setAttribute(java.lang.String key, java.lang.String value)`             Sets an Attribute for the XML object |
| `void` | `setAttributes(java.util.Properties atts)` |
| `void` | `setContents(java.lang.String ct)` |
| `void` | `setName(java.lang.String n)`             Sets the name of this XML Object |
| `void` | `setProperty(java.lang.String key, java.lang.String value)` |

|  |
| --- |
| **Methods inherited from class java.lang.Object** |
| `clone, equals, finalize, getClass, hashCode, notify, notifyAll, toString, wait, wait, wait` |

|  |
| --- |
| **Field Detail** |

### childrenNodes

```
public java.util.Vector childrenNodes
```

---


### attributes

```
public java.util.Properties attributes
```

---


### name

```
public java.lang.String name
```

---


### contents

```
public java.lang.String contents
```


|  |
| --- |
| **Constructor Detail** |

### XMLObject

```
public XMLObject()
```

---


### XMLObject

```
public XMLObject(java.lang.String xml)
```

:   Main constructor for an XML Object, takes the XML text and creates an XML Object
    encapsulating and enabling access to taht data

    **Parameters:**: `xml` - The XML string that will form the XML Object

---


### XMLObject

```
public XMLObject(java.io.File file)
```


|  |
| --- |
| **Method Detail** |

### setContents

```
public void setContents(java.lang.String ct)
```

---


### getContents

```
public java.lang.String getContents()
```

---


### getChildren

```
public java.util.Vector getChildren()
```

:   Returns the XML children nodes for this object

---


### addChild

```
public void addChild(XMLObject child)
```

:   Adds a child node

    :   **Parameters:**: `child` - The child node to add

---


### addChild

```
public void addChild(java.lang.String xml)
```

:   Adds a child node

---


### getAttributes

```
public java.util.Properties getAttributes()
```

:   Returns the attributes of this XML Object

---


### setAttributes

```
public void setAttributes(java.util.Properties atts)
```

---


### getName

```
public java.lang.String getName()
```

:   Returns the name of this XML Object

---


### setName

```
public void setName(java.lang.String n)
```

:   Sets the name of this XML Object

    :   **Parameters:**: `n` - The name to assign to the object

---


### setAttribute

```
public void setAttribute(java.lang.String key,
                         java.lang.String value)
```

:   Sets an Attribute for the XML object

    :   **Parameters:**: `key` - the name of the attribute to set: `value` - the value of the attribute

---


### setProperty

```
public void setProperty(java.lang.String key,
                        java.lang.String value)
```

---


### removeAttribute

```
public void removeAttribute(java.lang.String key)
```

:   Removes an attribute with the key given

    :   **Parameters:**: `key` - the name of the attribute to remove

---


### getAttribute

```
public java.lang.String getAttribute(java.lang.String key)
```

:   Returns the value of the attribute with the key given

    :   **Parameters:**: `key` - the name of the attribute to get

---


### getProperty

```
public java.lang.String getProperty(java.lang.String key)
```

---


### print

```
public java.lang.String print()
```

:   Returns a string containing the XML text representation of this object

---


### getChild

```
public XMLObject getChild(java.lang.String blockHeader)
```

---


### getNamedChildren

```
public java.util.Vector getNamedChildren(java.lang.String name)
```

---


### getNVPairs

```
public java.util.Properties getNVPairs(java.lang.String attrTag,
                                       java.lang.String nameTag,
                                       java.lang.String valTag)
```


---


|  |  |  |  |  |  |  |  |  |  |  |
| --- | --- | --- | --- | --- | --- | --- | --- | --- | --- | --- |
| |  |  |  |  |  |  |  | | --- | --- | --- | --- | --- | --- | --- | | Package | | **Class** | **Tree** | **Deprecated** | **Index** | **Help** | | | |  |
| **PREV CLASS**   NEXT CLASS | **FRAMES**    **NO FRAMES**     **All Classes** |
| SUMMARY: NESTED | FIELD | CONSTR | METHOD | DETAIL: FIELD | CONSTR | METHOD |


---
