# Supplementary material for: TABASCO: A single molecule, base-pair resolved gene expression simulator
Source: BMC Bioinformatics. 2007 Dec 19;8:480. doi: 10.1186/1471-2105-8-480 (PMC2242808; doi:10.1186/1471-2105-8-480)
Supplement: Additional File 3 — TABASCO website. [file 1471-2105-8-480-S3.zip › README.html]

TABASCO - OpenWetWare

# TABASCO

### From OpenWetWare

Jump to: navigation, search

**Home**        
  **Contact**        
  **Internal**        
  **Lab Members**        
  **Publications**        
  **Research**        
  **Talks**

  

|  |
| --- |
| Contents[hide]   - 1 Summary - 2 People - 3 Methods - 4 Software and Installation   - 4.1 Software   - 4.2 Installation instructions (for Unix systems) - 5 Usage   - 5.1 TabascoSimulator   - 5.2 Averager   - 5.3 TabascoJpegMake - 6 Examples |

## [edit] Summary

Tabasco is a simulator created to address the problem of simulating
gene expression at single-base resolution. By defining the logic of
transcription and translation rules a priori such as initiation,
elongation, termination, and interactions of polymerases and proteins,
Tabasco automatically traverses the state of the system as it develops
and thus makes simulation at such high resolution computationally
feasible. Tabasco was designed to allow us to better understand
bacteriophage gene expression. In general, Tabasco would be useful to
those interested in explicitly simulating hypotheses of protein-DNA
interactions and their relation to gene expression (e.g., eukaryotic
gene expression initiation).

## [edit] People

Sriram Kosuri,
Jason Kelly,
Drew Endy

## [edit] Methods

Tabasco avoids previous ‘combinatorial explosion’ problems by
tracking the position and state of proteins and genetic elements on the
DNA to dynamically generate appropriate reactions, such as promoters
being blocked by traversing polymerases. In order to improve
computational efficiency, Tabasco makes use of a Gibson-accelerated
Gillespie SSA to compute the reaction event timing and the resultant
time-evolution of the genetic system.

## [edit] Software and Installation

### [edit] Software

The software for Tabasco is freely available here:  
Extractable archive of all code, documentation, and examples   
Java Source Files   
Javadoc Documentation   
Compiled Java Byte Code

The simulator was only tested on J2SE version 1.4.2
and higher. A basic knowledge of running Java programs is needed.
Generally one can either download the source, and then compile it using
a java compiler to create byte code or download the precompiled byte
code above.

### [edit] Installation instructions (for Unix systems)

1. Download the extractable archive.
2. Extract the archive using the following command:

   ```
    tar -xvzf tabasco.tar.gz
   ```

   - Windows users should be able to use WinZip or other free programs (or download Cygwin to make the process more unix like)
3. Make sure java is in your path
   - To check, type

     ```
     java -version
     ```

     and make sure the
     command is found (also a good time to make sure you are above version
     1.4.2). If you do not have an up-to-date version of Java, visit the download site and follow the installation instructions.

## [edit] Usage

There are 3 main classes that can be run. TabascoSimulator.class
actually executes the simulation and is the longest program.
Averager.class averages output from multiple TabascoSimulator
simulations. TabascoJpegMake is the visualizer that makes JPEG stacks
to visualize data that can later be made into movies.

### [edit] TabascoSimulator

The TabascoSimulator class executes simulations. The basic usage is as follows.

```
java TabascoSimulator inputfilename outputfilename [random seed]
```

- The inputfilename is the location of the location of the input file.
- The outputfilename is the prefix to be used for the output files.

The optional random seed can be used to override the input from the
inputfilename. This field is useful if one is using a script to run
simulations on a cluster.

A sample input file is included in the distrubution. To run it, follow this procedure:

1. Go to the TabascoWeb/classes directory by typing

   ```
   cd TabascoWeb/classes
   ```
2. Try to run a sample program

   ```
   java TabascoSimulator ../examples/t7_input_file.txt ../output-test-
   ```

### [edit] Averager

The Averager class averages output from the TabascoSimulator. The basic usage is as follows.

```
java Averager #_of_iterations outputfilename
```

- The #\_of\_iterations is an integer that specifies the number of simulations you want to average together
- The outputfilename is the prefix to be used for the output files.

The example simulation run in TabascoSimulator can be averaged as follows:

```
java Averager 2 ../output-test-
```

### [edit] TabascoJpegMake

This class makes a set of jpeg's from the output files to visualize the DNA and molecule levels as a function of time.

```
java TabascoJpegMake output-filename mol-input-filename dna-file1 dna file2 ...
```

- output-filename is the filename prefix for jpeg's that are output
- mol-input-filename is the Molecule file name that you want to visualize
- dna-file1 and dna-file2, etc are the DNA files that will also be a part of the visualization.

For the example input, you can visualize the output by running the following command.

```
java TabascoJpegMake ../vis- ../output_AVG.txt ../output-test-DNA_phage1_sim1.txt   
 ../output-test-DNA_phage2_sim1.txt ../output-test-DNA_phage3_sim1.txt
```

## [edit] Examples

QuickTime movie showing TABASCO being used to simulate gene expression for the first 1500 seconds of bacteriophage T7 development.


Retrieved from "http://openwetware.org/wiki/TABASCO"

##### Views

- Article
- Discussion
- Edit
- History

##### Personal tools

- Log in / create account

##### Navigation

- Main Page
- Community portal
- Recent changes
- Random page

##### resources

- Materials
- Protocols
- Help

##### Search

##### Toolbox

- What links here
- Related changes
- Upload file
- Special pages
- Printable version
- Permanent link
- Cite this article

- This page was last modified 16:49, 3 May 2007.
- This page has been accessed 3,948 times.
- Content is available under GNU FDL or Creative Commons BY-SA.
- Privacy policy
- About OpenWetWare
- Disclaimers
