# Supplementary figures and images for: TABASCO: A single molecule, base-pair resolved gene expression simulator
Source: BMC Bioinformatics. 2007 Dec 19;8:480. doi: 10.1186/1471-2105-8-480 (PMC2242808; doi:10.1186/1471-2105-8-480)

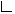

Supplement: Additional File 3 — TABASCO website. [file 1471-2105-8-480-S3.zip › doc/resources/inherit.gif]
