# Supplementary material for: TABASCO: A single molecule, base-pair resolved gene expression simulator
Source: BMC Bioinformatics. 2007 Dec 19;8:480. doi: 10.1186/1471-2105-8-480 (PMC2242808; doi:10.1186/1471-2105-8-480)
Supplement: Additional File 4 — Parameter definitions within the input file. The table describes the parameter definitions that are used in the input files that are contained in the supplementary materials. [file 1471-2105-8-480-S4.doc]

**Table S1. Parameter definitions within the input file.** The table describes the parameter definitions that are used in the input files that are contained in the supplementary materials.

| Parameter | Meaning |
| --- | --- |
| **Simulation Parameters** | |
| **General Notes** | Contained within the Simulation heading is all information regarding the simulation to be run. Under the Simulation heading, multiple Cell’s can be parameterized. |
| *runs* | The number of runs of the current simulation that will be run in series by Tabasco. The random seed is incremented with each successive run. |
| *random seed* | The random seed the simulation uses to run a particular simulation. This is to ensure repeatability of the simulation being run. |
| *simulation name* | Internal variable that is used to keep track of names in case multiple simulations are run simultaneously. |
| *time step* | The increment (in seconds) that the simulator will output data. |
| *time end* | The time at which simulation will exit (in seconds). |
| *write DNA* | (yes or no); Whether or not to output locations of all complexes at each DNA position for each molecule of DNA for each timepoint; slows down simulation significantly. This is mostly used for downstream imaging purposes. |
| *write Init RNA* | (yes or no); Whether or not to output initiation rates at each promoter for each timepoint; used for tracking and debugging purposes. |
| *write Energy* | (yes or no); Whether or not to output amino and nucleic acid usages for each timepoint; used for tracking and debugging purposes. |
| *compDeg* | (yes or no); Yes specifies that DNA degradation rates refer to a DNA degradation mechanism where degradation machinery competes with ribosomal binding for binding the 5’ end of mRNA; No specifies that there is no such competition for the degradation machinery. |
| **Cell Parameters** | |
| **General Notes** | Each Cell can contain one or many Ribosome, Polymerase, Species, Reaction, and DNA System parameterizations. |
| *volume* | The volume of the cell in the simulation. This is used to calculate microscopic rate constants from mesoscopic rate constants. |
| *polymerase interaction model* | (TRAFFIC JAM, UPSTREAM FALLOFF, DOWNSTREAM FALLOFF, CUSTOM); Specifies the way polymerases interact on the DNA with one another. The first three models are described in the text. The CUSTOM option is uses a table directly in the source code that can be edited to specify how different polymerases or proteins interact with each other. |
| **Ribosome Parameters** | |
| *n* | The number of free ribosomes in the cell at the start of simulation. The ribosomes in the cell can change if a reaction is defined to make new ribosomes. |
| *id* | The ID number of the ribosome for the purposes of identification in the Reaction and RBS definition sections (below). |
| *speed* | The rate at which the ribosome traverses mRNA at bases per second. |
| *footprint* | The number of bases of RNA the ribosome covers; this parameter is used to calculate ribosome binding site clearance, as well as if too many ribosomes have loaded a particular Nascent RNA (Methods). |
| **Polymerase Parameters** | |
| *n* | The number of initial polymerase molecules. |
| *speed* | The rate of polymerase elongation in base pairs per second. |
| *id* | The protein id of this polymerase for the purposes of identification in the Reaction and RBS definition sections (below). |
| *organism id* | Used as a parameter for visualization of DNA dynamics; each number will be grouped by color in the visualization. |
| *footprint* | The number of bases the polymerase occupies. |
| *name* | The name of the polymerase for output purposes. |
| **Species Parameters** | |
| *name* | The name of the species for output purposes. |
| *n* | The number of initial species at the start of simulation. |
| *ID* | The protein id of this species for the purposes of identification in the Reaction and RBS definition sections (below). |
| *organism* | No longer used; The value of this parameter does not matter; Previously used for visualization purposes. |
| **Reaction Parameters** | |
| **General Notes** | The specified reactions are for those not automatically generated by the simulator. These reactions are in the form of ID1+ID2>ID3+ID4;Reaction Rate. The Reaction Rate is a mesoscopic rate constant with appropriate units to the number of reactants. The IDX refer to the ID’s of the Species, Polymerases, or Ribosomes specified above. For this reason, those ID’s cannot overlap. |
| **DNA System** | |
| **General Notes** | Each DNA System describes a particular molecule of DNA (each can have many copies so as to not re-parameterize each one). Each DNA System can have Promoters, Terminators, and RBS. |
| name | The name of the DNA molecules; Used for output purposes. |
| *genome length* | The length of the genome in base pairs. |
| *copy number* | The number of copies of this DNA system within the Cell. |
| *entry rate constant* | The rate in base pairs per second at which DNA enters the cell through the polymerase independent mechanisms. To allow for instantaneous entry, this rate can be set very large. |
| *entry offsite* | The position in which the polymerase independent entry rate will no longer allow DNA entry. |
| **Promoter** | |
| **General Notes** | Describes each promoter on the DNA. Each Promoter will have one or many Polymerase entries that describe the promoter constants for each specified Polymerase. |
| *start* | The 5’ position of the promoter site; used for determining when the proteins may obscure polymerase binding. |
| *stop* | The 3’ position of the promoter site; used for determining when the proteins may obscure polymerase binding. |
| *startsite* | The position where mRNA polymerization begins. |
| *name* | The name of the promoter for output purposes. |
| *organism id* | Used for coloring in the visualization just like the polymerases. |
| **Polymerase (within Promoter) Parameters** | |
| *polymerase ID* | The Polymerase ID (from above) of the polymerase that binds this promoter. |
| *aon* | rate of polymerase-promoter binding (M^-1^s^-1^). |
| *aoff* | rate of polymerase-promoter unbinding (s^-1^). |
| *ainiton* | rate of initiation complex formation (s^-1^). |
| *runoff percentage* | Percentage of initiation complexes that form elongation complexes (0-100). |
| *aelong* | rate of elongation complex formation (s^-1^). |
| *arecyc* | rate constant for recycling back to initially bound promoter state (s^-1^). |
| **Terminator Parameters** | |
| **General Notes** | Describes each transcriptional terminator on the DNA. Each Terminator will have one or many Polymerase entries that describe the termination efficiencies of each Polymerase that passes the Terminator. |
| *start* | 5’ position of terminator region. |
| *stop* | 3’ position of terminator region. |
| *stopsite* | The site of the polymerase stochastic decision to terminate transcription. |
| *name* | The name of the terminator for output purposes. |
| *organism* | No longer used; The value of this parameter does not matter; Previously used for visualization purposes. |
| **Polymerase (within Terminators) Parameters** | |
| *ID* | The Polymerase ID (from above) that the following parameters correspond to |
| *efficiency* | Termination efficiency from 0 to 1. 0 describes complete pass through, while 1 described 100% termination efficiency. |
| **RBS parameters** | |
| **General Notes** | Describes each ribosome binding site on the DNA system. |
| *start* | 5’ position of the RBS. |
| *stop* | 3’ position of the RBS. |
| *startsite* | The position of the start codon. |
| *initstepsize* | The initial step size that determines the number of bases of RNA the ribosome must traverse to clear the ribosome binding site. |
| *elongstepsize* | No longer used; The value of this parameter does not matter; Now calculated automatically using the stopsite and the initstepsize. |
| *strength* | The second order rate constant (M^-1^s^-1^) for ribosome binding to the RBS. |
| *stopsite* | The stopsite of the reading frame described by this RBS. |
| *protid* | The ID of the protein that is produced (from above). |
| *initRateConstant* | The rate constant for clearing the ribosome binding site (s^-1^). |
| *mrnadeg* | The rate constant for mRNA degradation for this mRNA molecule (s^-1^). |
